# Intervention Mapping: A Framework to Co‐Design the ALAPAGE Programme to Simultaneously Improve Dietary Diversity and Physical Fitness Among Older People

**DOI:** 10.1111/hex.70612

**Published:** 2026-03-23

**Authors:** Anne‐Fleur Jacquemot, Aurélie Bocquier, Agnès Vinet, Christophe Dubois, Nicole Darmon, Florence Cousson Gélie, Catherine Féart, Pierre Verger, Sarah Danthony, The ALAPAGE Study Group, Valérie Arquier, Guillaume Briclot, Rachel Chamla, Karin Delrieu, Julie Dessirier, Catherine Féart, Christine Fusinati, Rozenn Gazan, Mélissa Gibert, Valérie Lamiraud, Matthieu Maillot, Dolorès Nadal, Christelle Trotta, Eric O. Verger, Valérie Viriot

**Affiliations:** ^1^ Bordeaux Population Health Research Centre, University of Bordeaux, Inserm, UMR 1219 Bordeaux France; ^2^ Observatoire régional de la santé Marseille France; ^3^ Université de Lorraine, Inserm, INSPIIRE Nancy France; ^4^ Avignon Université, UPR4278 Avignon France; ^5^ Trophis Les Pennes Mirabeau France; ^6^ MoISA, Université de Montpellier, CIHEAM‐IAMM, CIRAD, INRAE, Institut Agro, IRD Montpellier France; ^7^ Prevention Department Epidaure Institut du Cancer de Montpellier Montpellier France; ^8^ EPSYLON EA 4556, Université Paul Valéry Montpellier 3 Montpellier France; ^9^ Laboratoire SENS Université Grenoble Alpes Grenoble France; ^10^ Caisse d'assurance retraite et de la santé au travail Sud‐Est (Carsat Sud‐Est) Marseille France; ^11^ Association Géront'ONord Pôle Infos Séniors Marseille Nord Marseille France; ^12^ Mutualité Française Sud Provence‐Alpes‐Côte d'Azur Marseille France; ^13^ MS‐Nutrition Marseille France; ^14^ SudEval Provence‐Alpes‐Côte d'Azur Corse Marseille France; ^15^ Association de Santé, d'Éducation et de Prévention sur les Territoires Provence‐Alpes‐Côte d'Azur (ASEPT PACA) Marseille France

**Keywords:** codesign, dietary diversity, intervention, intervention mapping, older adults, physical activity, seniors

## Abstract

**Background:**

The design process of public health promotion programmes is rarely described in detail. This study aims to present the co‐design process and theoretical rationale of a diet‐ and physical activity‐promoting programme called ALAPAGE, targeting community‐dwelling adults aged 60 years and older in southeastern France.

**Methods:**

The six steps of the Intervention Mapping (IM) framework applied to the ALAPAGE programme were as follows:

1. Needs assessment: Eighteen participatory meetings with stakeholders and a literature review were conducted to identify determinants of behaviour change.
2. Identification of change objectives (immediate targets of the ALAPAGE programme).
3. Intervention theory: Based on the literature and health psychology expertise, Theory of Planned Behaviour and Goal Setting Theory were used supplemented by behaviour change techniques. These were adapted to the change objectives and translated into practical strategies.
4. Development of the ALAPAGE programme sequences.

Steps 5 (implementation) and 6 (evaluation) are described in a separate article.

**Results:**

Following IM, 31 change objectives were defined, adapted to 15 determinants of behaviour change (e.g., setting a personalised goal was selected as a change objective to address the determinant ‘experimenting with behaviour’). Thirty practical strategies (e.g., group discussions) were organised into seven group sessions. Nineteen pedagogical tools (e.g., 24‐h recall sheets) were tested in a pilot study involving 21 participants.

**Conclusion:**

Using the IM approach, the ALAPAGE programme integrates behaviour change techniques to simultaneously address determinants of diet and physical activity among seniors.

**Public Contribution:**

Professionals from partner organisations contributed to needs assessment, and programme development. Dieticians and physical activity specialists helped identify behaviour change determinants, validated the theoretical model, and participated in programme design and testing. Seniors were interviewed during the needs assessment and tested the programme sequence.

**Trial Registration:**

ClinicalTrials.gov identifier: NCT05 140330, 1 December 2021, https://www.clinicaltrials.gov/study/NCT05140330?titles=ALAPAGE&rank=1.

**Abbreviations**
IM: intervention mapping
PA: physical activity
Pro‐APA: qualified adapted physical activity professional

## 1 Background

Due to increasing life expectancy, the proportion of seniors in the population is rising [1]. In France, in 2019, adults aged 65 years and older represented 20.5% of the population, and this percentage is expected to reach 28.7% in 2070 [2]. As a consequence, the prevalence of age‐related chronic illnesses is increasing [3]. A healthy diet and physical activity (PA) throughout the life may delay the occurrence of chronic diseases in later life [4, 5]. The WHO recommends that seniors engage at least 150 min of moderate‐to‐vigorous PA per week. It also advises adopting a healthy diet, including increased consumption of fruits, vegetables, dairy products, and protein‐rich foods [6].

In France, 95% of seniors live at home and57% of seniors are women [2]. In 2020, the standard of living for seniors was slightly higher than younger people [2]. Moreover 30% of people aged 75 and over were users of digital material, highlighting the persistence of a digital divide among seniors [7].

To improve the health of seniors, some diet and PA programmes have been developed and evaluated worldwide [8, 9]. However, these programmes are often poorly described, which limits their transferability, and many are associated with high attrition rates [10, 11]. Recent recommendations for the development of such programmes emphasis the use of theoretical models and behaviour change techniques (i.e. general techniques or processes for influencing changes in the determinants of behaviours and environmental conditions [12]), as well as the involvement of stakeholders to support successful implementation [9] and to enhance effectiveness and participant retention [11]. Moreover, providing a detailed description of intervention development is recommended to facilitate transferability and to support both researchers and public health professionals in designing their own programmes [13]. In France, as part of the national strategy for healthy ageing, several national and regional stakeholders (e.g. the French Public Health Agency, retirement funds, and community centres involved in health promotion) have supported field actions targeting seniors' diet and PA for many years (most often as group workshops). However, these field initiatives present several limitations (e.g. heterogeneity in objectives), and to date no study has assessed their effectiveness [14]. In this context, ALAPAGE (ALimentation et Activité Physique chez les personnes ÂGEes) research was initiated 1) to co‐design a dietary and PA health promotion programme (the ALAPAGE programme) for community dwellers aged over 60 years and still autonomous; and 2) to evaluate the effectiveness and implementation of this programme in the Provence‐Alpes‐Côte d'Azur region (i.e. southeastern France). The objective of this article is to describe the ALAPAGE programme's co‐design process.

## 2 Methods

We used the intervention mapping (IM), created in 1998 and enhanced in 2006 by Bartholomew et al. [15]. It is a framework of six steps to ‘make and document decisions about how to influence change in behaviour and conditions to promote health and to prevent or improve a health problem’ [15].

To develop the ALAPAGE programme, we applied IM steps 1 to 4 (Table 1). IM steps 5 and 6 which focus on programme implementation and evaluation have been fully described elsewhere [16]. In addition, we followed the GUIDED and TIDieR recommendations [13] (see Supporting Information SI: Files 1 and 2).

**Table 1:** Intervention mapping steps applied to the codesign of the ALAPAGE programme.

IM Step		ALAPAGE programme codesign steps
Step 1		
	Need assessment	2016–2017 diagnostic study, including:
	PROCEDE model	– A review of international literature on (1) existing programmes in diet and physical activity, (2) barriers and facilitators of seniors' participation in diet and physical activity health programmes
		– The state of the field actions relating to diet and physical activity for older people in France and more specifically in southeastern France
		– Qualitative investigations with professionals involved in field actions and with seniors.
Step 2		
	Identification of the most important determinants	First codesign meeting with dieticians
	Performance objectives	Performance objectives and change objectives decided by the research team
	Matrix of change objectives	Second codesign meeting with dieticians and an expert in physical activity: approval of objectives
Step 3		
	Review programme ideas	Description of the existing workshops by operating partners during the diagnostic study
	Identify theoretical rationale	Choice of ALAPAGE guidelines thanks to the second codesign meeting with dieticians and reviews of literature
	Identify theoretical methods	The expertise of an expert in health psychologyReview of the literature on behaviour change models used in the literature on diet and physical activity among older peopleDiscussion with dieticians and qualified adapted physical activity professionals about its relevance during a third and fourth codesign meeting
	Selection with stakeholders	Identification of behavioural change techniques using a review of the literature
	Identify practical strategies	Identification of practical strategies during a third codesign meeting
Step 4		
	Produce scopes, sequences, and themes	First sequence with operating partners during the diagnostic study
	Choose channels through which to inform about programme topics	Codesign of sequence with dieticians and qualified adapted physical activity professionals during the third codesign meeting
	Creation of documents and protocols	Creation of protocols by the study team and approval of dieticians and qualified adapted physical activity professionals
		Creation of tools
		Pilot study
		Adaptation of tools through the pilot study during a fifth codesign meeting

Abbreviation: IM, intervention mapping.

The first step in intervention mapping (IM) consists of conducting a needs assessment to define the programme outcomes [15].

We carried out a diagnostic study from 2016 to 2020 [14], to (1) identify barriers and facilitators related to seniors' dietary and PA determinants and (2) assess the strengths and limitations of existing interventions.

First, we conducted:

- An exploratory review of international literature on barriers and facilitators of seniors' participation in health promotion programmes. The specific aim of this initial bibliographical search was to shed light on the psychosocial processes underlying the decision to participate – or not – in such preventive actions. It drew on resources from multiple disciplines (social psychology, clinical psychology, sociology, public health, epidemiology) and various databases.
- Three types of interviews with seniors to explore determinants of participation in dietary and/or PA group interventions, as well as behavioural determinants of healthy eating and PA:
  ◦ Six focus groups of 6–15 seniors (*n* = 62) to gather motivations and facilitators related to participation and determinants of healthy eating and PA.
  ◦ Two unstructured interviews with seniors (*n* = 2) who had never participated in any dietary and/or PA group workshop, as a pre‐test survey on barriers to participation.
  ◦ Six semi‐structured interviews with seniors who had never participated in diet and/or PA group workshops, focusing on barriers to participation.
- To identify themes, all interviews underwent a vertical (subjective) analysis to classify relevant elements into categories. In a second phase, frequency analysis was performed to identify the most frequently mentioned themes. Finally, a horizontal (cross‐sectional) analysis established the frequency of each category across interviews. Interview guides are available in Supporting Information S3: File 3. Participants were recruited through partner organisations and selected based on their training and experience with PA/nutrition programmes.
- One codesign meeting with four dieticians who led group workshops, allowing them to share their knowledge and perspectives on the impacts and behavioural change determinants targeted by existing dietary and/or PA group workshops.
  Second, we conducted
- An exploratory review of international literature on dietary and/or PA health promotion programmes targeting seniors. This bibliographic search was performed using the MEDLINE database (via PubMed) for the period 2005 to June 2016, with the following keywords: *diet, physical activity, nutrition/intervention, programme, counselling, education/senior, elder, ageing, seniors*. Studies conducted in developing countries or involving populations with specific pathologies were excluded.
- A state‐of‐the‐art review of dietary and/or PA group interventions for seniors in France, particularly in southeastern France. This review was conducted by examining three databases of existing field actions for seniors and by applying the ‘snowball’ method, which helped identify organisations involved in workshops [17, 18, 19].
- Qualitative investigations with organisations involved in existing diet and/or PA group workshops, based on the previous state‐of‐the‐art review:
- Eighteen organisations were contacted (12 local and 6 regional):
  ◦ Local: *Le Bon Temps* and *Ma Vie* associations (Gard), CCAS of Marseille, Nice, Toulon, Bouc‐Bel‐Air, La Ciotat, social centres La Rougière and Malpassé (Marseille), Contrat Local de Santé du Haut‐Allier, municipality of Nice, Pôle Infos Seniors Marseille Nord (*Géront'O Nord* association).
  ◦ Regional: ASEPT Paca, Caisse d'Assurance Retraite et de la Santé au Travail (Carsat) Sud‐Est, Irips (Centre de prévention Bien vieillir Agirc‐Arrco Paca), Mutualité Française Paca, Mutualité Sociale Agricole, Pôle Services à la Personne.
    The objectives were:
  ◦ To gather their opinions on existing dietary and/or PA group health interventions.
  ◦ To identify potential operational partners based on geographical location, existing interventions offered by the organisation, and motivation expressed during the exchange.
- One working group met four times with professionals from organisations willing to participate in the project in southeastern France, along with a PA professional, to describe existing diet and/or PA group workshops in detail and discuss possibilities for harmonisation.

The second step of IM involves defining performance objectives (i.e. how participants are expected to be immediately affected by the programme) and change objectives (i.e. the programme's immediate effects on the determinants of the health problem). Performance objectives combined with the most important determinants highlighted in step 1 lead to a matrix of change objectives [15]. In step 2 as applied to the ALAPAGE programme, the behavioural change determinants related to healthy diet and PA were identified based on the needs assessment conducted in step 1. Performance and change objectives were then defined on the basis of these determinants. Four dietitians and one expert in PA for older people approved both types of objectives during a second co‐design meeting.

The third step of IM involves reviewing programme ideas with the targeted population and identifying theoretical rationale and behaviour change techniques [15]. Theoretical methods are selected throught a working group involving stakeholders and appropriate practical strategies for applying these methods are subsequently defined [15]. In step 3 of the co‐design of the ALAPAGE programme, a theoretical model of behaviour was chosen on the basis of the expertise of health psychology specialist and the behaviour change models used in the literature. Discussions on its relevance occurred during the third and fourth codesign meetings with four dieticians and three pro‐APA. Behaviour change techniques were identified using Michie's taxonomy [12]. Practical strategies were defined with the four dietitians and three Pro‐APA during the third codesign meeting.

The fourth step of IM advises producing a plan that outlines the scopes, sequences and themes of the programme with the help of the targeted population and operating organisations [15]. Then, channels to inform about programme topics must be decided, and documents and protocols must be created and designed with the help of a creative consultant [15]. For the step 4 applied to the codesign of the ALAPAGE programme, a rough sketch was first decided with the operating partners during the diagnostic study and the third codesign meeting with the four dieticians and three qualified adapted PA professionals. Protocols were written by the research team and approved by dieticians and qualified adapted PA professionals. A pilot study was launched in September 2021 in which 21 seniors supervised by two dieticians and two pro‐APA tested protocols, sequences and materials. A fifth codesign meeting with two dieticians and pro‐APA was organised after the pilot study to identify practical changes that may improve the relevance and applicability of the programme.

## 3 Results

### 3.1 Step 1: Identified Programme Outcomes Based on the Behavioural Change Determinants

#### 3.1.1 Strength and Limits of Existing Health‐Promoting Programmes on Dietary/Pa

The review resulted in the identification of 132 studies (Supporting Information S4: File 4), 50% were conducted in the United States and 20% in Europe. The majority of these studies (56%) focused on PA only, 23% on diet only, and 21% on both. The studies were of various types: impact studies, intervention protocols, analyses of obstacles and levers, and literature reviews. The studies included a total of 44,630 seniors, with sample sizes ranging from 15 to 13,801 participants. Only 32 studies focused on seniors with some form of disability or limited resource [20, 21, 22, 23, 24, 25, 26, 27, 28, 29, 30, 31, 32, 33, 34, 35, 36, 37, 38, 39, 40, 41, 42, 43, 44, 45, 46, 47, 48, 49]. In most of these studies, health programmes were built based on psychosocial theories of behavioural change. The most often cited theories were Bandura's social cognitive theory [22, 25, 26, 27, 36, 40, 47, 50, 51, 52, 53, 54, 55, 56, 57, 58, 59] and Prochaska's transtheoretical stages of change theory [27, 60, 61, 62, 63]. Social Cognitive Theory posits that humans possess several capabilities, including symbolic and forethought capacities; vicarious capability (or modelling), self‐regulatory capacity, and self‐reflective capacity [64]. Prochaska's Transtheoretical Model identifies five stages of change: precontemplation, contemplation, preparation, action, and maintenance [65]. Interventions most often consisted of physical exercise and/or dietary advice, either through the provision of a group workshop [21, 23, 24, 26, 30, 31, 32, 34, 35, 36, 37, 40, 43, 44, 46, 47, 48, 50, 51, 53, 57, 58, 59, 61, 63, 66, 67, 68, 69, 70, 71, 72, 73, 74, 75, 76, 77, 78, 79, 80, 81, 82, 83, 84, 85, 86], by delivering information by phone [20, 38, 56, 62, 87, 88] or by using digital material [52, 61, 89, 90, 91, 92, 93, 94, 95, 96, 97, 98].

In France, field actions aimed at promoting PA and/or a healthy diet among seniors are common, but only some of them have been developed based on theoretical models (i.e. planned behaviour theory) [99]. Most of them are evaluated using a limited number of indicators of process evaluation (e.g. the number of participants or satisfaction surveys) by organisations, but very few have undergone rigorous impact evaluation [99].

In southeastern France, 45 dietary and/or PA field actions were identified and described by our team [14]: 5 were focused on diet only, 13 on PA only, and 27 on both PA and diet. They were mostly addressed to the whole population of seniors, and they did not include caregivers or family (31/45). The actions generally consisted of group workshops(32/45) and lectures (13/45) [14]. The description of these field actions helped us identify key operating organisations and actors.

A total of 18 organisations were identified as potential partners for the codesign of the ALAPAGE programme and contacted, leading to the establishment of three partnerships: two regional operating organisations (Carsat Sud‐Est, Mutualité Française Sud) and one local operating organisation (Géront'ONord). Discussions with professionals from operating organisations allowed us to identify four main limits to existing workshops on diet and/or PA: (i) a lack of effectiveness assessment; (ii) a heterogeneous and unstandardised form, limiting the possibility of evaluating their effectiveness; (iii) a lack of attractiveness compared to other kinds of workshops dedicated to older people, for instance, about memory or sleep; and (iv) difficulties reaching socially isolated people. Indeed, regular participants in existing workshops are often proactive adults who are already aware of the importance of having a healthy, diverse diet and regular PA.

#### 3.1.2 Seniors Dietary and PA Determinants

Evidence from the literature indicates that various psychological factors may discourage seniors from participating in health‐promoting programmes focused on diet and PA.

First, lack of confidence and low belief in one's physical abilities – such as fear of falling – can deter participation [100], whereas those with a strong sense of self‐efficacy are more likely to engage [101]. Second, some ‘seniors remain comparatively optimistic about their health’ [102] (i.e. they believe health problems will only affect others), which can reduce motivation to join health programmes. Third, seniors often have a limited future perspective [103] (i.e. they focus on the present), while participation in a programme requires an extended future perspective. Fourth, although older adults are generally aware of nutrition recommendations, they perceive them as difficult to implement [104]. Using Ajzen's theoretical model, nutritional recommendations may foster a more favourable attitude but fail to address perceived behavioural control – the parameter most strongly correlated with behaviour after intention [105]. Physical activity recommendations are rarely known, and seniors often believe they already exercise enough [106]. Fifth, age‐related discrimination can lead some seniors to avoid identifying themselves as ‘old’, viewing public health programmes as stigmatising [101]. Agreeing to participate in a dedicated workshop for seniors, especially on fall prevention, may imply relinquishing an identity of independence, as falls and fall‐prevention programmes are associated with dependency [101]. Sixth, poor health, advanced age, fatigue, fear of tiredness or pain, and lack of family support have also been described as barriers to participation [100]. Finally, the location of the PA workshop can be critical. For example, programmes held in hospitals may be poorly perceived because hospitals are associated with illness and death [100, 101].

The 62 seniors interviewed were aged between 65 and 80 years old and were mostly women. Gaining knowledge and verifying information from the media were the first motives cited by the interviewees. They also mentioned the desire to adopt a more balanced diet while still enjoying eating. Maintaining social links and meeting new people were also important motives. Some interviewees said that knowing that there will be fun activities in a pleasant place motivated them. Moreover, the place has to be near their home because seniors talked about the ‘cost of moving’ (including physical, psychological, and not particularly, or not only, financial costs). Lastly, interviewees considered it important that the dietary and/or PA group workshop is led by a skilled professional who is able to pass on knowledge to promote group cohesion and facilitate good relationships between participants. The interviews also helped identify seniors' perceptions of the workshop and of the behavioural changes that can occur after the workshop. Interviewees talked about their difficulties in maintaining a healthy diet but did not talk much about PA. Concerning PA, they reported that the workshops helped them increase their walking. For them, their loneliness due to ageing and life events (e.g. retirement) make it more difficult to maintain a healthy diet. Moreover, they said that changing their dietary habits is difficult because they are part of their identity. Finally, the interviews confirmed that older people knew dietary recommendations, but they saw them as difficult to put into action, as explained previously in the literature.

Before the first codesign meeting, four dieticians (women, aged between 30 and 60 years old) were asked to list the behavioural change determinants of adoption of a healthy diet for the participants of the existing field actions. The most important determinants were then chosen if they were unanimously agreed upon.

#### 3.1.3 Behavioural Determinants of Healthy Eating and PA Targeted by the Alapage Programme

Based on step 1 relieving both of literature and opinion from stakeholders, 12 determinants of both healthy eating and PA in seniors were selected as the most important to consider (e.g. being motivated) (Table 2). Moreover, three determinants specific to PA (e.g. physical habits) and 3 determinants specific to healthy eating (e.g. physical habits) were considered.

**Table 2:** Main determinants of diet and physical activity for seniors to be targeted during the ALAPAGE programme, identified during step 1.

Main determinants identified in Step 1		Source		
Common determinants		Seniors	Professional	Literature
	Receiving opposing information	x		
	Being motivated	x		
	Rejecting recommendations	x		x
	Knowledge	x		
	Beliefs		x	
	Thinking that recommendations are already Applied			x
	Representation			x
	Receiving support	x		x
	Being willing to improve one's health	x		x
	Having self‐esteem			x
	Being stressed			x
	Having experience		x	
Diet determinants only				
	Food habits	x		
	Fearing being deprived of food pleasure		x	
	Food costs		x	
Physical activity determinants only				
	Physical activity costs		x	
	Physical habits		x	
	Fearing of tireness or pain	x		x

#### 3.1.4 ALAPAGE Programme Outcomes

Data collected as part of step 1 allows us to identify the two ALAPAGE programme outcomes:

A higher dietary diversity, usually defined as the number of different foods or food groups consumed over a given reference period, may contribute to postponing age‐related chronic diseases [107, 108]. Indeed, studies exploring links between dietary diversity and health reported that greater dietary diversity was associated with a reduced risk of all‐cause mortality [109] and can help to maintain good physical function [108]. Moreover, it seemed to our expert group that there is a ‘pedagogical’ interest in using dietary diversity as a guiding principle, as the message is relatively simple to explain/understand and ‘positive’. This idea was approved by dieticians during the third codesign meeting. The ALAPAGE programme drew on a previously published dietary diversity intervention: a programme called Sumida Take Ten! aims to promote 10 min of PA per day and greater dietary diversity by conference and by monitoring the consumption of 10 food groups [78]. This programme, which had been tested over 92 seniors living at home, showed a significant increase in dietary diversity.

Preventing falls and maintaining functional physical abilities have been effectively improved by programmes that combine balance and muscle‐strengthening exercises. Among these, the innovative Australian LiFE (Lifestyle integrated Functional Exercise) [110] specifically incorporated PA into everyday tasks and routines (e.g. standing on one foot while cooking). In people over 70 years of age with a history of falls, LIFE was shown to be effective in reducing the risk of falling and improving balance, certain muscle strength parameters, overall PA level, and participation in daily living activities and social activities at 6 and 12 months. The pro‐APA approved the idea of increasing physical fitness by increasing PA embedded in everyday movements during the fourth codesign meeting. Traditional supervised exercises (number of repetitions, timed exertions, etc.) were added to LIFE during the codesign meeting where the PA professional put forward that some seniors would not adhere to LIFE.

### 3.2 Step 2: Matrix of Change Objectives

#### 3.2.1 Performance Objectives

Three performance objectives have been defined and applied to the diet and PA outcome:

1. Understanding the importance of high dietary diversity/good physical fitness.
2. Knowing how to adopt high dietary diversity/how to increase physical fitness.
3. Increasing the diversity of one's diet/one's level of physical fitness.

#### 3.2.2 Matrix of the ALAPAGE Change Objectives

A matrix of change objectives was constructed for both programme outcome (Table 3 for dietary diversity and Table 4 for physical fitness). A total of 15 change objectives have been identified for increasing dietary diversity, and 16 others have been identified for increasing physical fitness (e.g. understanding what daily PA is).

**Table 3:** Matrix of dietary diversity change objectives.

	Determinants								
Performance objectives	Receiving opposing information	Fearing being deprived of food pleasure	Rejecting recommendations	Knowledge	Beliefs	Thinking that recommendations are already applied	Representation	Receiving support	
P1: understanding the importance of a high dietary diversity	1. Questioning habits, beliefs, and representations			2. Understanding that becoming older can lead to illness and physiological change	3. Understanding that high dietary diversity is compatible with a sustainable diet	4. Understanding what high dietary diversity means	1. Questioning habits, beliefs and representations	5. Believing that the general practitioner thinks it is important to have a high dietary diversity	6. Believing that other participants and relatives think that it is important to have a high dietary diversity
P2: knowing how to adopt high dietary diversity		7. Identifying one's barrier	7. Identifying one's barrier	8. Knowledge of local patient support groups					
P3: increasing the diversity of one's diet									

	Determinants						
Performance objectives	Food costs	Willing to improve one's health	Having self‐esteem	Being stressed	Being motivated	Food habits	Having experience
P1: understanding the importance of high dietary diversity				9. Being reassured about diet			
P2: knowing how to adopt high dietary diversity							15. Knowledge of how to choose food according to its nutritional value
P3: increasing the diversity of one's diet	10. Knowing that high dietary diversity is possible at a low cost	11. Setting a personalised objective	12. Achieving a personalised objective	13. Feeling capable of making changes	14. Motivating oneself to increase dietary diversity	12. Achieving a personalised objective	11. Setting a personalised objective

**Table 4:** Matrix of change objectives for physical activity.

	Determinants							
Performance objectives	Fearing having to do a strict physical activity	Rejecting recommendations	Knowledge	Beliefs	Thinking that recommendations are already applied	Representation	Receiving support	
P1: Understanding the importance of good physical fitness			1. Understanding that becoming older can lead to illness and physiological change	2. Making the distinction between sport and physical activity	3. Understanding what daily physical activity is	4. Questioning habits, beliefs, and representations	5. Believing that other participants and relatives think it is important to practice daily physical activity	6. Believing that the general practitioner thinks it is important to practice daily physical activity
P2: Knowing how to increase physical fitness	7. Identifying one's barrier	7. Identifying one's barrier	8. Knowledge of local patient support groups					
P3: Increasing one's level of physical fitness								

	Determinants							
Performance objectives	Being motivated	Being stressed	Having self‐esteem	Receiving opposing information	Willing to improve one's health	Cost of physical activity	Having experience	Physical habits
P1: understanding the importance of daily physical activity		9. Doing a daily physical activity safely		4. Questioning habits, beliefs, and representations				
P2: Knowing how to construct physical activity and how to adapt to each one		10. Knowing that daily physical activity needs no investment				11. Knowing that daily physical activity needs no monetary investment	12. Choosing one's physical activity depending on perceived pleasure, one's habits and the level of ease	
P3:Increasing one's level of physical activity	13. Motivating oneself to increase one daily physical activity	14. Feeling capable out of making changes	15. Achieving a personalised objective		16. Setting a personalised objective		15. Setting a personalised objective	16. Achieving a personalised objective

### 3.3 Step 3: Theoretical Model, Behavioural Change Techniques, and Practical Strategies

#### 3.3.1 ALAPAGE Theoretical Model and Declination From Determinants to Behaviour Change Techniques

Advised by an expert in public health psychology, we selected Ajzen's theory of planned behaviour, which predicts that planned behaviours are determined by intention. This intention is mostly influenced by attitude, social norms, and perception of control over the behaviour [111].

However, if a limitation of this theory lies in the fact that there is an ‘intention‐behaviours gap’ due to goal conflicts [112], setting clear goals and planning action can be a good way to reduce this gap because ‘the extent to which people have planned an action is predictive of achieving a behaviour‘ [112]. The expert in public health psychology advised to use the Ajzen's theory of planned behaviour enhanced by the Goal Setting Theory.

We first associated the change objectives with the parameters of the theoretical model on which they had the most influence. For example, the change objective ‘understanding that high dietary diversity is compatible with a sustainable diet’ is associated with perceived control (Table 5 for dietary diversity and Table 6 for physical fitness). Second, the research team has identified 8 behavioural change techniques to increase dietary diversity and 12 other behavioural change techniques to increase physical fitness. For example, for the change objective ‘understanding that high dietary diversity is compatible with a sustainable diet’, the behavioural change technique ‘information about social and environmental consequences’ was identified (Tables 5 and 6).

**Table 5:** Theoretical model to increase dietary diversity.

ALAPAGE theory parameters	Change objectives	Behavioural change techniques	Practical strategies	Pedagogical tools
Attitude	Understanding that becoming older can lead to illness and physiological change	Information about antecedents	Group talk	
	Knowing what high dietary diversity is	Instruction on how to perform a behaviour	Game: the 11 ALAPAGE diversity families	Food cards
	Questioning habits, beliefs, and representations	Reattribution	Group talk on ideas received	Food cards
			Quiz	Quiz paper for dieticians'
			Game: the 11 ALAPAGE diversity families	food cards
Perceived norms	Believing that the general practitioner thinks it important to have high dietary diversity	Social support	Speaking with the general practitioner	Letter for General Practitioner
	Believing that other participants think it important to have high dietary diversity		Group talk	
Perceived control	Knowledge of local patient support groups	Information about consequences to health	Group talk and provision of information on local patient support groups	Information on local patient support groups
	Identifying one's barrier	Information about antecedents	Quiz	Quiz paper for dieticians
			Group talk on ideas received	
		Action planning	Creating a plan of action once personalised goals are set	Goal setting and planning
	Being reassured about diet	Reattribution	Group talk on ideas received	
			Quiz	
			Game: the 11 ALAPAGE diversity families	
	Knowing how to choose food according to nutritional value	Instructions on how to perform a behaviour	Game: ‘SAIN‐LIM’	SAIN‐LIM board
				Food cards
	Knowing that eating a highly diverse diet is possible at low cost	Information about antecedents	Tasting different brands of a same product (cheap, average, expensive)	
	Understanding that high dietary diversity is compatible with a sustainable diet	Information about social and environmental consequences	Group talk from photos	Photo‐language
				Recipes
	Motivating oneself to increase dietary diversity	Adding objects to the environment	Being given a magnet to put on the fridge to help to remember the goals set	Diversity magnet
	Feeling capable of making changes	Graded tasks	When setting goals, dieticians will insist on setting a small goal first	
Goal setting theory	Setting a personalised objective	Goal setting	Setting a personalised goal using a self‐analysis of dietary diversity	The 11 ALAPAGE families sorting quiz
		Self‐monitoring	24‐h dietary recall	Goal setting and planning
		Reviewing behaviour goal(s)	Setting a personalised goal using a self‐analysis of dietary diversity at the end of the ALAPAGE programme	Goal setting and planning
	Achieving a personalised objective	Feedback on behaviour	Group talk about goals at the beginning of each session	
		Action planning	Starting with personalised goals, the participant will plan their goals during the session	Goal setting and planning
			Group talk to organise activities after the ALAPAGE programme ends	

**Table 6:** Theoretical model to increase daily physical activity.

ALAPAGE theory parameters	Change objectives	Behavioural change techniques	Practical strategies	Pedagogical tools
Attitude	Understanding that becoming older can lead to illness and physiological change	Information about antecedents	Senior fitness test	
	Understanding what daily physical activity is	Instructions on how to perform a behaviour	Level 1 story The daily bracket	Golden rules in the notebookOnline daily bracket
	Differentiating between sport and physical activity	Instructions on how to perform a behaviour	PA quiz	
	Performing daily physical activity safely	ReattributionInstructions on how to perform a behaviour	Group talk Level 1 story The daily bracket	Golden rules in the notebookOnline daily bracket
	Questioning habits, beliefs, and representations	Reattribution	PA quiz	
Perceived norms	Believing that other participants and relatives think it important to practice a daily physical activity	Social support	Level 2 story to do with relatives	Notebook
	Believing that the general practitioner thinks it important to practice a daily physical activity	Social support	Speaking with the general practitioner	General practitioner letter
Perceived control	Identifying one's barrier	Information about antecedents	Senior fitness test Group talk about results	Results and norms
	Choosing one's physical activity according to perceived pleasure, habits and level of ease	Instructions on how to perform a behaviour	Level 1 story The daily bracket	Notebooks
		Demonstration of the behaviour	Level 1 story The daily bracket	Notebooks
		Information about antecedents	Senior fitness test Group talk about results	Information on results and norms
	Knowledge of local patient support groups	Practical social support	Group talk	Local patient support group information
	Knowing that daily physical activity needs no monetary investment	Information about antecedents	Level 1 story	Notebooks
	Feeling capable of making changes	Graded tasks	Identification of 4 levels of difficulty	Information on results and normsLevel exercises in notebooks
		Self‐monitoring of behaviour	Home exercises	PedometersDaily routine chart
		Focus on past success	Writing down home exercises accomplished	Daily routine chart
	Motivating oneself to increase one daily physical activity	Adding objects to the environment		Pedometers Notebooks Daily routine chart Online daily bracket
		Feedback on behaviour	Group talk	Daily routine chart
Goal setting theory	Setting a personalised objective	Goal setting	Group talk about home exercises Choice of 1 of 3 notebooks	Stamina notebook Balance notebook Strength and suppleness notebook
		Reviewing behaviour goal(s)	Group talk after home exercises	
	Achieving a personalised objective	Action planning	Group talk about the daily routine chart	
		Habit formation	Daily bracket and notebook exercises to do at home	Notebooks

Abbreviation: PA, physical activity.

#### 3.3.2 ALAPAGE Practical Strategies

The choice of practical strategies has been guided by reviews of the literature and stakeholders' opinions reported during the diagnostic study [113].

Based on the result of step 1, we applied the following key principles to codesign the ALAPAGE practical strategies: to ensure that participants take pleasure in the activity to work on stereotypes, to perform group activities, to focus on positive reinforcement, to take into account individuals, to go step by step, and to take small steps.

Different practical strategies have been used to target the change objectives. Concerning the diet component, group talks have been chosen to reinforce social links. Special games have been either created (e.g. the 11 ALAPAGE diversity families and the sustainable photolanguage) or reused (such as the SAIN‐LIM game from the Opticourses study [114]) to teach nutritional concepts and to replace the usual lectures. Moreover, to help participants self‐analyse their diet, set goals, and plan action, special activities have been developed. Participants complete 24‐h dietary recall and food frequency questionnaires and analyse their consumption of the 11 food diversity families in ALAPAGE. They can then choose to increase or decrease the consumption of one food family and consider how they will achieve their goals. At the end of the programme, they again fill out 24‐h dietary recall and food frequency questionnaires, and analyse them out to see how they changed their diet and to prepare their goals for the post‐ALAPAGE programme period (Table 7).

**Table 7:** ALAPAGE detailed programme.

Session	Professional	Total duration	Activity	Duration (min)	Home activities
S0	Both	2h30	Introduction of the dietician and APA pro	5	
			Introduction in pairs and round table discussion	30	
			Introduction to the programme and the research project	50	
			Distribution/explanation/filling in an example of a mock daily routine chart	10	
			24‐h recall and food frequency questionnaire	20	
			Debate on physical activity and diet	35	
S1	Dietician	2h30	Submission of weeknote 1, distribution of daily routine chart 2	30	Use of pedometer at home
			24‐h recall and questionnaire	30	
			Preconceived ideas about food	30	
			The 11 ALAPAGE diversity families	30	
			Sort your 24‐h recall into ALAPAGE diversity families	30	
S2	APA pro	2h30	Welcome and submission of daily routine chart 1, distribution of daily routine chart 2	10	
			Fitness assessment: SFT battery and Unipodal Balance Test and questionnaires	70	
			Chat together about the results	10	
			Presentation of the home activities over two sessions	25	
			Level 1 story ‘My typical day in action’	20	
			Learn the daily bracket	15	
S3	Dietician	2h30	Group talk about previous session, submission of daily routine chart 2, distribution of daily routine chart 3	15	Home exercises and use of pedometer at home
			SAIN‐LIM game	60	
			Disease prevention	30	
			Planning for more dietary diversity	45	
S4	Dietician	2h30	Group talk about previous session, submission of the daily routine chart 3, distribution of daily routine chart 4	30	
			Sustainable diet	65	
			Group talk about post‐ALAPAGE programme activities	25	
			Planning for more dietary diversity	30	
S5	Dietician	2h30	Group talk about previous session, submission of daily routine chart 4, distribution of daily routine chart 5	15	
			‘Pleasure and budget’ tasting	55	
			Group talk about post‐ALAPAGE programme activities	25	
			24‐h recall	15	
			Questionnaire	20	
			Sort your 24‐h recall into ALAPAGE diversity families and plan for more dietary diversity	20	
S6	APA pro	2h30	24‐h recall	20	
			Group talk about home exercises between sessions	10	
			Unipodal Balance Test and questionnaires	30	
			Comparison with the test results obtained in S2	25	
			Level 2 story ‘My typical day in action’	45	
			Group talk about post‐ALAPAGE programme activities	20	
**Activities organised by older people to keep making progress**					
S7	APA pro	2h30	24‐h recall and food frequency questionnaire	30	
			Fitness assessment: SFT battery, Unipodal Balance Test, questionnaires, and submission of daily routine chart	70	
			Group talk about results and the last three months	20	
			Results sharing over a snack	30	

Abbreviation: APA pro, adapted physical activity professionals.

Regarding the PA component, participants first answer a quiz to question their beliefs about PA and are given a pedometer to help them evaluate and follow their daily steps. They then learn about their initial physical fitness using the Senior Fitness Test [115] and unipodal balance test. A PA professional teaches them two types of exercises: Physical exercises are inspired by the LIFE [110], which focuses solely on balance and muscle strengthening, and has also been expanded by the ALAPAGE research group to include endurance exercises. The participants choose a level of difficulty and a physical quality that they prioritised to improve (strength‐flexibility, balance, or endurance) and work on using their test results, based on a booklet with ideas for integrating their PA into everyday activities. To respond to seniors who would prefer traditional supervised exercises, as advised by pro‐APA, a 10‐min traditional supervised exercise called the ‘daily bracket’ is proposed and is available both in the booklet and online. It consists of following a ‘take a 10‐min break for 10 exercises’ targeting upper and lower limb muscle strengthening, aerobic endurance and flexibility.

Between each session, whether it is a dietary or PA session, they have to fulfil a daily routine chart about how many steps they walked, which daily exercises they have done and how many daily brackets they have completed. At the end of the ALAPAGE programme, they once again take the Senior Fitness test and unipodal tests to see how much they have improved and prepare activities to do with the whole group or online to stay active (Table 7).

### 3.4 **Step 4:** Co‐Design for Planning the ALAPAGE Programme With the Scopes, Sequences, and Themes

To be consistent with existing workshops, the ALAPAGE programme consists of eight group sessions lasting 2 h 30 min, which are delivered in community centres to seniors by dieticians and PA professionals trained in the ALAPAGE programme [16]. A total of 15 seniors are recruited by community centres or by an innovative active recruitment strategy to improve the participation of hard‐to‐reach seniors [116]. The scope and sequence of the sessions are described in Table 7. The activity protocols are available on request, and the activity and materials are described in Supporting Information S2: File 2. The session, called the ‘introduction session’, allows us to present the programme and to enhance group cohesion. Compared to existing field actions, the ALAPAGE programme once again addresses the following themes: dietary diversity, the nutritional profiles of foods, budget (healthy eating on a budget), sustainable diet and, regarding PA, balance, flexibility, strength and aerobic exercises incorporated into everyday tasks, and a 10‐min routine exercise [16]. A total of 11 pedagogic tools were used for the diet component of the programme, and 8 pedagogic tools were used for the PA component, such as three notebooks with exercises to perform at home depending on the chosen physical quality (Tables 5 and 6).

A logo and a graphic chart have been created (see Supporting Information S5: File 5). A creative consultant has designed the PA notebooks. The programme was tested during a pilot study. The pilot involved testing the programme with two groups of 10 seniors through final workshops (2.5‐h sessions led by a dietician or an pro‐APA as described in Table 7) organised in two different local organisations accustomed hosting routine prevention workshops. Stakeholders suggested only minor changes: to extend the group session from 2 h to 2 h 30 min and to redesign the characters in the notebook because women with grey hair and bald men seem too old from the participants' point of view.

## 4 Discussion

This article describes how the codesign and the theoretical rationale of the ALAPAGE programme have been constructed based on the IM. Based on the target population, existing programmes in the PACA region, and insights from professionals, we developed a programme tailored to this specific context.

Step 1 of IM highlighted the major behavioural change determinants of seniors' diet and PA and participation that will be targeted in the ALAPAGE programme. Additionally, in this step, the main outcomes have been defined: to improve dietary diversity and to increase physical fitness. Step 2 led to defining the 31 ALAPAGE change objectives (15 and 16 for the diet and PA components, respectively). In step 3, the theoretical rationale, combining Ajzen's theory of planned behaviour and Goal Setting Theory, 25 behavioural change techniques practical strategies were decided. Lastly, during step 4, 19 pedagogical tools were created or used (11 for increasing dietary diversity and 8 for increasing physical fitness), and the sequence and scope of the 7 ALAPAGE sessions were defined.

Using the IM to develop a health‐promoting intervention helped to report it more precisely, as recommended [13], and may help face the challenges of scaling up or adapting the programme to other contexts. Finally, we found that the IM process is very adapted to the reality of real‐world circumstances.

To our knowledge at this time, only two studies about dietary and/or PA health promotion programmes in seniors described how they codesigned their intervention [97, 117].

Few studies that aim to promote senior health without focusing on one special illness and that combined PA and nutritional advice described their theoretical rationale [118]. During a recent systematic review, which included studies from 2010 to 2020, only 15 interventions (out of 31) targeted education or participation within the session for community‐dwelling seniors and did not target specific diseases. Only 9 referred to the theoretical model used. The social cognitive model was the most widely used (3/15), followed by the transtheoretical model (2/15). No one has used the theory of planned behaviour [118]. After being advised by a psychologist and according to the determinants co‐identified with dieticians, we chose to use the theory of planned behaviour. It is not the most widely used model. However, it is the option that best fits our intervention, as it aligns closely with the behavioural determinants and objectives identified in Step 1.

Two studies on seniors, one about the consumption of dairy products and one on vegetables consumption, showed that 40% of the variability of consumption is explained by intention [119, 120]. Moreover, a review reported studies showing that the theory of planned behaviour explains 45%, 55% and 72% of the variance in PA intention in older people [121]. Other models, such as COM‐B [122], are more contemporary and better able to account for participants' environment, which is not the case with Azjen's model They could be beneficial for designing similar interventions.

To adapt the programme to another context, it would be necessary to verify whether the behavioural determinants of healthy eating and PA (Step 1) are similar to those identified in this study and to adjust the behaviour change techniques and activities (Steps 3 and 4) accordingly.

For greater reproducibility, it is essential to apply a structured intervention design method, use a theoretical framework, and draw on both evidence from the literature and field expertise.

This study has several limitations. First, the process developed was inspired by the IM but deviated from it in some aspects. No PRECEDE model was created for the need assessment, and codesign with seniors was limited to step 1. In addition, while the study was co‐designed with most stakeholders involved in existing workshops, community centres were not included because of their heterogeneity. Finally, all tools were developed in French, as required for the ALAPAGE research context. However, translation into other languages may be considered in the future to allow adaptation to local communities (e.g., some older adults in south‐eastern France read and write only in Arabic).

The strength of this study is the involvement of various stakeholders from the existing workshops, and the theoretical rationale being adequately described. Moreover, as advised by the IM, the ALAPAGE development has considered the literature reviews on seniors' diet or physical health programmes. Finally, it is the first dietary and PA programme for French older people to have its codesign construction described. These characteristics should lead to an effective programme that can be implemented in the field on a routine basis [13].

## 5 Conclusion

Based on the Intervention Mapping approach, the co‐designed ALAPAGE programme employs behaviour change techniques to address determinants of diet and physical activity among seniors. Furthermore, the explicit description of this development process should enhance its transferability and dissemination. The impact assessment is currently underway.

## Author Contributions

**Anne‐Fleur Jacquemot:** conceptualisation, writing – original draft, methodology; writing – review and editing, investigation. **Aurélie Bocquier:** conceptualisation, writing – original draft, funding acquisition, methodology, investigation. **Agnès Vinet:** conceptualisation, investigation, writing – original draft, methodology, funding acquisition. **Christophe Dubois:** conceptualisation, writing – original draft, funding acquisition, methodology, investigation. **Nicole Darmon:** conceptualisation, investigation, writing – original draft, methodology, funding acquisition. **Florence Cousson Gélie:** conceptualisation, writing – original draft, methodology. **Catherine Féart:** writing – original draft. **Pierre Verger:** investigation, writing – original draft. **Sarah Danthony:** conceptualisation, writing – original draft, methodology; writing – review and editing, investigation.

## Ethics Statement

The ALAPAGE study was approved by the French ethics committee ‘Comité de Protection des Personnes TOURS ‑ Région Centre ‑ Ouest 1’ (FDA IRB n° IORG0008143) on 30 March 2021 (n° 2021T2–06) equivalent of an Institutional Review Board with an expedited review. Oral consents of older adults and of professional from organisations were obtained, implied consents of professional (dieticians and adapted physical activity professionals) were obtained, and written consents of professionals from operational partner were obtained. Major changes to this study protocol will be communicated to the ethics committee for approval.

## Consent

The authors have nothing to report.

## Conflicts of Interest

The authors declare no conflicts of interest.

## Supporting information


**Supporting file 1:** The Guided Checklist for the ALAPAGE Multicomponent Programme.

**Supporting file 2:** TIDieR checklist for the ALAPAGE multicomponent intervention.

**Supporting file 3:** Reference list of the review on the existing interventions.

**Supporting file 4:** Interview guide.

**Supporting file 5:** Example of material with a graphic chart: The diversity magnet.

## Data Availability

The protocols of the activity are available on request, and the activity and materials are described in Additional file 2. The details on the diagnostic study can be found online: http://www.orspaca.org/productions/publication/rapport-final-projet-alapage
