# Supplementary material for: Intervention Mapping: A Framework to Co‐Design the ALAPAGE Programme to Simultaneously Improve Dietary Diversity and Physical Fitness Among Older People
Source: Health Expect. 2026 Mar 23;29(2):e70612. doi: 10.1111/hex.70612 (PMC13087432; doi:10.1111/hex.70612)
Supplement: Supplementary file 2 — Supporting file 2: TIDieR checklist for the ALAPAGE multicomponent intervention. [file HEX-29-e70612-s005.pdf]

Additional file 2: TIDieR checklist for the ALAPAGE multicomponent intervention

Item number	Item	Description
BRIEF NAME		
1.	Provide the name or a phrase that describes the intervention.	A combined diet/physical activity intervention to improve dietary diversity and physical fitness among French older people living at home (the ALAPAGE programme)
WHY		
2.	Describe any rationale, theory, or goal of the elements essential to the intervention.	Dietary diversity and daily physical activity can help to postpone the onset of age-related health problems (30,33). Current interventions that promote healthy eating and physical activity among the elderly have limitations (16) and evidence of French interventions' effectiveness is lacking (28).<br><u>Rationale for component 1 "Dietary diversity":</u><br>As explained by ALAPAGE programme stakeholders, field actions on healthy diets seemed to be less appealing because of the difficulty of having a diet close to diet recommendations. Moreover, it seemed to our expert group that there is a "pedagogical" interest in using dietary diversity as a guiding principle, as the message is relatively simple to explain/understand and "positive". Studies exploring links between dietary diversity and health reported that greater dietary diversity was associated with a reduced risk of all-cause mortality (47), and can help to maintain good physical function (30). A previous dietary diversity programme conducted in Japan was effective in increasing dietary diversity (32).<br><u>Rationale for component 2 "Daily physical activity":</u><br>Programmes that combined balance and muscle-strengthening exercises have effectively improved preventing falls and maintaining functional physical abilities. Among these, the innovative Australian programme LiFE (Lifestyle integrated Functional Exercise) (12) specifically proposed exercises that can be carried out by performing daily tasks as double-tasks (e.g. standing on one foot while cooking, bending the knees rather than bending forward to pick up an object on the ground). In people over 70 years of age with a history of falls, this programme was shown to be effective in reducing the risk of falling and improving balance, certain muscle strength parameters, overall physical activity level, and participation in daily living activities and social activities at 6 and 12 months.
WHAT		
3.	Materials: Describe any physical or informational materials used in the intervention, including those	<u>Materials provided for component 1 "Dietary diversity":</u><br>- Group sessions 0,1, 3, 4, and 5 in the ALAPAGE programme:<br>During the session 0, a letter for the general practitioner and information on local patient support groups are given to the participants.<br>To address the concept of dietary diversity, a quiz sheet called a SAIN-LIM board, food cards, photolanguage, recipes, a diversity magnet, the 11 ALAPAGE families sorting paper, and 24-hour dietary recall are used.<br>To help to set a goal, a goal setting and planning sheet is used<br><u>Materials provided for component 2 "Daily physical activity":</u>

	provided to participants or used in intervention delivery or in training of intervention providers. Provide information on where the materials can be accessed (e.g. online appendix, URL).	-Group sessions 0, 2 and 6 in the ALAPAGE programme:<br>To learn about given ideas, the quiz sheet is used<br>To monitor participants' physical activity, a pedometer, a daily routine chart and information on results and norms are given to the participant.<br>Moreover, three different notebooks, with exercises, are provided in sessions 2 and 6.<br>A demonstration of some exercises is available in a YouTube video "la parenthèse quotidienne" https://www.youtube.com/watch?v=lfdz6 Wa6vrg<br>Materials provided for the training of intervention providers:<br>Dieticians are provided with the session protocols, leaflets about dietary diversity, sustainable diet, the ALAPAGE theoretical model and nutritional profile.<br>Physical activity professionals are provided with session protocols, tests, and weights<br>All materials are available on request from the authors
4.	Procedures: Describe each of the procedures, activities, and/or processes used in the intervention, including any enabling or support activities.	Implementation of the ALAPAGE programme:<br>A local community centre responds to a call for applications to participate in the ALAPAGE programme and designates a "project leader", who will attend a web conference about the ALAPAGE programme organised by the research team.<br>The project leader from the local community centre and intervention providers decide on the date of the ALAPAGE programme and inform the research team. Two months before the ALAPAGE programme begins, the research team call the project leader to explain the programme again<br>Participant recruitment is by the staff in local community centres and using an innovative active recruitment strategy that targets hard-to-reach people (39). The research team provided the community centre with all the material needed.<br>Procedures for component 1 "Dietary diversity":<br>- During group sessions 0,1, 3, 4, and 5 in the ALAPAGE programme, the dietician follows the activities protocol.<br>Procedures for component 2 "Daily physical activity":<br>During group sessions 0, 2, and 6 in the ALAPAGE programme, the physical activity professional follows the activities protocol.
WHO PROVIDED		
5.	For each category of intervention provider (e.g. psychologist, nursing assistant), describe their expertise, background and any specific training given.	For both components, physical activity professionals and dieticians have completed two days of training with the research team about how to conduct the research (tests and questionnaires and how to bring the ALAPAGE programme to life.<br>Physical activity professionals have a Masters in adapted physical activity.
HOW		

6.	Describe the modes of delivery (e.g. face-to-face or by some other mechanism, such as internet or telephone) of the intervention and whether it was provided individually or in a group.	The ALAPAGE programme is provided in groups, face-to-face. At home, the participants have some activities to do alone (wear the pedometer, complete the daily routine chart and one 24-recall, as well as completing the exercises in one notebook)
WHERE		
7.	Describe the type(s) of location(s) where the intervention occurred, including any necessary infrastructure or relevant features.	The ALAPAGE programme takes place in the local community centres.
WHEN and HOW MUCH		
8.	Describe the number of times the intervention was delivered and over what period of time including the number of sessions, their schedule, and their duration, intensity or dose.	The ALAPAGE programme comprises 7 weekly group sessions. The participants are meant to complete the ALAPAGE programme only once. Each group session lasts 2 hours 30 minutes.
TAILORING		
9.	If the intervention was planned to be	Using 24-hour recall and the physical activity tests, the participant can choose one objective to help them increase their dietary diversity and one notebook of three to improve one physical quality (stamina, balance, or strength and suppleness). Moreover, they can choose whether they prefer to do the exercises in one notebook or to do the daily bracket

	personalized, titrated or adapted, then describe what, why, when, and how.	
MODIFICATIONS		
10.	If the intervention was modified during the course of the study, describe the changes (what, why, when, and how).	N/A - Adaptations will be assessed as part of the implementation evaluation during the ALAPAGE study (18).
HOW WELL		
11.	Planned: If intervention adherence or fidelity was assessed, describe how and by whom, and if any strategies were used to maintain or improve fidelity, describe them.	Intervention components' dose and fidelity will be assessed as part of the implementation evaluation during the ALAPAGE study (18). They will be assessed using regular activity reports collected on a standardised form, completed after each group session by dieticians or APA professionals. Questionnaires are assessed by the project leader of community centre.
12.	Actual: If intervention adherence or fidelity was assessed, describe the extent to which the intervention was delivered as planned.	N/A - Intervention fidelity will be assessed as part of the implementation evaluation during the ALAPAGE study (Bocquier et al., 2022).
