# Supplementary material for: Intervention Mapping: A Framework to Co‐Design the ALAPAGE Programme to Simultaneously Improve Dietary Diversity and Physical Fitness Among Older People
Source: Health Expect. 2026 Mar 23;29(2):e70612. doi: 10.1111/hex.70612 (PMC13087432; doi:10.1111/hex.70612)
Supplement: Supplementary file 3 — Supporting file 3: Reference list of the review on the existing interventions. [file HEX-29-e70612-s004.rtf]

Reference List

	1. 	Akobundu UO, Netterville L. Meeting the Training Needs of Aging Network Nutrition Program Professionals: Past, Present, and Future. J.Nutr Gerontol.Geriatr. 2015;34:110-23.
Ref ID: 31
Keywords: Aged/Aged,80 and over/Aging/Community Health Services/economics/Delivery of Health Care/trends/Education,Continuing/Food Services/legislation & jurisprudence/organization & administration/Health Services for the Aged/Home Care Services/Humans/Insurance,Health/Long-Term Care/methods/Nutrition Policy/Nutritional Physiological Phenomena/Nutritionists/education/United States
Reprint: Not in File
Abstract: Aging network nutrition professionals must continuously adapt knowledge and skills in order to maintain the provision of high quality, appropriate, and targeted services able to address the evolving demographic, home- and health care-needs of the older Americans of today and tomorrow. This evolution must be supported by ready access to contemporary training and technical assistance. Since the passage of the Older Americans Act in 1972, the Administration on Aging has provided a diverse and contemporary array of supportive program development modalities for aging network nutrition professionals, ranging from the establishment of nutrition training centers and institutes, to the formation of action learning collaboratives. A sustainable and broad funding base is needed to support the training needs of aging network professionals and assure their continued acquisition of the skills, knowledge, and business acumen needed to integrate food and nutrition services into home and community-based social, health, and long-term care systems
Notes: DA - 20150625
IS - 2155-1200 (Electronic)
LA - eng
PT - Journal Article
SB - IM
SB - N
	2. 	Alburquerque-Sendin F, Barberio-Mariano E, Brandao-Santana N, Rebelatto DA, Rebelatto JR. Effects of an adapted physical activity program on the physical condition of elderly women: an analysis of efficiency. Rev.Bras.Fisioter. 2012;16:328-36.
Ref ID: 94
Keywords: Aged/Exercise Therapy/Female/Humans/Middle Aged/Physical Fitness
Reprint: Not in File
Abstract: BACKGROUND: Specific research tools and designs can assist in identifying the efficiency of physical activity in elderly women. OBJECTIVES: To identify the effects of physical activity on the physical condition of older women. METHOD: A one-year-long physical activity program (123 sessions) was implemented for women aged 60 years or older. Four physical assessments were conducted, in which weight, height, BMI, blood pressure, heart rate, absences, grip strength, flexibility, VO2max, and static and dynamic balance were assessed. The statistical analyses included a repeated measures analysis, both inferential (analysis of variance--ANOVA) and effect size (Cohen's d coefficient), as well as identification of the participants' efficiency (Data Envelopment Analysis--DEA). RESULTS: Despite the observation of differences that depended on the analysis used, the results were successful in the sense that they showed that physical activity adapted to older women can effectively change the decline in physical ability associated with aging, depending on the purpose of the study. The 60-65 yrs group was the most capable of converting physical activity into health benefits in both the short and long term. The >65 yrs group took less advantage of physical activity. CONCLUSION: Adherence to the program and actual time spent on each type of exercise are the factors that determine which population can benefit from physical activity programs. The DEA allows the assessment of the results related to time spent on physical activity in terms of health concerns. Article registered in Clinicaltrials.gov under number NCT01558401
Notes: DA - 20120817
LA - eng
PT - Clinical Trial
PT - Journal Article
SB - IM
	3. 	Ali NM, Shahar S, Kee YL, Norizan AR, Noah SA. Design of an interactive digital nutritional education package for elderly people. Inform.Health Soc.Care 2012;37:217-29.
Ref ID: 98
Keywords: Aged/Female/Health Education/organization & administration/Humans/Malaysia/Male/Middle Aged/Nutritional Status/Sex Distribution/Software Design/Surveys and Questionnaires/User-Computer Interface
Reprint: Not in File
Abstract: Designing a system for the elderly is crucial, as aging is associated with physiological changes that may impair perception, cognition and other social aspects; therefore, many aspects need consideration, especially in interface design. This study was conducted to develop a digital nutritional education package (WE Sihat) by following appropriate guidelines for elderly people to achieve better design interface and interaction. Touch-screen technology was used as a platform for user interaction. The nutritional content was based on previous nutrition studies and a lifestyle education package on healthy aging, which contains four modules. The questionnaires were distributed to 31 Malay subjects aged 60-76 years old, containing an evaluation about the overall content, graphics, design layout, colour, font size, audio/video, user-perceived satisfaction and acceptance levels. The findings showed positive feedback and acceptance. Most subjects agreed that the digital nutritional education package can increase their nutritional knowledge for a healthy lifestyle and is easy to use. The touch-screen technology was also well accepted by elderly people and can be used as a kiosk for disseminating nutrition education for healthy aging
Notes: DA - 20121205
IS - 1753-8165 (Electronic)
IS - 1753-8157 (Linking)
LA - eng
PT - Evaluation Studies
PT - Journal Article
PT - Research Support, Non-U.S. Gov't
SB - IM
	4. 	Badia T, Formiga F, Ferrer A, Sanz H, Hurtos L, Pujol R. Multifactorial assessment and targeted intervention in nutritional status among the older adults: a randomized controlled trial: the Octabaix study. BMC.Geriatr. 2015;15:45.
Ref ID: 36
Keywords: Aged/Aged,80 and over/Female/Humans/Male/Malnutrition/epidemiology/therapy/Morbidity/trends/Nutrition Assessment/Nutritional Status/Nutritional Support/methods/Retrospective Studies/Single-Blind Method/United States
Reprint: Not in File
Abstract: BACKGROUND: Malnutrition is frequent among older people and is associated with morbi-mortality. The aim of the study is to assess the effectiveness of a multifactorial and multidisciplinary intervention in the nutritional status among the elderly. METHODS: Randomized, single-blind, parallel-group, clinical trial conducted from January 2009 to December 2010 in seven primary health care centers in Baix Llobregat (Barcelona). Of 696 referred people, born in 1924, 328 subjects were randomized to an intervention group or a control group. The intervention model used an algorithm and was multifaceted for both the patients and their primary care providers. The main outcome was improvement in nutritional status assessed by Mini Nutritional Assessment (MNA). Data analyses were done by intention-to-treat. RESULTS: Two-year assessment was completed for 127 patients (77.4%) in the intervention group and 98 patients (59.7%) in the control group. In the adjusted linear mixed models for MNA, intervention showed no significant effect during all follow-up period with -0.21 (CI: - 0.96; 0.26). In subjects with nutritional risk (MNA </= 23.5/30) existed a tendency towards improvement in MNA score 1.13 (95% CI -0.48; 2.74) after 2 years. CONCLUSION: A universal multifactorial assessment and target intervention over a two year period in subjects at nutritional risk showed a tendency to improve nutrition but not in the rest of community-dwelling studied subjects. Cognitive impairment was an independent factor strongly associated with a decline in nutritional status. TRIAL REGISTRATION: The clinical trial is registered as part of a US National Institutes of Health Clinical Trial: NCT01141166
Notes: DA - 20150430
IS - 1471-2318 (Electronic)
IS - 1471-2318 (Linking)
LA - eng
PT - Journal Article
PT - Multicenter Study
PT - Randomized Controlled Trial
PT - Research Support, Non-U.S. Gov't
SB - IM
	5. 	Bann D, Chen H, Bonell C et al. Socioeconomic differences in the benefits of structured physical activity compared with health education on the prevention of major mobility disability in older adults: the LIFE study. J.Epidemiol.Community Health 2016.
Ref ID: 5
Reprint: Not in File
Abstract: BACKGROUND: Evidence is lacking on whether health-benefiting community-based interventions differ in their effectiveness according to socioeconomic characteristics. We evaluated whether the benefit of a structured physical activity intervention on reducing mobility disability in older adults differs by education or income. METHODS: The Lifestyle Interventions and Independence for Elders (LIFE) study was a multicentre, randomised trial that compared a structured physical activity programme with a health education programme on the incidence of mobility disability among at-risk community-living older adults (aged 70-89 years; average follow-up of 2.6 years). Education (</= high school (0-12 years), college (13-17 years) or postgraduate) and annual household income were self-reported (<$24 999, $25 000 to $49 999 and >/=$50 000). The risk of disability (objectively defined as loss of ability to walk 400 m) was compared between the 2 treatment groups using Cox regression, separately by socioeconomic group. Socioeconomic groupxintervention interaction terms were tested. RESULTS: The effect of reducing the incidence of mobility disability was larger for those with postgraduate education (0.72, 0.51 to 1.03; N=411) compared with lower education (high school or less (0.93, 0.70 to 1.24; N=536). However, the education groupxintervention interaction term was not statistically significant (p=0.54). Findings were in the same direction yet less pronounced when household income was used as the socioeconomic indicator. CONCLUSIONS: In the largest and longest running trial of physical activity amongst at-risk older adults, intervention effect sizes were largest among those with higher education or income, yet tests of statistical interactions were non-significant, likely due to inadequate power. TRIAL REGISTRATION NUMBER: NCT01072500
Notes: DA - 20160409
IS - 1470-2738 (Electronic)
IS - 0143-005X (Linking)
LA - ENG
PT - JOURNAL ARTICLE
	6. 	Batik O, Phelan EA, Walwick JA, Wang G, LoGerfo JP. Translating a community-based motivational support program to increase physical activity among older adults with diabetes at community clinics: a pilot study of Physical Activity for a Lifetime of Success (PALS). Prev.Chronic.Dis. 2008;5:A18.
Ref ID: 167
Keywords: Aged/Aging/physiology/Community Health Centers/organization & administration/Diabetes Mellitus,Type 2/therapy/Female/Health Promotion/Humans/Life Expectancy/Male/Motivation/Motor Activity/Outcome Assessment (Health Care)/Pilot Projects/Preventive Health Services/Program Development/Program Evaluation/Quality of Life/Social Support/Socioeconomic Factors/Washington
Reprint: Not in File
Abstract: BACKGROUND: Regular physical activity is an important goal for elders with chronic health conditions. CONTEXT: This report describes Physical Activity for a Lifetime of Success (PALS), an attempt to translate a motivational support program for physical activity, Active Choices, for use by a group of diverse, low-income, community-dwelling elders with diabetes. METHODS: PALS linked physical activity assessment and brief counseling by primary care providers with a structured referral to a community-based motivational telephone support program delivered by older adult volunteers. People with diabetes aged 65 years or older who were receiving care at two community clinics were randomized to receive either immediate or delayed intervention. The main intended outcome measure was physical activity level; the secondary outcome measure was mean hemoglobin A1c. CONSEQUENCES: One-third of those offered referral to the PALS program in the clinic setting declined. Another 44% subsequently declined enrollment or were unreachable by the support center. Only 14 (21%) of those offered referral enrolled in the program. Among these 14, the percentage who were sufficiently active was higher at follow-up than at enrollment, though not significantly so. Using an intent-to-treat analysis, which included all randomized clinic patients, we found no significant change in mean hemoglobin A1c for the intervention group compared with controls. INTERPRETATION: A community-based referral and support program to increase physical activity among elderly, ethnically diverse, low-income people with diabetes, many of whom are not English-speaking, may be thwarted by unforeseen barriers. Those who enroll and participate in the PALS program appear to increase their level of physical activity
Notes: DA - 20071217
IS - 1545-1151 (Electronic)
IS - 1545-1151 (Linking)
LA - eng
PT - Journal Article
PT - Randomized Controlled Trial
PT - Research Support, U.S. Gov't, P.H.S
SB - IM
	7. 	Beck AM, Beermann T, Kjaer S, Rasmussen HH. Ability of different screening tools to predict positive effect on nutritional intervention among the elderly in primary health care. Nutrition 2013;29:993-9.
Ref ID: 75
Keywords: Body Mass Index/Humans/Malnutrition/diagnosis/Nutrition Assessment/Nutritional Status/Primary Health Care/methods/Randomized Controlled Trials as Topic/Reproducibility of Results/Risk Assessment/Risk Factors
Reprint: Not in File
Abstract: OBJECTIVE: Routine identification of nutritional risk screening is paramount as the first stage in nutritional treatment of the elderly. The major focus of former validation studies of screening tools has been on the ability to predict undernutrition. The aim of this study was to validate Mini Nutritional Assessment-Short Form (MNA-SF), the Malnutrition Universal Screening Tool (MUST), the Nutritional Risk Screening 2002 (NRS-2002), Body Mass Index (BMI) <24, and the Eating Validation Scheme (EVS), using published randomized controlled trials of nutritional intervention among old people in primary health care, in order to evaluate whether they were capable of distinguishing those with a positive benefit from those that showed no benefit of nutritional intervention. METHODS: The methods used were a literature search; classification of participants with respect to nutritional risk according to the different nutritional screening tools; and validation (i.e., evaluation of whether the different tools were capable of distinguishing those with a positive benefit from those that showed no benefit of nutritional intervention by assessing the positive [PPV] and negative [NPV] predictive values). RESULTS: MNA-SF, NRS-2002, BMI <24 and EVS had the highest PPV (0.75) and EVS the highest NPV (0.74) with regard to function-the primary clinical outcome. CONCLUSION: Overall EVS seemed most capable of distinguishing those clients and residents with a positive benefit from those that showed no benefit of nutritional intervention. The findings should be confirmed in further validation and intervention studies
Notes: DA - 20130613
IS - 1873-1244 (Electronic)
IS - 0899-9007 (Linking)
LA - eng
PT - Journal Article
SB - IM
	8. 	Becofsky K, Baruth M, Wilcox S. Physical activity mediates the relationship between program participation and improved mental health in older adults. Public Health 2016;132:64-71.
Ref ID: 23
Reprint: Not in File
Abstract: OBJECTIVES: There is an implicit assumption that increased physical activity (PA) levels are responsible for the mental health benefits resulting from participation in PA programs. Other factors associated with participation may in fact be responsible. The purpose of this study was to examine whether changes in PA mediated the effects of two PA programs (Active Choices [AC] and Active Living Every Day [ALED]) on mental health outcomes. STUDY DESIGN: Secondary data analyses of quasi-experimental study. METHODS: A sub-sample of older adults who participated in AC (n = 744) and ALED (n = 853) were included in the current analyses. MacKinnon's product of coefficients was used to test change in PA as a mediator of the relationship between program dose and change in mental health outcomes (depressive symptoms, stress, and number of days with poor mental health). RESULTS: Change in PA explained 19% (AC) and 13% (ALED) of the absolute effects of program dose on depressive symptoms, 18% (AC) and 14% (ALED) of the effects on stress, and 37% (ALED) of the effects on days with poor mental health. CONCLUSIONS: Mounting evidence from both epidemiological studies and controlled trials suggests that PA can improve mental health. This study adds that increasing PA levels may improve mental health in older adults in 'real-world' settings
Notes: DA - 20160318
IS - 1476-5616 (Electronic)
IS - 0033-3506 (Linking)
LA - eng
PT - Journal Article
SB - IM
	9. 	Berendsen A, Santoro A, Pini E et al. Reprint of: A parallel randomized trial on the effect of a healthful diet on inflammageing and its consequences in European elderly people: design of the NU-AGE dietary intervention study. Mech.Ageing Dev. 2014;136-137:14-21.
Ref ID: 52
Reprint: Not in File
Abstract: BACKGROUND: The proportion of European elderly is expected to increase to 30% in 2060. Combining dietary components may modulate many processes involved in ageing. So, it is likely that a healthful diet approach might have greater favourable impact on age-related decline than individual dietary components. This paper describes the design of a healthful diet intervention on inflammageing and its consequences in the elderly. METHODS: The NU-AGE study is a parallel randomized one-year trial in 1250 apparently healthy, independently living European participants aged 65-80 years. Participants are randomised into either the diet group or control group. Participants in the diet group received dietary advice aimed at meeting the nutritional requirements of the ageing population. Special attention was paid to nutrients that may be inadequate or limiting in diets of elderly, such as vitamin D, vitamin B12, and calcium. C-reactive protein is measured as primary outcome. DISCUSSION: The NU-AGE study is the first dietary intervention investigating the effect of a healthful diet providing targeted nutritional recommendations for optimal health and quality of life in apparently healthy European elderly. Results of this intervention will provide evidence on the effect of a healthful diet on the prevention of age related decline
Notes: DA - 20140505
IS - 1872-6216 (Electronic)
IS - 0047-6374 (Linking)
LA - eng
PT - Journal Article
PT - Research Support, Non-U.S. Gov't
	10. 	Best JR, Liu-Ambrose T. A 2-year physical activity program for sedentary older adults does not improve cognitive functioning more than a health education program [commentary]. J.Physiother. 2016;62:115.
Ref ID: 6
Reprint: Not in File
Notes: DA - 20160408
IS - 1836-9561 (Electronic)
IS - 1836-9561 (Linking)
LA - eng
PT - Journal Article
SB - IM
	11. 	Bethancourt HJ, Rosenberg DE, Beatty T, Arterburn DE. Barriers to and facilitators of physical activity program use among older adults. Clin.Med.Res. 2014;12:10-20.
Ref ID: 55
Keywords: Aged/Exercise/Female/Fitness Centers/Focus Groups/Health Promotion/Health Services Accessibility/Humans/Life Style/Male/Medicare/Motivation/Qualitative Research/United States/Washington
Reprint: Not in File
Abstract: OBJECTIVE: Regular physical activity (PA) is important for maintaining long-term physical, cognitive, and emotional health. However, few older adults engage in routine PA, and even fewer take advantage of programs designed to enhance PA participation. Though most managed Medicare members have free access to the Silver Sneakers and EnhanceFitness PA programs, the vast majority of eligible seniors do not utilize these programs. The goal of this qualitative study was to better understand the barriers to and facilitators of PA and participation in PA programs among older adults. DESIGN: This was a qualitative study using focus group interviews. SETTING: Focus groups took place at three Group Health clinics in King County, Washington. PARTICIPANTS: Fifty-two randomly selected Group Health Medicare members between the ages of 66 to 78 participated. METHODS: We conducted four focus groups with 13 participants each. Focus group discussions were audio-recorded, transcribed, and analyzed using an inductive thematic approach and a social-ecological framework. RESULTS: Men and women were nearly equally represented among the participants, and the sample was largely white (77%), well-educated (69% college graduates), and relatively physically active. Prominent barriers to PA and PA program participation were physical limitations due to health conditions or aging, lack of professional guidance, and inadequate distribution of information on available and appropriate PA options and programs. Facilitators included the motivation to maintain physical and mental health and access to affordable, convenient, and stimulating PA options. CONCLUSION: Older adult populations may benefit from greater support and information from their providers and health care systems on how to safely and successfully improve or maintain PA levels through later adulthood. Efforts among health care systems to boost PA among older adults may need to consider patient-centered adjustments to current PA programs, as well as alternative methods for promoting overall active lifestyle choices
Notes: DA - 20141029
IS - 1554-6179 (Electronic)
IS - 1539-4182 (Linking)
LA - eng
PT - Journal Article
PT - Research Support, Non-U.S. Gov't
SB - IM
	12. 	Brand T, Gansefort D, Rothgang H, Roseler S, Meyer J, Zeeb H. Promoting community readiness for physical activity among older adults in Germany - protocol of the ready to change intervention trial. BMC.Public Health 2016;16:99.
Ref ID: 11
Reprint: Not in File
Abstract: BACKGROUND: Healthy ageing is an important concern for many societies facing the challenge of an ageing population. Physical activity (PA) is a major contributor to healthy ageing; however insufficient PA levels are prevalent in old age in Germany. Community capacity building and community involvement are often recommended as key strategies to improve equitable access to prevention and health promotion. However, evidence for the effectiveness of these strategies is scarce. This study aims to assess the community readiness for PA promotion in local environments and to analyse the utility of strategies to increase community readiness for reaching vulnerable groups. METHODS/DESIGN: We designed a mixed method intervention trial comprising three study modules. The first module includes an assessment of community readiness for PA interventions in older adults. The assessment is carried out in a sample of 24 municipalities in the Northwest of Germany using structured key informant interviews. In the second module, eight municipalities with the low community readiness are selected from the sample and randomly assigned to one of two study groups: active enhancement of community readiness (intervention) versus no enhancement (control). After enhancing community readiness in the active enhancement group, older adults in both study groups will be recruited for participation in a PA intervention. Participation rates are compared between the study groups to evaluate the effects of the intervention. In addition, a cost-effectiveness analysis is carried out calculating recruitment costs per person reached in the two study groups. In the third module, qualitative interviews are conducted with participants and non-participants of the PA intervention exploring reasons for participation or non-participation. DISCUSSION: This study offers the potential to contribute to the evidence base of reaching vulnerable older adults for PA interventions and provide ideas on how to reduce participation barriers. Its findings will inform governmental authorities, professionals, academics, and NGOs with an estimate of resources necessary to achieve equitable access to physical activity programs for vulnerable older adults. TRIAL REGISTRATION: German Clinical Trials Register DRKS00009564 (Date of registration 03-11-2015)
Notes: DA - 20160202
IS - 1471-2458 (Electronic)
IS - 1471-2458 (Linking)
LA - eng
PT - Journal Article
SB - IM
	13. 	Broekhuizen K, de Gelder J, Wijsman CA et al. An Internet-Based Physical Activity Intervention to Improve Quality of Life of Inactive Older Adults: A Randomized Controlled Trial. J.Med.Internet.Res. 2016;18:e74.
Ref ID: 1
Reprint: Not in File
Abstract: BACKGROUND: Increasing physical activity is a viable strategy for improving both the health and quality of life of older adults. OBJECTIVE: The aim of this study was to assess if an Internet-based intervention aimed to increase physical activity was effective in improving quality of life of inactive older adults. In addition, we analyzed the effect of the intervention on quality of life among those participants who successfully reached their individually targeted increase in daily physical activity as indicated by the intervention program, as well as the dose-response effect of increasing physical activity on quality of life. METHODS: The intervention was tested in a randomized controlled trial and was comprised of an Internet program-DirectLife (Philips)-aimed at increasing physical activity using monitoring and feedback by accelerometry and feedback by digital coaching (n=119). The control group received no intervention (n=116). Participants were inactive 60-70-year-olds and were recruited from the general population. Quality of life and physical activity were measured at baseline and after 3 months using the Research ANd Development 36-item health survey (RAND-36) and wrist-worn triaxial accelerometer, respectively. RESULTS: After 3 months, a significant improvement in quality of life was seen in the intervention group compared to the control group for RAND-36 subscales on emotional and mental health (2.52 vs -0.72, respectively; P=.03) and health change (8.99 vs 2.03, respectively; P=.01). A total of 50 of the 119 participants (42.0%) in the intervention group successfully reached their physical activity target and showed a significant improvement in quality of life compared to the control group for subscales on emotional and mental health (4.31 vs -0.72, respectively; P=.009) and health change (11.06 vs 2.03, respectively; P=.004). The dose-response analysis showed that there was a significant association between increase in minutes spent in moderate-to-vigorous physical activity (MVPA) and increase in quality of life. CONCLUSIONS: Our study shows that an Internet-based physical activity program was effective in improving quality of life in 60-70-year-olds after 3 months, particularly in participants that reached their individually targeted increase in daily physical activity. TRIAL REGISTRATION: Nederlands Trial Register: NTR 3045; http://www.trialregister.nl/trialreg/admin/rctview.asp?TC=3045 (Archived by WebCite at http://www.webcitation.org/6fobg2sjJ)
Notes: DA - 20160428
IS - 1438-8871 (Electronic)
IS - 1438-8871 (Linking)
LA - eng
PT - Journal Article
SB - IM
	14. 	Buford TW, Miller ME, Church TS et al. Antihypertensive Use and the Effect of a Physical Activity Intervention in the Prevention of Major Mobility Disability Among Older Adults: The LIFE Study. J.Gerontol.A Biol.Sci.Med.Sci. 2016.
Ref ID: 10
Reprint: Not in File
Abstract: BACKGROUND: This subgroup analysis of the Lifestyle Intervention and Independence for Elders trial evaluates the impact of a long-term physical activity (PA) intervention on rates of major mobility disability (MMD) among older adults according to their antihypertensive medication use. METHODS: Lifestyle Intervention and Independence for Elders study participants were randomized to center-based PA or health education for a median of 2.7 years. Participants were sedentary men and women aged 70-89 years with objectively measured physical limitations. This analysis evaluated rates of MMD and persistent MMD among 1,633 participants, according to antihypertensive medication use. Participants were designated as either (i) an angiotensin-converting enzyme (ACE) inhibitor user (ACEi+), (ii) a user of other antihypertensives not including ACEi (ACEi-), or (iii) nonusers of antihypertensive medications (AHT-). Interactions were explored between antihypertensive use and randomized arm. RESULTS: Interaction terms for MMD (p = .214) and persistent MMD (p = .180) did not reach statistical significance. For MMD, PA displayed marginal effects among ACEi+ (hazard ratio [HR] = 0.76; 95% confidence interval [CI] = 0.57, 1.02) and ACEi- (HR = 0.76; 95% CI = 0.60, 0.97) but not AHT- (HR = 1.19; 95% CI = 0.75, 1.87). For persistent MMD, the effect of PA was greatest among ACEi+ (HR = 0.57; 95% CI = 0.39, 0.84) when compared to ACEi- (HR = 0.76; 95% CI = 0.55, 1.06) or AHT- (HR = 1.18; 95% CI = 0.59, 2.36). CONCLUSIONS: The effects of long-term PA on the incidence of MMD and persistent MMD were similar among three subgroups of older adults stratified by their antihypertensive medication use. However, though statistical interactions did not reach significance, several findings may warrant future study in other cohorts given the post hoc nature of this study
Notes: DA - 20160211
IS - 1758-535X (Electronic)
IS - 1079-5006 (Linking)
LA - ENG
PT - JOURNAL ARTICLE
	15. 	Burke L, Lee AH, Pasalich M, Jancey J, Kerr D, Howat P. Effects of a physical activity and nutrition program for seniors on body mass index and waist-to-hip ratio: a randomised controlled trial. Prev.Med. 2012;54:397-401.
Ref ID: 100
Keywords: Aged/Anthropometry/Australia/Body Mass Index/Diet/Exercise/physiology/Female/Health Promotion/methods/Health Services for the Aged/Home Care Services/organization & administration/Humans/Male/Middle Aged/Nutritional Status/Obesity/diet therapy/prevention & control/Program Evaluation/Social Class/Surveys and Questionnaires/Time Factors/Urban Population/Waist-Hip Ratio/Western Australia
Reprint: Not in File
Abstract: OBJECTIVE: To investigate whether a home-based program, physical activity and nutrition for seniors (PANS), made positive changes to central obesity, measured by body mass index (BMI) and waist-to-hip ratio (WHR). METHODS: A 6-month randomised controlled trial was conducted targeting overweight and sedentary older adults aged 60 to 70 years residing in low to medium socio-economic suburbs within metropolitan Perth. Intervention participants (n=248) received mailed materials and telephone/email support to improve nutrition and physical activity levels. Controls (n=230) received small incentives to complete baseline and post-intervention questionnaires. Both groups reported anthropometric measures following specific written instructions. Generalised estimating equation models were used to assess repeated outcomes of BMI and WHR over both time points. RESULTS: 176 intervention and 199 controls (response rate 78.5%) with complete data were available for analysis. After controlling for demographic and other confounding factors, the intervention group demonstrated a small (0.02) but significant reduction in WHR (p=0.03) compared to controls, no apparent change in BMI was evident for both groups. The 0.02 reduction in mean WHR corresponded to a 2.11 cm decrease in waist circumference for a typical hip circumference. CONCLUSION: PANS appears to improve the WHR of participants. Changes in BMI might require a longer term intervention to take effect, and/or a follow-up study to confirm its sustainability
Notes: DA - 20120605
IS - 1096-0260 (Electronic)
IS - 0091-7435 (Linking)
LA - eng
PT - Journal Article
PT - Randomized Controlled Trial
PT - Research Support, Non-U.S. Gov't
SB - IM
	16. 	Burke L, Jancey J, Howat P et al. Physical activity and nutrition program for seniors (PANS): protocol of a randomized controlled trial. BMC.Public Health 2010;10:751.
Ref ID: 127
Keywords: Aged/Diet/Exercise/Health Promotion/organization & administration/Humans/Middle Aged/Models,Theoretical/Nutritional Requirements/Pamphlets/Research Design/Western Australia
Reprint: Not in File
Abstract: BACKGROUND: Along with reduced levels of physical activity, older Australian's mean energy consumption has increased. Now over 60% of older Australians are considered overweight or obese. This study aims to confirm if a low-cost, accessible physical activity and nutrition program can improve levels of physical activity and diet of insufficiently active 60-70 year-olds. METHODS/DESIGN: This 12-month home-based randomised controlled trial (RCT) will consist of a nutrition and physical activity intervention for insufficiently active people aged 60 to 70 years from low to medium socio-economic areas. Six-hundred participants will be recruited from the Australian Federal Electoral Role and randomly assigned to the intervention (n = 300) and control (n = 300) groups. The study is based on the Social Cognitive Theory and Precede-Proceed Model, incorporating voluntary cooperation and self-efficacy. The intervention includes a specially designed booklet that provides participants with information and encourages dietary and physical activity goal setting. The booklet will be supported by an exercise chart, calendar, bi-monthly newsletters, resistance bands and pedometers, along with phone and email contact. Data will be collected over three time points: pre-intervention, immediately post-intervention and 6-months post-study. DISCUSSION: This trial will provide valuable information for community-based strategies to improve older adults' physical activity and dietary intake. The project will provide guidelines for appropriate sample recruitment, and the development, implementation and evaluation of a minimal intervention program, as well as information on minimising barriers to participation in similar programs. TRIAL REGISTRATION: Australian and New Zealand Clinical Trials Registry ACTRN12609000735257
Notes: DA - 20110106
IS - 1471-2458 (Electronic)
IS - 1471-2458 (Linking)
LA - eng
PT - Journal Article
PT - Randomized Controlled Trial
PT - Research Support, Non-U.S. Gov't
SB - IM
	17. 	Burke L, Lee AH, Jancey J et al. Physical activity and nutrition behavioural outcomes of a home-based intervention program for seniors: a randomized controlled trial. Int.J.Behav.Nutr Phys.Act. 2013;10:14.
Ref ID: 82
Keywords: Aged/Diet/Dietary Fats/administration & dosage/Female/Food Habits/Fruit/Health Behavior/Health Promotion/methods/Humans/Male/Middle Aged/Physical Exertion/Posture/Resistance Training/Sedentary Lifestyle/Social Class/Surveys and Questionnaires/Walking
Reprint: Not in File
Abstract: BACKGROUND: This intervention aimed to ascertain whether a low-cost, accessible, physical activity and nutrition program could improve physical activity and nutrition behaviours of insufficiently active 60-70 year olds residing in Perth, Australia. METHODS: A 6-month home-based randomised controlled trial was conducted on 478 older adults (intervention, n = 248; control, n = 230) of low to medium socioeconomic status. Both intervention and control groups completed postal questionnaires at baseline and post-program, but only the intervention participants received project materials. A modified fat and fibre questionnaire measured nutritional behaviours, whereas physical activity was measured using the International Physical Activity Questionnaire. Generalised estimating equation models were used to assess the repeated outcomes over both time points. RESULTS: The final sample consisted of 176 intervention participants and 199 controls (response rate 78.5%) with complete data. After controlling for demographic and other confounding factors, the intervention group demonstrated increased participation in strength exercise (p < 0.001), walking (p = 0.029) and vigorous activity (p = 0.015), together with significant reduction in mean sitting time (p < 0.001) relative to controls. Improvements in nutritional behaviours for the intervention group were also evident in terms of fat avoidance (p < 0.001), fat intake (p = 0.021) and prevalence of frequent fruit intake (p = 0.008). CONCLUSIONS: A minimal contact, low-cost and home-based physical activity program can positively influence seniors' physical activity and nutrition behaviours. TRIAL REGISTRATION: anzctr.org.au Identifier: ACTRN12609000735257
Notes: DA - 20130212
IS - 1479-5868 (Electronic)
IS - 1479-5868 (Linking)
LA - eng
PT - Journal Article
PT - Randomized Controlled Trial
PT - Research Support, Non-U.S. Gov't
RN - 0 (Dietary Fats)
SB - IM
	18. 	Burke L, Jancey JM, Howat P, Lee AH, Shilton T. Physical Activity and Nutrition Program for Seniors (PANS): process evaluation. Health Promot.Pract. 2013;14:543-51.
Ref ID: 90
Keywords: Aged/Body Mass Index/Diet/Exercise/Female/Health Promotion/organization & administration/Humans/Male/Middle Aged/Monitoring,Ambulatory/Pamphlets/Program Evaluation/Residence Characteristics/Telephone
Reprint: Not in File
Abstract: ISSUE ADDRESSED: The Physical Activity and Nutrition Program for Seniors (PANS) program aimed to increase levels of physical activity and improve the diet of insufficiently active community-based seniors aged 60 to 70 years using a range of strategies. Comprehensive process evaluation was used to determine the suitability and appropriateness of the resources and effectiveness of the strategies. METHOD: Process evaluation data (qualitative and quantitative) were collected on the program strategies and resources throughout, and at the conclusion of the intervention period. RESULTS: The program strategies/resources were found to be relevant to the population, assisting participants to increase their level of physical activity and improve their diet. Participants reported that the program resources were suitable for their age-group (84%), encouraged them to think about physical activity (78%), and nutrition (70%). Participants reported that they used the pedometer (91%) and recorded daily steps (78%). Moreover, the provision of group guides facilitated individuals to set and achieve personal goals. CONCLUSION: The PANS strategies and resources were appropriate, which supported the seniors in identifying, establishing, and achieving their physical activity and nutrition goals. Minor refinements of the program were recommended based on the findings
Notes: DA - 20130607
IS - 1524-8399 (Print)
IS - 1524-8399 (Linking)
LA - eng
PT - Journal Article
PT - Research Support, Non-U.S. Gov't
SB - IM
	19. 	Choi SH, Choi-Kwon S. The effects of the DASH diet education program with omega-3 fatty acid supplementation on metabolic syndrome parameters in elderly women with abdominal obesity. Nutr Res.Pract. 2015;9:150-7.
Ref ID: 37
Reprint: Not in File
Abstract: BACKGROUND/OBJECTIVES: The purpose of this study was to investigate the overall effects of a tailored Dietary Approaches to Stop Hypertension (DASH) nutritional intervention program which included omega-3 fatty acids supplementation, on dietary self-efficacy, dietary knowledge, and dietary behaviors in Korean elderly women with abdominal obesity. Furthermore, we investigated the effects of the program on metabolic syndrome parameters including the antioxidant capacities in these subjects. SUBJECTS/METHODS: A randomized, controlled trial was conducted for 8 weeks. The experimental group (n = 21) received a weekly tailored nutritional program for 8 weeks and the control group (n = 18) received only one educational session. The clinical survey was conducted before and after the intervention period. RESULTS: After the intervention, dietary self-efficacy (P = 0.023), frequency of fruit intake (P = 0.019), and dietary fiber intake (P = 0.044) were higher in the experimental group than in the control group. The oxidative stress (P < 0.001) was lower in the experimental group than in the control group. Moreover, low density lipoprotein (LDL) cholesterol (P = 0.023) had significantly decreased in the experimental group but not in the control group after the intervention. CONCLUSIONS: The intervention program including omega-3 fatty acid supplementation had a positive effect on dietary self-efficacy, dietary behaviors, and oxidative stress among aged women with abdominal obesity
Notes: DA - 20150411
IS - 1976-1457 (Print)
IS - 1976-1457 (Linking)
LA - eng
PT - Journal Article
	20. 	Chung LM, Chung JW. Effectiveness of a food education program in improving appetite and nutritional status of elderly adults living at home. Asia Pac.J.Clin.Nutr 2014;23:315-20.
Ref ID: 50
Keywords: Aged/Aged,80 and over/Analysis of Variance/Appetite/physiology/Cooking/methods/statistics & numerical data/Diet/Female/Follow-Up Studies/Geriatric Assessment/Health Education/Hong Kong/Humans/Male/Middle Aged/Nutrition Surveys/Nutritional Status/Personal Satisfaction/Program Evaluation/Residence Characteristics/Surveys and Questionnaires
Reprint: Not in File
Abstract: OBJECTIVE: To evaluate a 3-week program comprising cooking demonstrations with free food samples in motivating elderly adults to cook more and improve their nutritional status. DESIGN: An experimental pre-post study. SETTING: Three districts in Hong Kong. PARTICIPANTS: Sixty aged 59-95 home-living adults. INTERVENTION: Group A (one 1-day food sample given free weekly) and Group B (three 1-day food samples given free weekly). MAIN OUTCOME MEASURE: Satisfaction questionnaires were conducted every week. Mini Nutritional Assessment (MNA) scores were assessed at baseline and 6 months after the program's completion. ANALYSIS: Nutritional status was assessed before and after intervention. Repeated analysis of variance measures of compliance, appetite, easiness of program at 3-week time-points were calculated to differentiate a more frequent (Group B) and a less frequent (Group A) provision of food sample reinforced their cooking skills to a greater extent. RESULTS: The compliance rate of Group B was higher than that of Group A. More than 60% of the participants intended to continue cooking and a third of the participants expressed satisfaction with the program. The MNA scores had improved 6 months later (combined data from both groups). CONCLUSIONS: Nutrition education through cooking demonstrations and the community-based distribution of food ingredients can improve the nutrition status of the elderly population
Notes: DA - 20140606
IS - 0964-7058 (Print)
IS - 0964-7058 (Linking)
LA - eng
PT - Journal Article
SB - IM
	21. 	Comte M, Hobin E, Manske S et al. Is the Provision of Physical Education to Senior-Years Students Associated With Greater Physical Activity Levels? Insight Into a Province-Wide Policy. J.Phys.Act.Health 2015;12:649-54.
Ref ID: 46
Keywords: Accelerometry/Adolescent/Cross-Sectional Studies/Exercise/Female/Health Policy/Humans/Male/Physical Education and Training/Students/psychology
Reprint: Not in File
Abstract: OBJECTIVES: The purpose of this study was to determine if participation in physical education (PE) was associated with increased moderate-to-vigorous physical activity (MVPA) levels in adolescents. METHODS: This was a cross sectional study comparing MVPA levels in senior-years students-grade 11 and 12-enrolled in high school PE during the semester data were collected compared with those not enrolled in PE in that same semester. The primary outcome measure was daily MVPA measured by accelerometry. The primary exposure was participation in PE. RESULTS: Among the 508 adolescents (16.9 +/- 0.8 yrs, 49% female, n = 338 exposed to PE) studied, no differences in MVPA (47.0 +/- 25.8 vs. 43.9 +/- 25.0 mins/day, P = .25) or sedentary time (540.2 +/- 94.7 vs. 550.2 +/- 79.4 mins/day, P = .79) were noted between students enrolled in PE compared with students not enrolled in PE. Participation in PE was associated with a greater odds of achieving >60 minutes of MVPA daily (OR: 1.69; 95% CI: 1.04-2.75). This association was stronger among boys (OR: 2.4; 95% CI: 1.2-4.8) than girls (OR: 1.17; 95% CI: 0.5-2.7). CONCLUSION: Enrollment in PE in grade 11 or 12 is associated with modestly higher levels of MVPA and an increased likelihood of meeting PA guidelines among students in grades 11 and 12, particularly among boys
Notes: DA - 20150804
IS - 1543-5474 (Electronic)
IS - 1543-3080 (Linking)
LA - eng
PT - Journal Article
PT - Research Support, Non-U.S. Gov't
SB - IM
	22. 	Cottell KE, Dorfman LR, Straight CR, Delmonico MJ, Lofgren IE. The effects of diet education plus light resistance training on coronary heart disease risk factors in community-dwelling older adults. J.Nutr Health Aging 2011;15:762-7.
Ref ID: 109
Keywords: Adipose Tissue/metabolism/Aged/Aged,80 and over/Blood Pressure/Body Composition/Body Mass Index/Body Weight/Coronary Disease/complications/diet therapy/prevention & control/Diet/Female/Humans/Male/Middle Aged/Obesity/Patient Education as Topic/Resistance Training/Rhode Island/Risk Factors/Surveys and Questionnaires/Waist Circumference
Reprint: Not in File
Abstract: OBJECTIVES: To examine the effects of diet education (DE) plus light resistance training (RT) on coronary heart disease risk (CHD) factors, specifically dietary quality, blood lipid and C-Reactive protein (CRP) concentrations in overweight and obese older adults in a community setting. DESIGN: Community outreach intervention with a quasi-experimental design. PARTICIPANTS AND SETTING: A total of 96 subjects, 16 males and 80 females, aged 69.2 +/- 6.2 years, community-dwelling, and from one of four senior centers in Rhode Island. INTERVENTION: Subjects participated in 30 minutes of DE (once per week) and ~80 minutes of RT (two separate sessions per week) for eight weeks. The DE sessions were led by a registered dietitian. MEASUREMENTS: Anthropometrics (height, weight, waist circumference, hip circumference, and body composition), clinical (blood pressure), biochemical (lipid profile, glucose, and CRP concentrations), and diet quality measured by the Dietary Screening Tool (DST). RESULTS: A significant change was seen in DST risk categories from baseline to post-intervention, chi(2) (2)=20.43, p < 0.01. Significant differences were seen in triacylglycerol (p=0.028) as well as in systolic and diastolic blood pressures, weight, waist circumference, hip circumference, percent body fat, fat mass, and body mass index (all p<0.05). CONCLUSION: This intervention effectively decreased CHD risk in overweight and obese older adults. Future research is needed to examine the effects of longer DE plus RT interventions with greater weight loss on the lipid profile and CRP concentrations in overweight and obese older adults at risk for CHD
Notes: DA - 20111117
IS - 1760-4788 (Electronic)
IS - 1279-7707 (Linking)
LA - eng
PT - Journal Article
SB - IM
	23. 	Cox KL, Flicker L, Almeida OP et al. The FABS trial: a randomised control trial of the effects of a 6-month physical activity intervention on adherence and long-term physical activity and self-efficacy in older adults with memory complaints. Prev.Med. 2013;57:824-30.
Ref ID: 63
Keywords: Aged/Exercise/psychology/Exercise Therapy/methods/Female/Humans/Male/Memory Disorders/therapy/Middle Aged/Mild Cognitive Impairment/Motor Activity/Patient Compliance/statistics & numerical data/Self Efficacy/Sex Factors
Reprint: Not in File
Abstract: OBJECTIVE: The aim of this study is to assess in older adults with memory complaints, the effects of a 6-month home-based physical activity (PA) intervention on short-term adherence, short and long-term self-efficacy and the predictors of adherence. METHODS: Participants with memory complaints with or without mild cognitive impairment (MCI) were recruited from Perth, Western Australia between May 2004 and July 2006 and randomly assigned to a control or an intervention group. The intervention group received a 6-month PA programme and recorded sessions on a diary. Pedometer readings, questionnaires, and physical and cognitive measures were completed at 0, 6, 12 and 18 months. RESULTS: One hundred and seventy participants started the study. Retention rates were similar for both groups at all time-points however retention was higher for men than women (P<0.01). Adherence to the prescribed PA was 72.8% (95% CI, 70.8 74.9%). Men had higher adherence rate than women (P<0.001). Those with and without MCI had similar adherence. Compared to controls self-efficacy was higher in the intervention group after 6 months only (P<0.01). CONCLUSIONS: Older adults with memory complaints, with or without MCI, can successfully participate in and enjoy home-based PA programmes. Long-term adherence to such interventions may require continued support and increased self-efficacy. ( TRIAL REGISTRATION: ACTRN012605000136606.)
Notes: DA - 20131122
IS - 1096-0260 (Electronic)
IS - 0091-7435 (Linking)
LA - eng
PT - Journal Article
PT - Randomized Controlled Trial
PT - Research Support, Non-U.S. Gov't
SB - IM
	24. 	Daly RM, Gianoudis J, Prosser M et al. The effects of a protein enriched diet with lean red meat combined with a multi-modal exercise program on muscle and cognitive health and function in older adults: study protocol for a randomised controlled trial. Trials 2015;16:339.
Ref ID: 27
Keywords: Age Factors/Aged/Aging/psychology/Biomarkers/blood/Clinical Protocols/Cognition/Cognition Disorders/diagnosis/etiology/prevention & control/Combined Modality Therapy/Dietary Proteins/administration & dosage/Female/Geriatric Assessment/Humans/Male/Mental Health/Muscle Strength/Muscle,Skeletal/pathology/physiopathology/Quality of Life/Red Meat/Research Design/Resistance Training/Sarcopenia/Surveys and Questionnaires/Time Factors/Treatment Outcome/Victoria
Reprint: Not in File
Abstract: BACKGROUND: Age-related muscle wasting has been strongly implicated with falls and fractures in the elderly, but it has also been associated with cognitive decline and dementia. Progressive resistance training (PRT) and adequate dietary protein are recognised as important contributors to the maintenance of muscle health and function in older adults. However, both factors also have the potential to improve brain function and prevent cognitive decline via several pathways, including the regulation of various growth and neurotrophic factors [insulin-like growth factor-1 (IGF-1)]; brain-derived growth factor (BDNF)] and/or the modulation of systemic inflammation. The primary aim of this study is to investigate whether a modest increase in dietary protein achieved through the consumption of lean red meat three days per week, when combined with PRT, can enhance muscle mass, size and strength and cognitive function in community-dwelling older people. METHODS/DESIGN: The study design is a 48-week randomised controlled trial consisting of a 24-week intervention with a 24-week follow-up. Men and women (n=152) aged 65 years and over residing in the community will be randomly allocated to: 1) PRT and provided with 220 g (raw weight) of lean red meat to be cooked and divided into two 80 g servings on each of the three days that they complete their exercise session, or 2) control PRT in which participants will be provided with and advised to consume >/=1 serving (~1/2 cup) of rice and/or pasta or 1 medium potato on each of the three training days. The primary outcome measures will be muscle mass, size and strength and cognitive function. Secondary outcomes will include changes in: muscle function, neural health (corticospinal excitability and inhibition and voluntary activation), serum IGF-1 and BDNF, adipokines and inflammatory markers, fat mass and inter-/intra-muscular fat, blood pressure, lipids and health-related quality of life. All outcome measures will be assessed at baseline and 24 weeks, with the exception of cognitive function and the various neurobiological and inflammatory markers which will also be assessed at week 12. DISCUSSION: The findings from this study will provide important new information on whether a modest increase in dietary protein achieved through the ingestion of lean red meat can enhance the effects of PRT on muscle mass, size and strength as well as cognitive function in community-dwelling older adults. If successful, the findings will form the basis for more precise exercise and nutrition guidelines for the management and prevention of age-related changes in muscle and neural health and cognitive function in the elderly. TRIAL REGISTRATION: Australian New Zealand Clinical Trials Registry: ACTRN12613001153707 . Date registered 16(th) October, 2013
Notes: DA - 20150808
IS - 1745-6215 (Electronic)
IS - 1745-6215 (Linking)
LA - eng
PT - Journal Article
PT - Randomized Controlled Trial
PT - Research Support, Non-U.S. Gov't
RN - 0 (Biomarkers)
RN - 0 (Dietary Proteins)
SB - IM
	25. 	Espeland MA, Rapp SR, Katula JA et al. Telephone interview for cognitive status (TICS) screening for clinical trials of physical activity and cognitive training: the seniors health and activity research program pilot (SHARP-P) study. Int.J.Geriatr.Psychiatry 2011;26:135-43.
Ref ID: 126
Keywords: Aged/Aged,80 and over/Cognition/physiology/Cognition Disorders/diagnosis/prevention & control/Cognitive Therapy/Exercise/psychology/Female/Geriatric Assessment/methods/Humans/Logistic Models/Male/Mass Screening/Physical Exertion/Pilot Projects/Remote Consultation/Telephone
Reprint: Not in File
Abstract: OBJECTIVE: To examine the performance of the Telephone Interview for Cognitive Status (TICS) for identifying participants appropriate for trials of physical activity and cognitive training interventions. METHODS: Volunteers (N=343), ages 70-85 years, who were being recruited for a pilot clinical trial on approaches to prevent cognitive decline, were administered TICS and required to score >/= 31 prior to an invitation to attend clinic-based assessments. The frequencies of contraindications for physical activity and cognitive training interventions were tallied for individuals grouped by TICS scores. Relationships between TICS scores and other measures of cognitive function were described by scatterplots and correlation coefficients. RESULTS: Eligibility criteria to identify candidates who were appropriate candidates for the trial interventions excluded 51.7% of the volunteers with TICS<31. TICS scores above this range were not strongly related to cognition or attendance at screening visits, however overall enrollment yields were approximately half for participants with TICS=31 versus TICS=41, and increased in a graded fashion throughout the range of scores. CONCLUSIONS: Use of TICS to define eligibility criteria in trials of physical activity and cognitive training interventions may not be worthwhile in that many individuals with low scores would already be eliminated by intervention-specific criteria and the relationship of TICS with clinic-based tests of cognitive function among appropriate candidates for these interventions may be weak. TICS may be most useful in these trials to identify candidates for oversampling in order to obtain a balanced cohort of participants at risk for cognitive decline
Notes: DA - 20110113
IS - 1099-1166 (Electronic)
IS - 0885-6230 (Linking)
LA - eng
PT - Journal Article
PT - Research Support, N.I.H., Extramural
SB - IM
	26. 	Estabrooks PA, Smith-Ray RL, Dzewaltowski DA et al. Sustainability of evidence-based community-based physical activity programs for older adults: lessons from Active for Life. Transl.Behav.Med. 2011;1:208-15.
Ref ID: 119
Reprint: Not in File
Abstract: Program sustainability in community and healthcare settings is critical to realizing the translation of research into practice. The purpose of this study is to describe the implementation and assessment of an intervention to increase organizational maintenance of evidence-based physical activity programs and the factors that impede or facilitate sustainability. All organizations implemented a sustainability action plan that included identifying factors related to sustainability, examining resources available, identifying program modifications to enhance sustainability, and long-term action planning. A mixed methods approach was used. Organizational (n = 12 sites) ability to demonstrate program effectiveness, align priorities with the organizational mission, and integrate the program within the existing infrastructure were strengths related to sustainability. Sites were more optimistic about program sustainability when they had less reliance on internal financial, but more reliance on internal human resources to run the program post-funding. The study resulted in a number of tools that can help community organizations plan for sustainability of physical activity programs
Notes: DA - 20130927
IS - 1869-6716 (Print)
LA - eng
PT - Journal Article
	27. 	Fanning J, Awick EA, Wojcicki TR et al. Effects of a DVD-Delivered Exercise Intervention on Maintenance of Physical Activity in Older Adults. J.Phys.Act.Health 2015.
Ref ID: 18
Reprint: Not in File
Abstract: BACKGROUND: Previous research supports the efficacy of a 6-month DVD-delivered program for enhancing physical activity (PA) in older adults. In the present study, we examined the degree to which intervention-related increases in PA were maintained after a 6 month, no-contact follow up. METHODS: Follow-up assessments of PA via accelerometry and the Godin Leisure-Time Exercise Questionnaire (GLTEQ) were collected in a sample of older adults (N = 238). Repeated measures analyses of variance were conducted to examine changes in PA over the course of the follow-up period. RESULTS: For accelerometer measured PA, there was a significant time x treatment x age group interaction, F(1,203)=11.319, p = .001, eta2 = .053, such that younger (</=70 years) intervention participants maintained high levels of PA across the follow-up period, while PA in older intervention and young control participants declined significantly. Rates of PA in older control participants remained low over the course of the follow-up period. Analyses of GLTEQ scores revealed similar, though less significant patterns. CONCLUSIONS: DVD-based exercise programs may be effective for maintaining PA in younger members of the older adult population, however there remains a need to develop better strategies for promoting PA maintenance in older individuals when using home-based designs
Notes: DA - 20151123
IS - 1543-5474 (Electronic)
IS - 1543-3080 (Linking)
LA - ENG
PT - JOURNAL ARTICLE
	28. 	Ferry M, Coley N, Andrieu S et al. How to design nutritional intervention trials to slow cognitive decline in apparently healthy populations and apply for efficacy claims: a statement from the International Academy on Nutrition and Aging Task Force. J.Nutr Health Aging 2013;17:619-23.
Ref ID: 70
Keywords: Academies and Institutes/Advisory Committees/Cognition/Cognition Disorders/prevention & control/Dementia/Diet/Health Services Needs and Demand/Humans/Research Design/Treatment Outcome
Reprint: Not in File
Abstract: Interventions are crucial as they offer simple and inexpensive public health solutions that will be useful over the long term use. A Task Force on designing trials of nutritional interventions to slow cognitive decline in older adults was held in Toulouse in September 2012. The aim of the Task Force was to bring together leading experts from academia, the food industry and regulatory agencies to determine the best trial designs that would enable us to reach our goal of maintaining or improving cognitive function in apparently healthy aging people. An associated challenge for this Task Force was to determine the type of trials required by the Public Food Agencies for assessing the impact of nutritional compounds in comparison to well established requirements for drug trials. Although the required quality of the study design, rationale and statistical analysis remains the same, the studies designed to show reduction of cognitive decline require a long duration and the objectives of this task force was to determine best design for these trials. Two specific needs were identified to support trials of nutritional interventions: 1- Risk- reduction strategies are needed to tackle the growing burden of cognitive decline that may lead to dementia, 2- Innovative study designs are needed to improve the quality of these studies
Notes: DA - 20130812
IS - 1760-4788 (Electronic)
IS - 1279-7707 (Linking)
LA - eng
PT - Journal Article
PT - Review
SB - IM
	29. 	Figueira HA, Figueira AA, Cader SA et al. Effects of a physical activity governmental health programme on the quality of life of elderly people. Scand.J.Public Health 2012;40:418-22.
Ref ID: 93
Keywords: Aged/Brazil/Female/Government Programs/Health Promotion/methods/Humans/Male/Middle Aged/Motor Activity/Program Evaluation/Quality of Life/Sedentary Lifestyle/Surveys and Questionnaires
Reprint: Not in File
Abstract: BACKGROUND: The population ageing occurring worldwide resulted in multiple researches on sedentary ageing and quality of life. PURPOSE: To verify the effects of a physical activity programme on the quality of life (QOL) of elderly individuals served by a governmental health programme. DESIGN: Descriptive inquiry research. METHODS: Randomly distributing 70 elderly individuals in a control group (n=35; mean+/-SD 69.80+/-8.05 years) and an experimental group (n=35; 68.66+/-5.93 years) plus QOL evaluation via WHOQOL-Old. RESULTS: The experimental group showed significant best results on the post-test by repeated-measures ANOVA on sensorial functioning (Delta%=0.022%, p=0. 0001), social participation (Delta%=0.012%, p=0.013), perceptions of death and dying (Delta%=0.04%, p=0.009), intimacy (Delta%=0.059%, p=0.05), and total score (Delta%=0.001, p=0.000). CONCLUSIONS: Sensorial functioning, social participation, perceptions of death and dying, and intimacy play an important role in the positive relationship between physical activity and QOL
Notes: DA - 20120817
IS - 1651-1905 (Electronic)
IS - 1403-4948 (Linking)
LA - eng
PT - Journal Article
PT - Randomized Controlled Trial
SB - IM
	30. 	Fisher SG. Community-based nutrition programs and services are needed to improve the health of older adults. J.Am.Diet.Assoc. 2007;107:272-3.
Ref ID: 173
Keywords: Activities of Daily Living/Aged/Aged,80 and over/Aging/physiology/Chronic Disease/Counseling/Diet/Dietetics/organization & administration/standards/Female/Geriatric Assessment/Health Promotion/Health Services for the Aged/Health Status/Humans/Male/Nutrition Assessment/Nutrition Policy/Nutritional Requirements
Reprint: Not in File
Notes: DA - 20070129
IS - 0002-8223 (Print)
IS - 0002-8223 (Linking)
LA - eng
PT - Comment
PT - Journal Article
SB - AIM
SB - IM
	31. 	Floegel TA, Giacobbi PR, Jr., Dzierzewski JM et al. Intervention markers of physical activity maintenance in older adults. Am.J.Health Behav. 2015;39:487-99.
Ref ID: 33
Keywords: Aged/Behavior Therapy/Female/Goals/Humans/Interviews as Topic/Male/Motivation/Motor Activity/Social Support/Surveys and Questionnaires/Treatment Outcome
Reprint: Not in File
Abstract: OBJECTIVES: To identify intervention components that may promote longterm changes of physical activity among older adults in a behavioral theory-based physical activity trial. METHODS: Participants (N = 24; aged 65 +/- 8.79 years) shared perceptions of intervention components at the end of the intervention and physical activity was assessed at 18 months. Mixed-methods analyses using a pragmatic content analysis of interview data were conducted. RESULTS: Active study participants (25%) cited more specific goals/actions to achieve goals and more social support from family/friends, and had significantly higher self-determined motivation mean scores at 18 months than insufficiently active study participants (75%). CONCLUSIONS: Specific goal-setting behaviors and social support from family/friends may be key elements of physical activity maintenance in older adults
Notes: DA - 20150528
IS - 1945-7359 (Electronic)
IS - 1087-3244 (Linking)
LA - eng
PT - Journal Article
PT - Research Support, N.I.H., Extramural
PT - Research Support, Non-U.S. Gov't
SB - IM
	32. 	Foy CG, Vitolins MZ, Case LD et al. Incorporating prosocial behavior to promote physical activity in older adults: rationale and design of the Program for Active Aging and Community Engagement (PACE). Contemp.Clin.Trials 2013;36:284-97.
Ref ID: 71
Keywords: Aged/Aging/physiology/Cognitive Therapy/methods/Consumer Participation/psychology/Disability Evaluation/Exercise/Female/Geriatric Assessment/Health Promotion/organization & administration/Health Status/Humans/Male/Middle Aged/Motivation/Quality of Health Care/Social Behavior/Volunteers
Reprint: Not in File
Abstract: Despite the benefits of regular physical activity among older adults, physical activity rates are low in this population. The Program for Active Aging and Community Engagement (PACE) is an ongoing randomized controlled trial designed to compare the effects of two interventions on physical activity at 12 months among older adults. A total of 300 men and women aged 55 years or older will be randomized into either a healthy aging (HA) control intervention (n = 150), which is largely based upon educational sessions, or a prosocial behavior physical activity (PBPA) intervention (n = 150), which incorporates structured physical activity sessions, cognitive-behavioral counseling, and opportunities to earn food for donation to a regional food bank based on weekly physical activity and volunteering. The PBPA intervention is delivered at a local YMCA, and a regional grocery store chain donates the food to the food bank. Data will be collected at baseline, 3, 6, and 12 months. The primary outcome is physical activity as assessed by the Community Healthy Activities Model Program for Seniors (CHAMPS) Questionnaire at 12 months. Secondary outcomes include physical function and health-related quality of life. If successful, the PACE study will demonstrate that prosocial behavior and volunteerism may be efficaciously incorporated into interventions and will provide evidence for a novel motivating factor for physical activity
Notes: DA - 20130909
IS - 1559-2030 (Electronic)
IS - 1551-7144 (Linking)
LA - eng
PT - Journal Article
PT - Randomized Controlled Trial
PT - Research Support, N.I.H., Extramural
SB - IM
	33. 	Gellert P, Ziegelmann JP, Krupka S, Knoll N, Schwarzer R. An age-tailored intervention sustains physical activity changes in older adults: a randomized controlled trial. Int.J.Behav.Med. 2014;21:519-28.
Ref ID: 72
Keywords: Aged/Aged,80 and over/Exercise/physiology/psychology/Female/Follow-Up Studies/Germany/Health Promotion/Humans/Male/Mental Health/Middle Aged/Physical Endurance/Program Evaluation/Self Efficacy/Surveys and Questionnaires/Treatment Outcome
Reprint: Not in File
Abstract: BACKGROUND: A randomized controlled trial compared an age-tailored intervention to increase physical activity levels in older adults to an age-neutral intervention. PURPOSE: Both interventions communicated activity planning strategies and messages to improve self-efficacy. On top of this, the age-tailored intervention also included two lifespan components that targeted present orientation and emotional focus, and fostered strategies of selection, optimization, and compensation. METHOD: A total of 386 German older adults (aged 60-95 years) were randomized to receive either the age-tailored intervention (age-specific strategy training and short-term emotional focus) or the age-neutral intervention. Physical activity was measured by questionnaires at baseline (T1) and at 6-month (T2) and 12-month follow-ups (T3). Latent true change modeling was applied by creating latent change scores (T2 - T1 and T3 - T2). RESULTS: After controlling for gender, age, and physical and mental health, allocation to the age-tailored intervention predicted a latent physical activity difference at T3 - T2, but not at T2 - T1. CONCLUSION: Compared to the age-neutral intervention, the age-tailored intervention led to superior maintenance of physical activity within these older adults
Notes: DA - 20140505
IS - 1532-7558 (Electronic)
IS - 1070-5503 (Linking)
LA - eng
PT - Journal Article
PT - Randomized Controlled Trial
PT - Research Support, Non-U.S. Gov't
SB - IM
	34. 	Gergerich E, Shobe M, Christy K. Sustaining Our Nation's Seniors through Federal Food and Nutrition Programs. J.Nutr Gerontol.Geriatr. 2015;34:273-91.
Ref ID: 26
Keywords: Adult/Aged/Aged,80 and over/Child/Crops,Agricultural/Diet/Financing,Government/Food Assistance/economics/legislation & jurisprudence/Food Supply/Fruit/Health Policy/Humans/Independent Living/Interpersonal Relations/Middle Aged/Nutritional Physiological Phenomena/Nutritive Value/Poverty/Program Evaluation/United States/United States Department of Agriculture/Vegetables
Reprint: Not in File
Abstract: Food insecurity is a pressing issue in the United States where one in six people suffer from hunger. The older adult population faces unique challenges to receiving adequate nutrition. The federal government currently employs four food and nutrition programs that target the senior population in an effort to address their specific needs. These are the Congregate Meals and Home Delivered Meals Programs (provided through the Older Americans Act), and the Senior Farmers' Market Nutrition Program and Child and Adult Care Food Program (provided by the United States Department of Agriculture). As the older adult population continues to grow, it will be important to evaluate and improve these programs and the social policies related to them. This manuscript describes each policy in depth, considers economic and political elements that have shaped each policy, describes the level of program success, and offers suggestions for future research and program development
Notes: DA - 20150813
IS - 2155-1200 (Electronic)
LA - eng
PT - Journal Article
PT - Review
SB - IM
SB - N
	35. 	Gothe NP, Wojcicki TR, Olson EA et al. Physical activity levels and patterns in older adults: the influence of a DVD-based exercise program. J.Behav.Med. 2015;38:91-7.
Ref ID: 48
Keywords: Accelerometry/Aged/Exercise Therapy/methods/Female/Health Promotion/Humans/Male/Motor Activity/Videodisc Recording
Reprint: Not in File
Abstract: The use of multimedia to influence health behaviors offers unique advantages over more traditional center-based programs, however, little is known about the effectiveness of such approaches in improving physical activity levels over time. The purpose of this study was to examine the efficacy of a progressive and age-appropriate, DVD-delivered exercise program in promoting physical activity levels among older adult cohorts. Community dwelling older adults (N = 307, Mean age = 71 years) were randomized to one of two groups: a 6-month home-based DVD-delivered exercise (i.e., FlexToBa) intervention group or a healthy aging DVD control group. Physical activity was assessed objectively using a standard 7-day accelerometer wear period and subjectively using the Godin Leisure Time Exercise Questionnaire, at baseline and follow-up. Analysis of covariances indicated a statistically significant treatment effect for subjectively [F(1,250) = 8.42, P = .004, eta(2) = .03] and objectively [F(1,240) = 3.77, P = .05, eta(2) = .02] measured physical activity. The older cohort (>70) in the FlexToBa condition further had significantly larger improvements in physical activity levels compared to their younger counterparts. From a public health perspective, media-delivered interventions such as the FlexToBa program might prove to be cost-effective, have a broader reach and at the same time be effective in improving physical activity levels in older adults
Notes: DA - 20150120
IS - 1573-3521 (Electronic)
IS - 0160-7715 (Linking)
LA - eng
PT - Journal Article
PT - Randomized Controlled Trial
PT - Research Support, N.I.H., Extramural
SB - IM
	36. 	Groessl EJ, Kaplan RM, Blair SN et al. A cost analysis of a physical activity intervention for older adults. J.Phys.Act.Health 2009;6:767-74.
Ref ID: 139
Keywords: Activities of Daily Living/Aged/Aged,80 and over/Cost-Benefit Analysis/Counseling/economics/methods/Exercise/physiology/Exercise Therapy/Female/Health Behavior/Health Education/Humans/Male/Pilot Projects/Walking
Reprint: Not in File
Abstract: We examined the costs of a physical activity (PA) and an educational comparison intervention. 424 older adults at risk for mobility disability were randomly assigned to either condition. The PA program consisted of center-based exercise sessions 3x weekly for 8 weeks, 2x weekly for weeks 9 to 24 and weekly behavioral counseling for 10 weeks. Optional sessions were offered during maintenance weeks (25-52). The comparison intervention consisted of weekly education meetings for 24 weeks, and then monthly for 6 months. Cost analyses were conducted from the "payer's" perspective, with a 1-year time horizon. Intervention costs were estimated by tracking personnel activities and materials used for each intervention and multiplying by national unit cost averages. The average cost/participant was $1134 and $175 for the PA and the comparison interventions, respectively. A preliminary cost/effectiveness analysis gauged the cost/disability avoided to be $28,206. Costs for this PA program for older adults are comparable to those of other PA interventions. The results are preliminary and a longer study is required to fully assess the costs and health benefits of these interventions
Notes: DA - 20100127
IS - 1543-3080 (Print)
IS - 1543-3080 (Linking)
LA - eng
PT - Journal Article
PT - Randomized Controlled Trial
PT - Research Support, N.I.H., Extramural
PT - Research Support, N.I.H., Intramural
PT - Research Support, U.S. Gov't, Non-P.H.S
SB - IM
	37. 	Groessl EJ, Kaplan RM, Castro Sweet CM et al. Cost-effectiveness of the LIFE Physical Activity Intervention for Older Adults at Increased Risk for Mobility Disability. J.Gerontol.A Biol.Sci.Med.Sci. 2016;71:656-62.
Ref ID: 9
Reprint: Not in File
Abstract: BACKGROUND: Losing the ability to walk safely and independently is a major concern for many older adults. The Lifestyle Interventions and Independence for Elders study recently demonstrated that a physical activity (PA) intervention can delay the onset of major mobility disability. Our objective is to examine the resources required to deliver the PA intervention and calculate the incremental cost-effectiveness compared with a health education intervention. METHODS: The Lifestyle Interventions and Independence for Elders study enrolled 1,635 older adults at risk for mobility disability. They were recruited at eight field centers and randomly assigned to either PA or health education. The PA program consisted of 50-minute center-based exercise 2x weekly, augmented with home-based activity to achieve a goal of 150min/wk of PA. Health education consisted of weekly workshops for 26 weeks, and monthly sessions thereafter. Analyses were conducted from a health system perspective, with a 2.6-year time horizon. RESULTS: The average cost per participant over 2.6 years was US$3,302 and US$1,001 for the PA and health education interventions, respectively. PA participants accrued 0.047 per person more Quality-Adjusted Life-Years (QALYs) than health education participants. PA interventions costs were slightly higher than other recent PA interventions. The incremental cost-effectiveness ratios were US$42,376/major mobility disability prevented and US$49,167/QALY. Sensitivity analyses indicated that results were relatively robust to varied assumptions. CONCLUSIONS: The PA intervention costs and QALYs gained are comparable to those found in other studies. The ICERS are less than many commonly recommended medical treatments. Implementing the intervention in non-research settings may reduce costs further
Notes: DA - 20160413
IS - 1758-535X (Electronic)
IS - 1079-5006 (Linking)
LA - eng
PT - Journal Article
SB - AIM
SB - IM
	38. 	Hall KS, Crowley GM, Bosworth HB, Howard TA, Morey MC. Individual progress toward self-selected goals among older adults enrolled in a physical activity counseling intervention. J.Aging Phys.Act. 2010;18:439-50.
Ref ID: 129
Keywords: Aged/Aged,80 and over/Counseling/Exercise/psychology/Goals/Health Status/Humans/Longitudinal Studies/Male/Models,Theoretical/Veterans/Virginia/Walking
Reprint: Not in File
Abstract: The purpose of this study was to examine what happens to goals over the course of a physical activity counseling trial in older veterans. At baseline, participants (N = 313) identified 1 health-related goal and 1 walking goal for their participation in the study and rated where they perceived themselves to be relative to that goal at the current time. They rated their current status on these same goals again at 6 and 12 mo. Growth-curve analyses were used to examine longitudinal change in perceived goal status. Although both the intervention and control groups demonstrated improvement in their perceived proximity to their health-related and walking goals (L = 1.19, p < .001), the rates of change were significantly greater in the intervention group (beta = -.30, p < .05). Our results demonstrate that this physical activity counseling intervention had a positive impact on self-selected goals over the course of the intervention
Notes: DA - 20101019
IS - 1063-8652 (Print)
IS - 1063-8652 (Linking)
LA - eng
PT - Journal Article
PT - Research Support, N.I.H., Extramural
PT - Research Support, Non-U.S. Gov't
SB - IM
	39. 	Hall KS, Sloane R, Pieper CF et al. Long-term changes in physical activity following a one-year home-based physical activity counseling program in older adults with multiple morbidities. J.Aging Res. 2010;2011:308407.
Ref ID: 125
Reprint: Not in File
Abstract: This study assessed the sustained effect of a physical activity (PA) counseling intervention on PA one year after intervention, predictors of sustained PA participation, and three classes of post-intervention PA trajectories (improvers, maintainers, and decliners) in 238 older Veterans. Declines in minutes of PA from 12 to 24 months were observed for both the treatment and control arms of the study. PA at 12 months was the strongest predictor of post-intervention changes in PA. To our surprise, those who took up the intervention and increased PA levels the most, had significant declines in post-intervention PA. Analysis of the three post-intervention PA trajectories demonstrated that the maintenance group actually reflected a group of nonresponders to the intervention who had more comorbidities, lower self-efficacy, and worse physical function than the improvers or decliners. Results suggest that behavioral counseling/support must be ongoing to promote maintenance. Strategies to promote PA appropriately to subgroups of individuals are needed
Notes: DA - 20110114
IS - 2090-2212 (Electronic)
IS - 2090-2204 (Linking)
LA - eng
PT - Journal Article
	40. 	Hammerback K, Felias-Christensen G, Phelan EA. Evaluation of a telephone-based physical activity promotion program for disadvantaged older adults. Prev.Chronic.Dis. 2012;9:E62.
Ref ID: 104
Keywords: Aged/Aging/Health Promotion/methods/Humans/Motor Activity/Telephone/Vulnerable Populations/psychology
Reprint: Not in File
Abstract: BACKGROUND: Lack of adequate physical activity among older adults has been widely documented. Although interventions aimed at increasing physical activity that are based on behavioral strategies and theories have been shown to increase activity levels among older adults, little is known about responses to these interventions in different population segments. COMMUNITY CONTEXT: The Physical Activity for a Lifetime of Success (PALS) program attempted to translate a telephone-based, motivational support program for physical activity, Active Choices, for use by a low-income, ethnically diverse population of older adults living in southeast Seattle. This article describes the evaluation of PALS at the end of the 5-year program. METHODS: Evaluation data included a data set of participant physical activity assessments; internal study documents; and interviews with key PALS stakeholders, participants, volunteers, and people eligible for PALS who declined to enroll when invited. OUTCOME: PALS demonstrated improved physical activity levels among the sedentary older adults who participated in the program, but the PALS model did not appeal widely to a diverse, low-income target population. Extensive recruitment efforts resulted in a low number of participants, and attempts to recruit peer volunteers were largely unsuccessful. INTERPRETATION: Considering the resources required to engage both participants and volunteers, PALS does not appear to be a sustainable model for delivering support for physical activity to community-dwelling minority and low-income older adults
Notes: DA - 20120224
IS - 1545-1151 (Electronic)
IS - 1545-1151 (Linking)
LA - eng
PT - Evaluation Studies
PT - Journal Article
PT - Research Support, U.S. Gov't, P.H.S
SB - IM
	41. 	Harden SM, Fanning JT, Motl RW, McAuley E, Estabrooks PA. Determining the reach of a home-based physical activity program for older adults within the context of a randomized controlled trial. Health Educ.Res. 2014;29:861-9.
Ref ID: 45
Reprint: Not in File
Abstract: Determining the reach of physical activity (PA) programs is challenging due to inconsistent reporting across studies. The purpose of this study was to document multiple indicators of program reach for a 6-month, Digital Versatile Disc (DVD)-delivered home-based PA program. Radio, newspaper and direct mailing advertisements were tracked to determine costs as well as the number and representativeness of older adults exposed and responding to recruitment. It was estimated that all older adults in the recruitment area (n = 105 515) may have been exposed to at least one of the recruitment strategies--563 responded and 383 were screened as eligible. Of those that enrolled (n = 307), the DVD reached between 81% and 97% of the participants over each month within the 6 month period. Newspaper advertisements were most effective (n = 222) at a cost of $78 per participant enrolled. CONCLUSION: Using multiple indicators of reach supports the accurate calculation and generalizability of recruiting older adults into PA programs
Notes: DA - 20140918
IS - 1465-3648 (Electronic)
IS - 0268-1153 (Linking)
LA - eng
PT - Journal Article
PT - Research Support, N.I.H., Extramural
SB - T
	42. 	Hernandes NA, Probst VS, Da SR, Jr., Januario RS, Pitta F, Teixeira DC. Physical activity in daily life in physically independent elderly participating in community-based exercise program. Braz.J.Phys.Ther. 2013;17:57-63.
Ref ID: 88
Keywords: Activities of Daily Living/Aged/Cross-Sectional Studies/Exercise/Female/Humans/Male/Middle Aged/Motor Activity
Reprint: Not in File
Abstract: BACKGROUND: It is unclear whether participation in exercise programs specifically developed for elderly translates into a more active lifestyle. OBJECTIVES: To compare the objectively measured level of physical activity in daily life (PADL) between physically independent elderly who participate or do not participate in community-based exercise programs; and to evaluate which factors are associated with the higher level of PADL in these subjects. METHOD: 134 elderly participants in community-based exercise programs (PG) and 104 non-participants (NPG) had their level of PADL measured using pedometers during 7 days. OTHER MEASUREMENTS: 6-minute walking test (6MWT), incremental shuttle walking test (ISWT), muscle strength, flexibility and balance. RESULTS: The PG had higher 1-week mean daily step count than NPG (8314 [IQR 5971-10060] vs. 6250 [IQR 4346-8207] steps/day, p<0.0001), as well as higher step count in any day of the week. There was a higher proportion of physically active subjects (>8000 steps/day) in PG than in NPG (37% vs. 16%, respectively; p<0.001), as well as the proportion of sedentary subjects (<5000 steps/day) (14% vs. 33%, respectively; p<0.001). Participation in exercise programs, 6MWT and ISWT explained a higher daily steps count (model r(2)=0.56, p<0.0001). CONCLUSIONS: In physically independent elderly, a higher level of physical activity in daily life occurs in those who participate in community-based exercise programs, regardless of the weekday and including non-program days. Participation of elderly in community-based exercise programs should be more systematically available and encouraged due to its close link to higher activity levels and better exercise capacity
Notes: DA - 20130329
IS - 1809-9246 (Electronic)
IS - 1413-3555 (Linking)
LA - eng
PT - Journal Article
SB - IM
	43. 	Hildebrand M, Neufeld P. Recruiting older adults into a physical activity promotion program: Active Living Every Day offered in a naturally occurring retirement community. Gerontologist 2009;49:702-10.
Ref ID: 147
Keywords: Aged/Aged,80 and over/Female/Health Promotion/methods/Housing for the Elderly/Humans/Male/Missouri/Motivation/Motor Activity/Physical Fitness
Reprint: Not in File
Abstract: PURPOSE: This article explores recruitment strategies based on the transtheoretical model (TTM) with older adults living in a naturally occurring retirement community (NORC) to encourage enrollment in a physical activity promotion program, Active Living Every Day (ALED). Reasons for participation or nonparticipation are identified. DESIGN AND METHODS: Recruitment strategies were designed to move older adults through the TTM stages of change to enroll in ALED and were built on meetings and resources established by St. Louis NORC's supportive service program. NORC residents (25 ALED participants and 25 nonparticipants) were interviewed about reasons for enrollment or nonenrollment. RESULTS: A significant difference was found between the two groups on their responses to a physical activity stage-of-change question, although no significant differences were found in their demographics, social resources, and mood/depression. ALED participants' motivation to enroll primarily came from TTM proactive recruitment methods (88%) and less (12%) from reactive methods. Themes for ALED participants' choices to enroll included motivation to exercise, physical activity ideas from peers, social engagement, and trust in sponsoring organizations' staff and programs. Analysis of interview data identified that scheduling and cost were primary reasons for nonenrollment in ALED. IMPLICATIONS: Using theoretically based recruitment methods for older adults and a neighborhood approach through organizations such as a NORC may result in greater numbers of older adults participating in health promotion programs
Notes: DA - 20090914
IS - 1758-5341 (Electronic)
IS - 0016-9013 (Linking)
LA - eng
PT - Journal Article
SB - IM
	44. 	Hinrichs T, Brach M. The general practitioner's role in promoting physical activity to older adults: a review based on program theory. Curr.Aging Sci. 2012;5:41-50.
Ref ID: 117
Keywords: Aged/Aged,80 and over/Counseling/General Practitioners/Germany/Health Promotion/Humans/Motor Activity/Physician's Role/Primary Health Care/Systems Theory
Reprint: Not in File
Abstract: Positive influences of physical activity both on many chronic diseases and on preservation of mobility are well documented. But chronically ill or mobility restricted elderly living in their own homes are difficult to reach for interventions. The general practitioner's (GP) surgery offers one of the few opportunities to give advice for physical activity to those people. We used program theory to sound out knowledge on GP-centered physical activity counseling. The "conceptual theory" (evidence for training effects in old age) and the "implementation theory" (unique position of the GP) were reviewed narratively. The "action theory" (effects of GP counseling) was reviewed systematically. According to program theory, appropriate MeSH (Medical subject headings) concepts were Aged OR Aged, 80 and over (Target group), Physicians, Family OR Primary Health Care (Implementation/Setting), Counseling OR Patient Education as Topic OR Disease Management OR Health promotion (Intervention), Exercise OR Motor Activity OR Physical Fitness OR Sports (Determinants). The resulting six review papers (Pubmed, 2000-2009) were presented using the STARLITE mnemonic. Authors agree, that the GP plays a central role in the promotion of physical activity to elderly people, but there is conflicting evidence concerning counseling effectiveness. Utilizing behavioral change strategies and the collaboration between GPs and specialised professions are recommended and currently under research
Notes: DA - 20120322
IS - 1874-6128 (Electronic)
LA - eng
PT - Journal Article
PT - Review
SB - IM
	45. 	Homenko DR, Morin PC, Eimicke JP, Teresi JA, Weinstock RS. Food insecurity and food choices in rural older adults with diabetes receiving nutrition education via telemedicine. J.Nutr Educ.Behav. 2010;42:404-9.
Ref ID: 128
Keywords: African Americans/Aged/Aged,80 and over/Body Mass Index/Choice Behavior/Diabetes Mellitus/Educational Status/Elder Nutritional Physiological Phenomena/Female/Follow-Up Studies/Food Habits/Food Supply/statistics & numerical data/Humans/Male/Motor Activity/Obesity/metabolism/Rural Population/Telemedicine/methods
Reprint: Not in File
Abstract: OBJECTIVE: To evaluate differences between rural older adults with diabetes reporting the presence or absence of food insecurity with respect to meal planning, preparation, shopping, obesity, and glycemic control after receiving nutrition counseling through telemedicine. METHODS: Food insecurity data were obtained by telephone survey (n=74). Group differences for continuous variables were measured by t tests; categorical variables by Pearson chi-square tests. RESULTS: Participants reporting mild food insecurity (23%) had higher body mass index (35.5+/-7.1 kg/m2 vs 30.5+/-6.0 kg/m2, P=.01) and lower household incomes (P=.03) and were more likely to consider cost of ingredients in food preparation compared to food-secure participants (P=.03). Most purchased fresh produce (97%) and considered the dietitian's advice when purchasing food. Both groups report similar adherence to dietitians' advice and had similar glycemic control. CONCLUSIONS AND IMPLICATIONS: Strategies to address higher levels of obesity associated with food insecurity are needed
Notes: DA - 20101112
IS - 1878-2620 (Electronic)
IS - 1499-4046 (Linking)
LA - eng
PT - Journal Article
PT - Research Support, U.S. Gov't, P.H.S
SB - IM
	46. 	Hooker SP, Seavey W, Weidmer CE et al. The California active aging community grant program: translating science into practice to promote physical activity in older adults. Ann.Behav.Med. 2005;29:155-65.
Ref ID: 188
Keywords: Aged/Aged,80 and over/Aging/Community Health Services/Energy Metabolism/Exercise Therapy/Female/Health Promotion/Health Status/Humans/Male/Middle Aged/Professional-Patient Relations/Quality of Life/Social Support/Treatment Outcome
Reprint: Not in File
Abstract: BACKGROUND: Attempts to study the translation of evidence-based physical activity interventions in community settings are scarce. PURPOSE: This project was an investigation of whether 13 diverse local lead agencies could effectively implement a choice-based, telephone-assisted physical activity promotion program for older adults based on intervention models proven efficacious in research settings. METHODS: At baseline, participants developed their own physical activity programs through an individualized planning session based on preference, health status, readiness to change, and available community resources. Thereafter, participants received regular telephone calls over a 1-year period from a trained staff member or volunteer support buddy. Additional program components consisted of health education workshops, newsletters, and group-based physical activities. Self-report data on caloric expenditure due to all and moderate or greater intensity physical activities were collected from 447 participants (M age = 68 +/- 8.6 years). RESULTS: A significant increase (p < or = .0001) from baseline to midintervention and intervention endpoint was observed for total weekly caloric expenditure (Mdn change = 644-707 kcal/week) and moderate or greater weekly caloric expenditure (Mdn change = 149-265 kcal/week), as well as for weekly physical activity duration and frequency. These changes were observed in participants across all sites. CONCLUSIONS: The increases in weekly caloric expenditure were commensurate with findings from several previous randomized clinical trials. The utilization of community agency staff and volunteers receiving basic training to implement essential program components proved feasible. Very favorable levels of program satisfaction expressed by community staff, volunteer support buddies, and participants, combined with the significant increases in physical activity, warrant further dissemination of the intervention model
Notes: DA - 20050610
IS - 0883-6612 (Print)
IS - 0883-6612 (Linking)
LA - eng
PT - Clinical Trial
PT - Journal Article
PT - Research Support, Non-U.S. Gov't
PT - Research Support, U.S. Gov't, Non-P.H.S
SB - IM
	47. 	Hughes SL, Seymour RB, Campbell RT, Whitelaw N, Bazzarre T. Best-practice physical activity programs for older adults: findings from the national impact study. Am.J.Public Health 2009;99:362-8.
Ref ID: 152
Keywords: Aged/Aged,80 and over/Exercise/Female/Humans/Interviews as Topic/Male/Middle Aged/Muscle Strength/Outcome Assessment (Health Care)/methods/Self Efficacy/United States
Reprint: Not in File
Abstract: OBJECTIVES: We assessed the impact of existing best-practice physical activity programs for older adults on physical activity participation and health-related outcomes. METHODS: We used a multisite, randomized trial with 544 older adults (mean age 66 years) and measures at baseline, 5, and 10 months to test the impact of a multiple-component physical activity program compared with results for a control group that did not participate in such a program. RESULTS: For adults who participated in a multiple-component physical activity program, we found statistically significant benefits at 5 and 10 months with regard to self-efficacy for exercise adherence over time (P < .001), adherence in the face of barriers (P = .01), increased upper- and lower-body strength (P = .02, P = .01), and exercise participation (P = .01). CONCLUSIONS: Best-practice community-based physical activity programs can measurably improve aspects of functioning that are risk factors for disability among older adults. US public policy should encourage these inexpensive health promotion programs
Notes: DA - 20090114
IS - 1541-0048 (Electronic)
IS - 0090-0036 (Linking)
LA - eng
PT - Journal Article
PT - Multicenter Study
PT - Randomized Controlled Trial
PT - Research Support, Non-U.S. Gov't
SB - AIM
SB - IM
	48. 	Hughes SL, Williams B, Molina LC et al. Characteristics of physical activity programs for older adults: results of a multisite survey. Gerontologist 2005;45:667-75.
Ref ID: 186
Keywords: Aged/Aged,80 and over/Exercise/Fitness Centers/utilization/Health Care Surveys/methods/Health Promotion/Health Services Needs and Demand/Health Services for the Aged/Humans/Motor Activity/United States
Reprint: Not in File
Abstract: PURPOSE: Although increased participation in physical activity by older adults is a major public health goal, little is known about the supply and use of physical activity programs in the United States. DESIGN AND METHODS: Seven academic centers in diverse geographic areas surveyed physical activity programs for older adults. Five sites conducted surveys by mail with telephone follow-up, and two administered surveys primarily by telephone. Reported program attendance rates were compared with local census data to assess unmet needs. RESULTS: Of the 2,110 targeted facilities, 77% responded. Aerobic programs were offered by 73%, flexibility by 47%, and strength training by 26%. Commercial gyms or YMCAs, senior centers, park or recreation centers, and senior-housing facilities offered 90% of available programs. The 2000 Census enumerated 1,123,401 total older adults across the seven sites. Facilities reported 69,634 individuals as current weekly program participants, equaling 6% of the sites' total older-adult population. This percentage varied from 3% in Pittsburgh to 28% in Colorado. IMPLICATIONS: Based on conservative estimates of demand, the number of physical activity programs would have to increase substantially (by 78%) to meet the needs of older adults. The data also indicate the need to develop more strength-training programs and to engage a higher percentage of older adults in these programs. There is a clear need to stimulate demand for programs through health promotion
Notes: DA - 20051003
IS - 0016-9013 (Print)
IS - 0016-9013 (Linking)
LA - eng
PT - Journal Article
PT - Research Support, N.I.H., Extramural
PT - Research Support, Non-U.S. Gov't
PT - Research Support, U.S. Gov't, P.H.S
SB - IM
	49. 	Hyland RM, Wood CE, Adamson AJ et al. Peer educators' perceptions of training for and implementing a community-based nutrition intervention for older adults. J.Nutr Elder. 2006;25:147-71.
Ref ID: 169
Keywords: Aged/Aged,80 and over/Attitude to Health/Cooking/methods/Diet/England/Female/Food Handling/Health Education/Humans/Interviews as Topic/Male/Motivation/Nutritional Physiological Phenomena/physiology/Peer Group/Perception/Personal Satisfaction/Program Evaluation/Residence Characteristics
Reprint: Not in File
Abstract: This study aimed to evaluate the use of peer educators in nutrition interventions with older people. A sample of 22 people aged 60+ were recruited and trained using an accredited course for Community Nutrition Assistants which included basic nutrition and group skills. They were paid to work as peer educators in a 20-week food club intervention which ran in 13 sheltered accommodation schemes for older people in northeast England. Clubs ran for 2 hours each week and included food preparation, food tasting and sharing information and ideas about food and health. This paper reports key findings from qualitative interviews with peer educators on their perspectives on their motivation to participate, their training and their implementation of the food club intervention. It discusses some of the issues involved in the training and use of peer educators and presents lessons learned, particularly the need to target training, according to prior experience and skills
Notes: DA - 20071122
IS - 0163-9366 (Print)
IS - 0163-9366 (Linking)
LA - eng
PT - Evaluation Studies
PT - Journal Article
PT - Research Support, Non-U.S. Gov't
SB - IM
	50. 	Irvine AB, Gelatt VA, Seeley JR, Macfarlane P, Gau JM. Web-based intervention to promote physical activity by sedentary older adults: randomized controlled trial. J.Med.Internet.Res. 2013;15:e19.
Ref ID: 81
Keywords: Aged/Exercise/psychology/Female/Health Behavior/Health Promotion/methods/statistics & numerical data/Humans/Internet/Male/Middle Aged/Motor Activity/Multivariate Analysis/Muscle Strength/Muscle Stretching Exercises/Outcome Assessment (Health Care)/Physical Endurance/Postural Balance/Sedentary Lifestyle/Telemedicine
Reprint: Not in File
Abstract: BACKGROUND: Physical activity (PA) for older adults has well-documented physical and cognitive benefits, but most seniors do not meet recommended guidelines for PA, and interventions are lacking. OBJECTIVES: This study evaluated the efficacy of a 12-week Internet intervention to help sedentary older adults over 55 years of age adopt and maintain an exercise regimen. METHODS: A total of 368 sedentary men and women (M=60.3; SD 4.9) were recruited, screened, and assessed online. They were randomized into treatment and control groups and assessed at pretest, at 12 weeks, and at 6 months. After treatment group participants rated their fitness level, activity goals, and barriers to exercise, the Internet intervention program helped them select exercise activities in the areas of endurance, flexibility, strengthening, and balance enhancement. They returned to the program weekly for automated video and text support and education, with the option to change or increase their exercise plan. The program also included ongoing problem solving to overcome user-identified barriers to exercise. RESULTS: The multivariate model indicated significant treatment effects at posttest (P=.001; large effect size) and at 6 months (P=.001; medium effect size). At posttest, intervention participation showed significant improvement on 13 of 14 outcome measures compared to the control participants. At 6 months, treatment participants maintained large gains compared to the control participants on all 14 outcome measures. CONCLUSIONS: These results suggest that an online PA program has the potential to positively impact the physical activity of sedentary older adult participants. More research is needed to replicate the study results, which were based on self-report measures. Research is also needed on intervention effects with older populations
Notes: DA - 20130308
IS - 1438-8871 (Electronic)
IS - 1438-8871 (Linking)
LA - eng
PT - Journal Article
PT - Randomized Controlled Trial
PT - Research Support, N.I.H., Extramural
SB - IM
	51. 	Jancey J, Lee A, Howat P, Clarke A, Wang K, Shilton T. Reducing attrition in physical activity programs for older adults. J.Aging Phys.Act. 2007;15:152-65.
Ref ID: 172
Keywords: Age Factors/Aged/Attitude to Health/Australia/Body Mass Index/Exercise/physiology/Female/Health Behavior/Health Promotion/Humans/Leisure Activities/Male/Motor Activity/Program Development/Self Efficacy/Surveys and Questionnaires
Reprint: Not in File
Abstract: This study investigated attrition in a 6-month physical activity intervention for older adults. The program was based on the social-cognitive theory incorporating self-efficacy factors. Two hundred forty-eight insufficiently active 65- to 74-year-olds were recruited from the Australian federal electoral roll. The intervention comprised walking and strength and flexibility exercises and was conducted in 30 local neighborhoods where the participants resided. Characteristics of individuals lost to attrition (n = 86, 35%) were compared with those of program completers (n = 162, 65%). Logistic-regression analysis showed that those lost to attrition came from areas of lower socioeconomic status, were overweight and less physically active, and had lower walking self-efficacy scores and higher loneliness scores. The results suggest that early assessment of these characteristics should be undertaken to identify individuals at risk of attrition, to improve retention, and to avoid potential bias
Notes: DA - 20070608
IS - 1063-8652 (Print)
IS - 1063-8652 (Linking)
LA - eng
PT - Journal Article
SB - IM
	52. 	Jancey JM, Lee AH, Howat PA, Clarke A, Wang K, Shilton T. The effectiveness of a physical activity intervention for seniors. Am.J.Health Promot. 2008;22:318-21.
Ref ID: 159
Keywords: Aged/Body Mass Index/Exercise/Female/Health Promotion/methods/organization & administration/Humans/Male/Prospective Studies/Social Support/Socioeconomic Factors
Reprint: Not in File
Abstract: PURPOSE: To determine whether a tailored, 6-month, neighborhood-based, physical activity intervention for people aged 65 to 74 years could increase their total physical activity levels and to identify factors associated with physical activity times. DESIGN: A longitudinal, prospective, intervention study. SETTING: Perth, Western Australia. SUBJECTS: A total of 573 older adults, recruited from 30 intervention (n = 260) and 30 control (n = 313) neighborhoods. Initial response rates were 74% (260/352) in the intervention group and 82% (313/382) in the control group, which provided the 573 adults for participation in the study. A total of 413 participants (177 and 236 in the intervention and control groups, respectively) completed the program. INTERVENTION: A neighborhood-based physical activity intervention. MEASURES: A self-reported questionnaire administered at three time points. Physical activity levels were measured using the International Physical Activity Questionnaire. Personal and demographic information, including perceived financial struggle and proximity to friends, were collected. ANALYSIS: Descriptive statistics, repeated measures analysis of variance, and generalized estimating equations (GEE). RESULTS: The intervention resulted in a significant increase in total average physical activity times of 2.25 hours per week (p < .001). The GEE analysis confirmed significant increases in physical activity from baseline to midpoint (p = .002) and topostintervention (p = .031). Perception of financial struggle (p = .020) was positively associated with physical activity time spent by participants, whereas having no friends or acquaintances living nearby (p = .037) had a significant negative correlation. The main limitation of this study was the restricted duration of the intervention. CONCLUSION: The program was successful in increasing weekly mean time for physical activity in seniors and in identifying factors that affect their commitment to physical activities
Notes: DA - 20080603
IS - 0890-1171 (Print)
IS - 0890-1171 (Linking)
LA - eng
PT - Journal Article
PT - Randomized Controlled Trial
PT - Research Support, Non-U.S. Gov't
SB - T
	53. 	Johnson GS, McGee BB, Gossett JM et al. Documenting the need for nutrition and health intervention for middle-aged and older adults in the Lower Mississippi Delta region. J.Nutr Elder. 2008;27:83-99.
Ref ID: 155
Keywords: African Americans/statistics & numerical data/Aged/Aging/Body Weight/Cross-Sectional Studies/Diet/methods/standards/Diet Records/Diet Surveys/European Continental Ancestry Group/Female/Geriatric Assessment/Health Status/Humans/Male/Middle Aged/Mississippi/Needs Assessment/Nutrition Assessment/Nutritional Status/ethnology/Rural Population/Socioeconomic Factors
Reprint: Not in File
Abstract: Multiple demographic, health, and environmental factors may influence the overall quality of diets among rural middle-aged and older adults. This project compared the diet quality of participants in Foods of Our Delta Survey (FOODS 2000) who were aged 55 years and older with national data. The data were assessed using 24-hour dietary recall methodology and a modified version of the United States Department of Agriculture Healthy Eating Index (HEI) that excluded the sodium component. The mean total Modified Healthy Eating Index (MHEI) study score was significantly lower than their counterparts from the national survey (61.0 +/- 0.68 vs. 65.6 +/- 3.65, P < 0.0001). Race and educational attainment were associated with higher MHEI scores. This study emphasized a critical need for implementing nutrition and health interventions in rural communities with special attention to subpopulations at risk
Notes: DA - 20081020
IS - 0163-9366 (Print)
IS - 0163-9366 (Linking)
LA - eng
PT - Journal Article
SB - IM
	54. 	Kamp BJ, Wellman NS, Russell C. Position of the American Dietetic Association, American Society for Nutrition, and Society for Nutrition Education: food and nutrition programs for community-residing older adults. J.Nutr Educ.Behav. 2010;42:72-82.
Ref ID: 137
Keywords: Aged/Aged,80 and over/Aging/physiology/Dietary Services/Dietetics/standards/Female/Food Services/Health Status/Humans/Independent Living/Male/Nutrition Policy/Nutritional Physiological Phenomena/Nutritional Requirements/Nutritional Sciences/Nutritional Status/Risk Factors/Societies/Socioeconomic Factors/United States
Reprint: Not in File
Abstract: Given the federal cost-containment policy to rebalance long-term care away from nursing homes to home- and community-based services, it is the position of the American Dietetic Association, the American Society for Nutrition, and the Society for Nutrition Education that all older adults should have access to food and nutrition programs that ensure the availability of safe, adequate food to promote optimal nutritional status. Appropriate food and nutrition programs include adequately funded food assistance and meal programs, nutrition education, screening, assessment, counseling, therapy, monitoring, evaluation, and outcomes documentation to ensure more healthful aging. The growing number of older adults, the health care focus on prevention, and the global economic situation accentuate the fundamental need for these programs. Yet far too often food and nutrition programs are disregarded or taken for granted. Growing older generally increases nutritional risk. Illnesses and chronic diseases; physical, cognitive, and social challenges; racial, ethnic, and linguistic differences; and low socioeconomic status can further complicate a situation. The beneficial effects of nutrition for health promotion, risk reduction, and disease management need emphasis. Although many older adults are enjoying longer and more healthful lives in their own homes, others, especially those with health disparities and poor nutritional status, would benefit from greater access to food and nutrition programs and services. Food and nutrition practitioners can play a major role in promoting universal access and integrating food and nutrition programs and nutrition services into home- and community-based services
Notes: DA - 20100311
IS - 1878-2620 (Electronic)
IS - 1499-4046 (Linking)
LA - eng
PT - Journal Article
SB - IM
	55. 	Katula JA, Church T, Sink KM. Physical Activity vs Health Education for Cognition in Sedentary Older Adults--Reply. JAMA 2016;315:415-6.
Ref ID: 12
Keywords: Dementia/prevention & control/Executive Function/Exercise Therapy/methods/Female/Health Promotion/Humans/Male/Mild Cognitive Impairment/Sedentary Lifestyle
Reprint: Not in File
Notes: DA - 20160127
IS - 1538-3598 (Electronic)
IS - 0098-7484 (Linking)
LA - eng
PT - Comment
PT - Letter
SB - AIM
SB - IM
	56. 	Keller HH, Hedley MR, Wong SS, Vanderkooy P, Tindale J, Norris J. Community organized food and nutrition education: participation, attitudes and nutritional risk in seniors. J.Nutr Health Aging 2006;10:15-20.
Ref ID: 183
Keywords: Aged/Attitude to Health/Community Health Centers/organization & administration/Consumer Participation/Eating/Female/Follow-Up Studies/Food Habits/Geriatric Assessment/Health Education/Health Knowledge,Attitudes,Practice/Health Promotion/methods/Humans/Male/Nutrition Surveys/Nutritional Requirements/Nutritional Sciences/education/Program Evaluation/Risk Assessment/Surveys and Questionnaires
Reprint: Not in File
Abstract: Evergreen Action Nutrition (EAN) is a health promotion program designed to facilitate relatively healthy members of a seniors recreation center to maintain their nutritional health as they age. A main goal of this project was to demonstrate the feasibility and relevance of using the community organization approach to develop a nutrition education program for seniors. METHODS: Using the current membership list, seniors were randomly selected to receive a mailed baseline (n=247) questionnaire. A follow-up survey (n=251) was sent out to randomly selected members three years later to determine participation in EAN and reported behavior change. Although not the same individuals, responses were compared to baseline to determine changes in nutritional risk. Items from the Diet and Health Knowledge survey were compared by EAN participation. RESULTS: The program had a large reach with 162 survey respondents (64.5%) reporting some level of participation and 51% reporting "frequent" participation. Use of informal forms of education predominated (e.g. displays). Significant differences were found between baseline and follow-up for risk attributed to low intake of fruits and vegetables and frequency of eating, with EAN participants having reduced risk of low fruit and vegetable intake. Those participating in formal education (e.g. food workshops) reported more frequent changes in food practices than those participating in informal activities. EAN participants appear to have more healthy nutrition attitudes/beliefs. CONCLUSION: The community organization approach to program planning and delivery leads to the development of diverse and appropriate nutrition education activities for seniors. Informal and formal health promotion activities can be successfully implemented in recreation centers
Notes: DA - 20060202
IS - 1279-7707 (Print)
IS - 1279-7707 (Linking)
LA - eng
PT - Journal Article
SB - IM
	57. 	Keller HH, Hedley M, Hadley T, Wong S, Vanderkooy P. Food workshops, nutrition education, and older adults: a process evaluation. J.Nutr Elder. 2005;24:5-23.
Ref ID: 191
Keywords: Aged/Aged,80 and over/Cooking/Dietary Services/methods/organization & administration/Female/Focus Groups/Food/Humans/Male/Middle Aged/Nutritional Sciences/education/Observer Variation/Program Evaluation
Reprint: Not in File
Abstract: Three years of process evaluation data are presented for Evergreen ActionNutrition food workshops conducted at an older adults' recreation center. Over a three-year period, 475 written evaluations of single and three-session series workshops were completed. Additionally, data were collected from four focus groups of older adult participants (n = 19). Older persons report intention to change behavior based on attendance at a single workshop. Series workshop participants report increased knowledge, confidence in cooking, and motivation to make changes. Focus group participants identified the following as key aspects to success and empowerment of behavior change: relevant information, specifically in the form of recipes; interactive format, including taste-testing; social experience; consistent, high-quality education; and small size of the group. Food workshops or demonstrations can be meaningful nutrition education activities for older adults
Notes: DA - 20050524
IS - 0163-9366 (Print)
IS - 0163-9366 (Linking)
LA - eng
PT - Journal Article
PT - Research Support, Non-U.S. Gov't
SB - IM
	58. 	Keller HH. Meal programs improve nutritional risk: a longitudinal analysis of community-living seniors. J.Am.Diet.Assoc. 2006;106:1042-8.
Ref ID: 177
Keywords: Activities of Daily Living/Aged/Cohort Studies/Female/Follow-Up Studies/Food Services/utilization/Geriatric Assessment/Health Status/Humans/Interviews as Topic/Linear Models/Longitudinal Studies/Male/Mass Screening/Nutrition Assessment/Nutrition Surveys/Nutritional Status/Ontario/Risk Assessment/Social Support/Surveys and Questionnaires
Reprint: Not in File
Abstract: OBJECTIVE: To determine the independent association of meal programs (eg, Meals On Wheels and other meal programs with a social component) and shopping help on seniors' nutritional risk. DESIGN: Cohort design. Baseline data were collected with an in-person interview and subjects were followed up for 18 months via telephone interview. SUBJECTS/SETTING: Cognitively well, vulnerable (ie, required informal or formal supports for activities of daily living) seniors were recruited through community service agencies in southwestern Ontario, Canada. Three hundred sixty-seven seniors participated in baseline interviews and 263 completed data collection at 18-month follow-up; 70% participated in meal programs at baseline. MAIN OUTCOME MEASURES: The 15-item Seniors in the Community: Risk Evaluation for Eating and Nutrition (SCREEN) questionnaire identified nutritional risk at 18 months. STATISTICAL ANALYSES PERFORMED: Descriptive and bivariate analyses were performed and significant associations (P<0.05) used to build the full multiple linear regression model. Meal and shopping variables were forced into the model as predictors of follow-up SCREEN questionnaire scores. RESULTS: Meals On Wheels use was independently associated with higher SCREEN questionnaire scores (ie, less risk), as was higher income. Baseline SCREEN questionnaire scores also strongly and positively predicted follow-up scores. Self-reported depression at baseline was associated with lower scores at follow-up. Although use of programs at baseline was associated with decreased risk, if participants experienced increased use of the program (eg, more meals) during the follow-up period this was associated with lower scores, or increased risk. CONCLUSIONS: Meal programs can improve or maintain nutritional risk for vulnerable seniors. Increased use of these programs over time may indicate a senior's declining status. Seniors who are in need of informal or formal supports for food shopping or preparation should be encouraged to participate in meal programs as a means of maintaining or improving their nutrition
Notes: DA - 20060703
IS - 0002-8223 (Print)
IS - 0002-8223 (Linking)
LA - eng
PT - Journal Article
SB - AIM
SB - IM
	59. 	Kimura M, Moriyasu A, Kumagai S et al. Community-based intervention to improve dietary habits and promote physical activity among older adults: a cluster randomized trial. BMC.Geriatr. 2013;13:8.
Ref ID: 83
Keywords: Aged/Aged,80 and over/Cluster Analysis/Female/Food Habits/physiology/psychology/Humans/Male/Motor Activity/Residence Characteristics/Risk Reduction Behavior
Reprint: Not in File
Abstract: BACKGROUND: The fastest growing age group globally is older adults, and preventing the need for long-term nursing care in this group is important for social and financial reasons. A population approach to diet and physical activity through the use of social services can play an important role in prevention. This study examined the effectiveness of a social health program for community-dwelling older adults aimed at introducing and promoting physical activity in the home at each individual's pace, helping participants maintain good dietary habits by keeping self-check sheets, and determining whether long-standing unhealthy or less-than-ideal physical and dietary habits can be changed. METHOD: This cluster randomized trial conducted at 6 community centers in an urban community involved 92 community-dwelling older adults aged 65-90 years. The intervention group (3 community centers; n = 57) participated in the social health program "Sumida TAKE10!" which is an educational program incorporating the "TAKE10!(R) for Older Adults" program, once every 2 weeks for 3 months. The control group (3 community centers; n=35) was subsequently provided with the same program as a crossover intervention group. The main outcome measures were changes in food intake frequency, food frequency score (FFS), dietary variety score (DVS), and frequency of walking and exercise. The secondary outcome measures were changes in self-rated health, appetite, and the Tokyo Metropolitan Institute of Gerontology (TMIG) Index of Competence score. RESULTS: Compared to baseline, post-intervention food intake frequency for 6 of 10 food groups (meat, fish/shellfish, eggs, potatoes, fruits, and seaweed), FFS, and DVS were significantly increased in the intervention group, and interaction effects of FFS and DVS were seen between the two groups. No significant differences were observed between baseline and post-intervention in the control group. Frequency of walking and exercise remained unchanged in both groups, and no significant difference in improvement rate was seen between the groups. Self-rated health was significantly increased in the intervention group. Appetite and TMIG Index of Competence score were unchanged in both groups. CONCLUSIONS: The social health program resulted in improved dietary habits, as measured by food intake frequency, FFS, and DVS, and may improve self-rated health among community-dwelling older adults. TRIAL REGISTRATION NUMBER: UMIN000007357
Notes: DA - 20130131
IS - 1471-2318 (Electronic)
IS - 1471-2318 (Linking)
LA - eng
PT - Journal Article
PT - Multicenter Study
PT - Randomized Controlled Trial
PT - Research Support, Non-U.S. Gov't
SB - IM
	60. 	Knight E, Petrella RJ. Prescribing physical activity for healthy aging: longitudinal follow-up and mixed method analysis of a primary care intervention. Phys.Sportsmed. 2014;42:30-8.
Ref ID: 44
Keywords: Aged/Counseling/methods/organization & administration/Exercise/physiology/Female/Health Behavior/Health Promotion/Humans/Life Style/Male/Middle Aged/Motor Activity/Oxygen Consumption/Primary Health Care/Telemedicine
Reprint: Not in File
Abstract: BACKGROUND: There is a shortage of literature describing the experience of individuals who have participated in a physical activity and mobile health (mHealth) intervention. Many physical activity interventions are of short duration and do not report long-term changes in clinical measures or adoption of prescribed health behaviors. Previously, we have reported the clinical and behavioral outcomes from the first phase of a physical activity prescription and mHealth intervention delivered through the primary care setting. The purpose of this next phase is to perform a longitudinal follow-up 6-months postintervention. METHODS: Mixed methods analysis including repeated measures ANOVA of functional aerobic capacity (VO2max) at preintervention, postintervention, and follow-up clinic visits, and whole text analysis of semistructured interviews discussing the participant experience in a health behavior intervention. RESULTS: Twenty participants, mean age 63 +/- 5 years, participated. Gains made in VO2max were maintained at 6 months (P < 0.05). Participants reported engaging in sustained and routine physical activity, yet some identified a need for additional support to adopt the prescribed health behaviors. Emergent themes included the desire for short-term mHealth intervention to educate individuals about prescribed health behaviors without need for ongoing management by clinicians, leveraging mHealth to build social networks around prescribed health behaviors and to connect individuals to build a sense of community, and participant views of physical activity as medicine. CONCLUSIONS: The present study investigated both the long-term adoption of physical activity behaviors as well as the participant experience in a physical activity and mHealth intervention. Findings from the current study may be used to inform the development of user-centered lifestyle interventions
Notes: DA - 20141125
IS - 0091-3847 (Print)
IS - 0091-3847 (Linking)
LA - eng
PT - Journal Article
PT - Randomized Controlled Trial
SB - IM
	61. 	Kogan AC, Gonzalez J, Hart B et al. Be Well: results of a nutrition, exercise, and weight management intervention among at-risk older adults. J.Appl.Gerontol. 2013;32:889-901.
Ref ID: 64
Keywords: Aged/Aged,80 and over/Chronic Disease/epidemiology/Cohort Studies/Counseling/Depression/diagnosis/therapy/Exercise/Exercise Test/Female/Health Education/Humans/Los Angeles/Male/Middle Aged/Nutrition Assessment/Physical Fitness/Senior Centers/Weight Reduction Programs
Reprint: Not in File
Abstract: The objective of this article is to test the effectiveness of a multifaceted exercise and nutritional education intervention for chronically ill, community-dwelling older adults. A pre/post cohort design was implemented with measures of physical activity, fitness, depression, and anthropometry collected via 4-month in-person interview and telephone follow-up. The study was conducted at two community-based senior centers in the Los Angeles area and participants (n=62) were older adults aged 60 or older, with multiple chronic conditions, with one or more emergency department visits or hospital admissions in the previous 6 months, and at nutritionally moderate to high risk. The intervention was a fitness program providing nutritional counseling, low-impact exercise, and weight management. Results revealed significant improvements for hours of weekly exercise (Z = -4.3, p < .001), daily walking distance (Z = -5.7, p < .001), performance on fitness tests, depression (Z = 3.9, p < .001), and body measurements were observed. Findings speak to the healthy benefits of exercise and good nutrition as possible alternatives or adjuncts to pharmacotherapy for weight loss and depression
Notes: DA - 20141205
IS - 1552-4523 (Electronic)
IS - 0733-4648 (Linking)
LA - eng
PT - Journal Article
PT - Research Support, Non-U.S. Gov't
SB - IM
	62. 	Laforest S, Pelletier A, Gauvin L et al. Impact of a community-based falls prevention program on maintenance of physical activity among older adults. J.Aging Health 2009;21:480-500.
Ref ID: 150
Keywords: Accidental Falls/prevention & control/Aged/Canada/Community Health Services/methods/Energy Metabolism/Exercise/Exercise Therapy/Female/Health Promotion/Humans/Male/Middle Aged/Motor Activity/Preventive Health Services/Treatment Outcome
Reprint: Not in File
Abstract: OBJECTIVE: This study examines the 9-month impact of a 12-week falls prevention program (called Stand Up!) which included balance exercises and educational components on maintenance of physical activity among community-dwelling seniors. METHOD: Data were collected among 98 experimental and 102 control participants at baseline, immediately after the program and 9 months later. Involvement in physical activity was measured with three indicators. Program effects were examined using linear and logistic regression procedures. RESULTS: Both groups showed similar increases in weekly frequency of exercise at the 9-month posttest. However, the program's participants showed higher increases in their variety of exercises at the 9-month posttest (especially among those with greater baseline scores). Among seniors reporting lower levels of energy expenditure at baseline, the program's participants showed significantly greater increases in energy expenditure than control participants. DISCUSSION: These preliminary findings suggest that programs such as Stand Up! have the potential to stimulate continued involvement in physical activity
Notes: DA - 20090325
IS - 0898-2643 (Print)
IS - 0898-2643 (Linking)
LA - eng
PT - Journal Article
PT - Research Support, Non-U.S. Gov't
SB - T
	63. 	Laforest S, Goldin B, Nour K, Roy MA, Payette H. Nutrition risk in home-bound older adults: using dietician-trained and supervised nutrition volunteers for screening and intervention. Can.J.Aging 2007;26:305-15.
Ref ID: 165
Keywords: Aged/Aged,80 and over/Aging/Feasibility Studies/Female/Geriatric Assessment/Health Services for the Aged/Health Status Indicators/Home Care Services/Homebound Persons/statistics & numerical data/Humans/Male/Nutrition Assessment/Nutrition Surveys/Pilot Projects/Quebec/Risk Assessment/Surveys and Questionnaires/Volunteers
Reprint: Not in File
Abstract: Nutrition screening and early intervention in home-bound older adults are key to preventing unfavourable health outcomes and functional decline. This pilot study's objectives were (a) to test the reliability of the Elderly Nutrition Screening Tool (ENS) when administered by dietician-trained and supervised nutrition volunteers, and (b) to explore the feasibility of volunteers' doing nutrition screening and intervention for home-bound older adults receiving home care services. Both participating clients ( n = 29) and volunteers ( n = 15) were community-dwelling older adults. Volunteers met with participating clients, assessed nutritional risk with the ENS, provided nutritional education, and developed and helped implement intervention plans. To assess ENS (c) inter-rater reliability, we compared results obtained by nutrition volunteers and a dietician. Agreement was high (> or =80%) for most items but was higher among volunteers than between volunteers and the dietician. We conclude that nutrition volunteers can assist in screening and educating older adults regarding nutritional risks, but intervention is best left to professionals
Notes: DA - 20080228
IS - 0714-9808 (Print)
IS - 0714-9808 (Linking)
LA - eng
PT - Comparative Study
PT - Journal Article
PT - Research Support, Non-U.S. Gov't
SB - IM
	64. 	Lammes E, Rydwik E, Akner G. Effects of nutritional intervention and physical training on energy intake, resting metabolic rate and body composition in frail elderly. a randomised, controlled pilot study. J.Nutr Health Aging 2012;16:162-7.
Ref ID: 105
Keywords: Aged/Aged,80 and over/Basal Metabolism/physiology/Body Composition/Calorimetry,Indirect/Energy Intake/Exercise/Female/Follow-Up Studies/Frail Elderly/Humans/Male/Nutritional Sciences/education/Pilot Projects/Treatment Outcome
Reprint: Not in File
Abstract: OBJECTIVES: To analyse the effect of nutritional intervention and physical training on energy intake, resting metabolic rate (RMR) and body composition in the frail elderly. DESIGN: Open, randomised, controlled pilot treatment study. SETTING: Community-based research centre. PARTICIPANTS: Ninety-six community-dwelling frail elderly people aged 75 and older, 40% men. INTERVENTION: Four treatment arms: i) individual nutritional advice and group sessions on nutrition for the elderly, ii) physical training 2 x 45 minutes per week for 3 months, iii) combined nutritional and physical intervention and iv) control group. MEASUREMENTS: The outcomes were energy intake (4-day food diary); resting metabolic rate (indirect calorimetry) and body composition (anthropometry) performed at baseline, after 3 months' intervention (completed by 79 individuals), and as a follow-up at 9 months (completed by 64 individuals). RESULTS: The training group showed a significantly increased RMR at 3 months. Otherwise, there were no observed differences within or between the four groups. There was no correlation over time between energy intake, RMR and fat free mass. The participants with a low energy intake who managed to increase their energy intake during the study ('responders') had a statistically significantly lower BMI (21 vs. 24) and a lower fat percentage (23 vs. 30) at baseline than the 'non-responders'. The 'non-responders' showed a small but statistically significant decrease in body fat percentage at F1, and in body weight, BMI and FFM at 9 months (F3). CONCLUSION: Individual nutrition counselling and physical exercise had no effect on energy intake, RMR or fat free mass in community-dwelling frail elderly people aged 75 and older. Interventions in frail elderly people should be targeted according to the needs of the individual patients. The issues of randomisation, targeting and responders in are problematised and discussed
Notes: DA - 20120210
IS - 1760-4788 (Electronic)
IS - 1279-7707 (Linking)
LA - eng
PT - Journal Article
PT - Randomized Controlled Trial
PT - Research Support, Non-U.S. Gov't
SB - IM
	65. 	Lanuez MV, Lanuez FV, Montero EG, Jacob FW. Correlation between two physical activity programs in the gait of sedentary elderly subjects. Einstein.(Sao Paulo) 2010;8:281-4.
Ref ID: 131
Reprint: Not in File
Abstract: OBJECTIVES: To assess the effect of exercise on gait using two different programs: a group of aerobic exercises (Group A, n = 18) and a group of flexibility and balance exercises (Group B, n = 19). METHODS: A casualized controlled study, in which each sample controlled itself, was undertaken. The sample comprised 37 male and female subjects, aged from 60 to 90 years, from the outpatient clinic of the Geriatrics Unit of Hospital das Clinicas of Faculdade de Medicina of Universidade de Sao Paulo; the patients were sedentary and had not exercised regularly during the past six months. RESULTS: Improvement of gait was seen mainly in the group that did specific exercises. CONCLUSION: The results of this study underline the importance of physical exercises in sedentary elderly subjects, but show the need for programming the exercises towards specific goals, which can optimize the results of this tool of health promotion for the elderly
Notes: DA - 20160114
IS - 1679-4508 (Print)
IS - 1679-4508 (Linking)
LA - eng
LA - por
PT - Journal Article
	66. 	Lara J, Turbett E, Mckevic A, Rudgard K, Hearth H, Mathers JC. The Mediterranean diet among British older adults: Its understanding, acceptability and the feasibility of a randomised brief intervention with two levels of dietary advice. Maturitas 2015;82:387-93.
Ref ID: 24
Reprint: Not in File
Abstract: OBJECTIVES: To assess (i) understanding, acceptability and preference for two graphical displays of the Mediterranean diet (MD); and (ii) feasibility of a brief MD intervention and cost of adherence to this diet among British older adults. DESIGN: Two studies undertaken at the Human Nutrition Research Centre, Newcastle University are reported. In study-1, preference and understanding of the MD guidelines and two graphical displays, a plate and a pyramid, were evaluated in an educational group session (EGS). In study-2, we evaluated the feasibility of a three-week brief MD intervention with two levels of dietary advice: Group-1 (level 1) attended an EGS on the MD, and Group-2 (level 2) attended an EGS and received additional support. MD adherence using a 9-point score, and the cost of food intake during intervention, were assessed. RESULTS STUDY-1: No differences in preference for a MD plate or pyramid were observed. Both graphic displays were rated as acceptable and conveyed clearly these guidelines. STUDY-2: The intervention was rated as acceptable. No significant differences were observed between groups 1 and 2. Analysis of the combined sample showed significant increases from baseline in fish intake (P=0.01) and MD score (P=0.05). The cost of food intake during intervention was not significantly different from baseline. CONCLUSION: British older adults rated a MD as an acceptable model of healthy eating, and a plate and a pyramid as comprehensible graphic displays of these guidelines. A brief dietary intervention was also acceptable and revealed that greater adherence to the MD could be achieved without incurring significantly greater costs
Notes: DA - 20151116
IS - 1873-4111 (Electronic)
IS - 0378-5122 (Linking)
LA - eng
PT - Journal Article
PT - Research Support, Non-U.S. Gov't
SB - IM
	67. 	Lee AH, Jancey J, Howat P, Burke L, Kerr DA, Shilton T. Effectiveness of a home-based postal and telephone physical activity and nutrition pilot program for seniors. J.Obes. 2011;2011.
Ref ID: 130
Reprint: Not in File
Abstract: Objective. To evaluate the effectiveness of a 12-week home-based postal and telephone physical activity and nutrition pilot program for seniors. Methods. The program was delivered by mailed material and telephone calls. The main intervention consisted of a booklet tailored for seniors containing information on dietary guidelines, recommended physical activity levels, and goal setting. Dietary and walking activity outcomes were collected via a self-administered postal questionnaire pre- and postintervention and analysed using linear mixed regressions. Of the 270 seniors recruited, half were randomly selected for the program while others served as the control group. Results. The program elicited favourable responses. Postintervention walking for exercise/recreation showed an average gain of 27 minutes per week for the participants in contrast to an average drop of 5 minutes for the controls (P < .01). Little change was evident in errand walking for both groups. The intervention group (n = 114) demonstrated a significant increase in fibre intake (P < .01) but no reduction in fat intake (P > .05) compared to controls (n = 134). Conclusions. The participants became more aware of their health and wellbeing after the pilot program, which was successful in increasing time spent walking for recreation and improving fibre intake
Notes: DA - 20100917
IS - 2090-0716 (Electronic)
IS - 2090-0708 (Linking)
LA - eng
PT - Journal Article
	68. 	Lee JS, Frongillo EA, Olson CM. Understanding targeting from the perspective of program providers in the elderly nutrition program. J.Nutr Elder. 2005;24:25-45.
Ref ID: 190
Keywords: Adult/Aged/Female/Food Services/economics/organization & administration/Humans/Interviews as Topic/Male/Middle Aged/Needs Assessment/Program Evaluation/Reproducibility of Results
Reprint: Not in File
Abstract: Providers' perspectives on need, strategies, and challenges are critical in targeting of programs. This study was undertaken to understand the meanings and challenges of targeting from the perspectives of program providers in the Elderly Nutrition Program (ENP). Qualitative in-depth interviews were done with a sample of 36 local ENP providers from six counties in upstate New York. Each interview was tape-recorded, transcribed, and analyzed. Qualitative analysis revealed four distinct meanings of targeting including maximum outreach, reaching out to the most needy, serving only the most needy, and evaluating coverage. Regardless of the meaning or type of targeting, local providers reported universal challenges for outreach, need assessment, and resources in implementing targeting. Understanding these different meanings about targeting could contribute to crafting better targeting policies and program procedures to deliver adequate nutrition service to elders in need in the community
Notes: DA - 20050524
IS - 0163-9366 (Print)
IS - 0163-9366 (Linking)
LA - eng
PT - Journal Article
PT - Research Support, U.S. Gov't, Non-P.H.S
SB - IM
	69. 	Legault C, Jennings JM, Katula JA et al. Designing clinical trials for assessing the effects of cognitive training and physical activity interventions on cognitive outcomes: the Seniors Health and Activity Research Program Pilot (SHARP-P) study, a randomized controlled trial. BMC.Geriatr. 2011;11:27.
Ref ID: 120
Keywords: Aged/Aged,80 and over/Cognition Disorders/prevention & control/psychology/therapy/Cognitive Therapy/methods/Cohort Studies/Humans/Learning/physiology/Motor Activity/Pilot Projects/Single-Blind Method/Treatment Outcome
Reprint: Not in File
Abstract: BACKGROUND: The efficacy of non-pharmacological intervention approaches such as physical activity, strength, and cognitive training for improving brain health has not been established. Before definitive trials are mounted, important design questions on participation/adherence, training and interventions effects must be answered to more fully inform a full-scale trial. METHODS: SHARP-P was a single-blinded randomized controlled pilot trial of a 4-month physical activity training intervention (PA) and/or cognitive training intervention (CT) in a 2 x 2 factorial design with a health education control condition in 73 community-dwelling persons, aged 70-85 years, who were at risk for cognitive decline but did not have mild cognitive impairment. RESULTS: Intervention attendance rates were higher in the CT and PACT groups: CT: 96%, PA: 76%, PACT: 90% (p=0.004), the interventions produced marked changes in cognitive and physical performance measures (p</=0.05), and retention rates exceeded 90%. There were no statistically significant differences in 4-month changes in composite scores of cognitive, executive, and episodic memory function among arms. Four-month improvements in the composite measure increased with age among participants assigned to physical activity training but decreased with age for other participants (intervention*age interaction p=0.01). Depending on the choice of outcome, two-armed full-scale trials may require fewer than 1,000 participants (continuous outcome) or 2,000 participants (categorical outcome). CONCLUSIONS: Good levels of participation, adherence, and retention appear to be achievable for participants through age 85 years. Care should be taken to ensure that an attention control condition does not attenuate intervention effects. Depending on the choice of outcome measures, the necessary sample sizes to conduct four-year trials appear to be feasible. TRIAL REGISTRATION: Clinicaltrials.gov Identifier: NCT00688155
Notes: DA - 20110630
IS - 1471-2318 (Electronic)
IS - 1471-2318 (Linking)
LA - eng
PT - Comparative Study
PT - Journal Article
PT - Randomized Controlled Trial
PT - Research Support, N.I.H., Extramural
PT - Research Support, Non-U.S. Gov't
SB - IM
	70. 	Lin YH, Chu LL, Kao CC, Chen TB, Lee I, Li HC. The Effects of a Diet and Exercise Program for Older Adults With Metabolic Syndrome. J.Nurs.Res. 2015;23:197-205.
Ref ID: 41
Reprint: Not in File
Abstract: BACKGROUND: The prevalence of metabolic syndrome is high among older adults in Taiwan. However, few studies have studied the effect of a combined diet and exercise program on managing metabolic syndrome (MetS) in individuals 65 years and older and living in Taiwan's rural areas. PURPOSE: This study tests the effectiveness of a diet and exercise program on the MetS biomarkers in older community residents with MetS. METHODS: This study used a quasiexperimental study design. All participants were 65 years and older and were diagnosed with MetS. The outcome variables included biomarkers (blood pressure, waist circumference, hip circumference, body mass index, blood sugar, cholesterol, and triglycerides) and demographic characteristics. The participants were distributed into a diet-and-exercise group (n = 163) and a nondiet-and-nonexercise group (n = 138). The outcome variables were examined 3 months after the start of the intervention program. RESULTS: The participants in the diet-and-exercise group had lower values than the nondiet-and-nonexercise group for blood pressure, waist circumference, hip circumference, body mass index, blood sugar, cholesterol, and triglyceride (all ps < .001). CONCLUSIONS/IMPLICATIONS FOR PRACTICE: The diet and exercise program is an effective intervention for treating older individuals with MetS. Clear and concise information concerning the effects of diet and exercise in promoting the health of older residents with MetS is helpful to improve the health of the older adults inTaiwan
Notes: DA - 20150814
IS - 1948-965X (Electronic)
IS - 1682-3141 (Linking)
LA - eng
PT - Journal Article
SB - IM
SB - N
	71. 	Liu CK, Leng X, Hsu FC et al. The impact of sarcopenia on a physical activity intervention: the Lifestyle Interventions and Independence for Elders Pilot Study (LIFE-P). J.Nutr Health Aging 2014;18:59-64.
Ref ID: 56
Keywords: Absorptiometry,Photon/Aged/Aged,80 and over/Aging/physiology/Body Composition/Exercise/Female/Gait/Geriatric Assessment/Humans/Independent Living/Life Style/Male/Mobility Limitation/Physical Fitness/Pilot Projects/Sarcopenia/complications/physiopathology/therapy
Reprint: Not in File
Abstract: OBJECTIVE: To determine if sarcopenia modulates the response to a physical activity intervention in functionally limited older adults. DESIGN: Secondary analysis of a randomized controlled trial. SETTING: Three academic centers. PARTICIPANTS: Elders aged 70 to 89 years at risk for mobility disability who underwent dual-energy x-ray absorptiometry (DXA) for body composition at enrollment and follow-up at twelve months (N = 177). INTERVENTION: Subjects participated in a physical activity program (PA) featuring aerobic, strength, balance, and flexibility training, or a successful aging (SA) educational program about healthy aging. MEASUREMENTS: Sarcopenia as determined by measuring appendicular lean mass and adjusting for height and total body fat mass (residuals method), Short Physical Performance Battery score (SPPB), and gait speed determined on 400 meter course. RESULTS: At twelve months, sarcopenic and non-sarcopenic subjects in PA tended to have higher mean SPPB scores (8.7+/-0.5 and 8.7+/-0.2 points) compared to sarcopenic and non-sarcopenic subjects in SA (8.3+/-0.5 and 8.4+/-0.2 points, p = 0.24 and 0.10), although the differences were not statistically significant. At twelve months, faster mean gait speeds were observed in PA: 0.93+/-0.4 and 0.95+/-0.03 meters/second in sarcopenic and non-sarcopenic PA subjects, and 0.89+/-0.4 and 0.91+/-0.03 meters/second in sarcopenic and non-sarcopenic SA subjects (p = 0.98 and 0.26), although not statistically significant. There was no difference between the sarcopenic and non-sarcopenic groups in intervention adherence or number of adverse events. CONCLUSION: These data suggest that older adults with sarcopenia, who represent a vulnerable segment of the elder population, are capable of improvements in physical performance after a physical activity intervention
Notes: DA - 20140109
IS - 1760-4788 (Electronic)
IS - 1279-7707 (Linking)
LA - eng
PT - Journal Article
PT - Randomized Controlled Trial
PT - Research Support, N.I.H., Extramural
PT - Research Support, N.I.H., Intramural
PT - Research Support, Non-U.S. Gov't
PT - Research Support, U.S. Gov't, Non-P.H.S
PT - Research Support, U.S. Gov't, P.H.S
SB - IM
	72. 	Lloyd JL, Wellman NS. Older Americans Act Nutrition Programs: A Community-Based Nutrition Program Helping Older Adults Remain at Home. J.Nutr Gerontol.Geriatr. 2015;34:90-109.
Ref ID: 32
Keywords: Aged/Aged,80 and over/Community Health Services/economics/legislation & jurisprudence/statistics & numerical data/Consumer Behavior/Financing,Government/Food Quality/Food Services/Food Supply/Health Services for the Aged/Health Status/Homebound Persons/Humans/Malnutrition/prevention & control/Nutritional Physiological Phenomena/Nutritional Requirements/Poverty/United States
Reprint: Not in File
Abstract: Nutrition interventions are important as the older population, most of whom live in the community, increases in size and diversity. They are key to leading a healthy, functional life and mitigating chronic health conditions. The Older Americans Act Nutrition Program served 86.3 million congregate and 137.4 million home-delivered meals to 1.6 million and 850,000 older adults, respectively (2012). Congregate and home-delivered participants were older, poorer, sicker, more functionally impaired, and at a greater risk of institutionalization than the general U.S. older population. The Nutrition Program is publically and privately funded. About 44% of congregate and 30% of home-delivered expenditures are from federal sources, which dropped from $25 per older adult in 1990 to $12 in 2013. Despite multiple funding sources, funding is insufficient for the expanding older population. Health, nutrition, and social service professionals need to coordinate their community-based services to truly help older adults remain in their homes
Notes: DA - 20150625
IS - 2155-1200 (Electronic)
LA - eng
PT - Journal Article
SB - IM
SB - N
	73. 	Locher JL, Vickers KS, Buys DR et al. A randomized controlled trial of a theoretically-based behavioral nutrition intervention for community elders: lessons learned from the Behavioral Nutrition Intervention for Community Elders Study. J.Acad.Nutr Diet. 2013;113:1675-82.
Ref ID: 66
Keywords: Aged/Aged,80 and over/Behavior Therapy/methods/Body Mass Index/Energy Intake/Female/Home Care Services/Homebound Persons/Humans/Male/Malnutrition/therapy/Prospective Studies/Self Care/Weight Gain
Reprint: Not in File
Abstract: Older adults with multiple comorbidities are often undernourished or at high risk for becoming so, especially after a recent hospitalization. Randomized controlled trials of effective, innovative interventions are needed to support evidence-based approaches for solving nutritional problems in this population. Self-management approaches where participants select their own behavioral goals can enhance success of interventions. The purpose of this study was to evaluate the feasibility and efficacy of a multilevel self-management intervention to improve nutritional status in a group of high-risk older adults. The Behavioral Nutrition Intervention for Community Elders (B-NICE) trial used a prospective randomized controlled design to determine whether the intervention, compared to standard care, maintained or increased caloric intake (depending on baseline body mass index) and, consequently, stabilized or increased body weight. Participants were 34 Medicare-eligible, age 65 years old or older, homebound adults who were consuming insufficient calories and/or had a history of weight loss >/=2.5% over 6 months. The intervention took place within participants' homes. Outcome measures, including energy intake (based on collection of three 24-hour dietary recalls) and body weights were assessed at baseline and at 60 days post randomization. The primary analyses included analyses of covariance and Pearson's chi(2). We hypothesized that the intervention would result in increased caloric intake and weight gain in underweight older adults and increased or stabilized caloric intake and weight for everyone else. The intervention was feasible; however, it did not result in differences between groups for desired outcomes of either caloric intake or body weight. Future interventions might either deliberately involve caregivers or reduce burden for both patients and caregivers
Notes: DA - 20131118
IS - 2212-2672 (Print)
LA - eng
PT - Journal Article
PT - Randomized Controlled Trial
PT - Research Support, N.I.H., Extramural
SB - AIM
SB - IM
	74. 	Locher JL, Bales CW, Ellis AC et al. A theoretically based Behavioral Nutrition Intervention for Community Elders at high risk: the B-NICE randomized controlled clinical trial. J.Nutr Gerontol.Geriatr. 2011;30:384-402.
Ref ID: 108
Keywords: Aged/Attitude to Health/Behavior Therapy/Counseling/Diet/Energy Intake/Evaluation Studies as Topic/Female/Health Behavior/Health Policy/Home Care Services/Humans/Male/Malnutrition/prevention & control/Medicare/Prospective Studies/Psychological Theory/Risk/Self Care/Treatment Outcome/United States
Reprint: Not in File
Abstract: We conducted a study designed to evaluate the efficacy and feasibility of a multilevel self-management intervention to improve nutritional intake in a group of older adults receiving Medicare home health services who were at especially high risk for experiencing undernutrition. The Behavioral Nutrition Intervention for Community Elders (B-NICE) trial used a prospective randomized controlled design to determine whether individually tailored counseling focused on social and behavioral aspects of eating resulted in increased caloric intake and improved nutrition-related health outcomes in a high-risk population of older adults. The study was guided by the theoretical approaches of the Ecological Model and Social Cognitive Theory. The development and implementation of the B-NICE protocol, including the theoretical framework, methodology, specific elements of the behavioral intervention, and assurances of the treatment fidelity, as well as the health policy implications of the trial results, are presented in this article
Notes: DA - 20111121
IS - 2155-1200 (Electronic)
LA - eng
PT - Journal Article
PT - Randomized Controlled Trial
PT - Research Support, N.I.H., Extramural
SB - IM
SB - N
	75. 	Lubans DR, Sylva K. Mediators of change following a senior school physical activity intervention. J.Sci.Med.Sport 2009;12:134-40.
Ref ID: 168
Keywords: Adolescent/Adolescent Behavior/psychology/Exercise/Female/Health Behavior/Health Promotion/methods/Humans/Male/Motor Activity/Peer Group/Schools/Self Efficacy/Social Support/Students
Reprint: Not in File
Abstract: It has been suggested that the low level of effectiveness of youth interventions is due to a lack of knowledge regarding the mechanisms responsible for behaviour change. The identification of behaviour mediators is necessary for the progression of physical activity research, as it allows researchers to determine which components of an intervention are responsible for mediating behaviour change. The purpose of this study was to identify mediators of behaviour change in a physical activity intervention for senior school students. Participants (n=78) were randomly allocated to control or intervention conditions for a period of 10 weeks. Moderate-to-vigorous physical activity (MVPA) and potential mediators were assessed at baseline and post-intervention (10 weeks). Hypothesized mediators were derived from Bandura's Social Cognitive Theory and included: peer support, exercise self-efficacy and outcome expectancy. Mediation was assessed using the product-of-coefficients test described by MacKinnon and colleagues, based on the criteria for mediation identified by Baron and Kenny. While none of the variables satisfied all four criteria for mediation among males or females, self-efficacy was able to satisfy the first three criteria among females in the study. Exercise self-efficacy may be a mediator of physical activity behaviour in adolescent girls
Notes: DA - 20090121
IS - 1440-2440 (Print)
LA - eng
PT - Journal Article
PT - Randomized Controlled Trial
SB - IM
	76. 	Luten KA, Dijkstra A, Reijneveld SA, de Winter AF. Moderators of physical activity and healthy eating in an integrated community-based intervention for older adults. Eur.J.Public Health 2016.
Ref ID: 2
Reprint: Not in File
Abstract: BACKGROUND: An integrated community-based intervention was developed to stimulate physical activity (PA) and healthy eating in older adults in a socioeconomically disadvantaged area. This study aims to assess whether its short-term effects among older adults vary by sociodemographic, psychosocial and health-related variables. METHODS: The study was a controlled pre-post quasi-experimental design (intervention conditionn= 430; control conditionn= 213), with a baseline measurement and a 9-month follow-up measurement. The intervention consisted of a local media campaign and environmental approaches. Changes in PA and fruit and vegetable consumption (FVC) were dependent variables assessed at 9-month follow-up. Sociodemographic, psychosocial and health-related variables at baseline were tested as potential moderators of the effects of the conditions. RESULTS: We found different types of moderators in particular for transport-related PA and FVC. Regarding sociodemographic characteristics, gender was a moderator for household-related PA, and educational level for transport-related PA and FVC. Self-efficacy, as a psychosocial variable, was a moderator of transport-related PA and vegetable consumption. Concerning health-related outcomes, baseline levels of transport-related PA and fruit consumption were moderators for transport-related PA and fruit consumption. If adjusted for multiple testing, only three moderators persisted: educational level regarding vegetable consumption, and baseline levels regarding transport-related PA and fruit consumption. CONCLUSION: The effects of the community intervention vary somewhat by sociodemographic, psychosocial and health-related variables. The intervention seems to be especially beneficial to those who are most in need of more PA and healthy eating
Notes: DA - 20160420
IS - 1464-360X (Electronic)
IS - 1101-1262 (Linking)
LA - ENG
PT - JOURNAL ARTICLE
	77. 	Luten KA, Reijneveld SA, Dijkstra A, de Winter AF. Reach and effectiveness of an integrated community-based intervention on physical activity and healthy eating of older adults in a socioeconomically disadvantaged community. Health Educ.Res. 2016;31:98-106.
Ref ID: 17
Reprint: Not in File
Abstract: The aim of this study is to assess the reach and effectiveness of an integrated community-based intervention designed to promote physical activity and healthy eating among older adults in a socioeconomically disadvantaged community in the Netherlands. The intervention was evaluated with a controlled pre-post quasi-experimental design, with 430 randomly selected older adults participating in the intervention group and 213 in a control group at baseline. The intervention included a local media campaign and environmental approaches (e.g. community involvement) and was implemented during a 3-month high-intensity period, followed by a 6-month low-intensity one. Levels of physical activity and fruit and vegetable consumption were assessed at baseline and at 3 and 9 months after baseline. At the follow-up measurements, the intervention had reached respectively 68 and 69% of the participants in the intervention group. No significant differences were found between the intervention group and the control group in changes to any outcome except for transport-related PA at 3 and 9 months follow-up. The systematically developed community-based intervention reached a relatively large proportion of the participants, but had only small effects on the levels of physical activity and healthy eating in older adults in the short and medium term
Notes: DA - 20160120
IS - 1465-3648 (Electronic)
IS - 0268-1153 (Linking)
LA - eng
PT - Journal Article
SB - T
	78. 	Manilla B, Keller HH, Hedley MR. Food tasting as nutrition education for older adults. Can.J.Diet.Pract.Res. 2010;71:99-102.
Ref ID: 132
Keywords: Aged/Aged,80 and over/Exploratory Behavior/Female/Food Preferences/psychology/Health Promotion/methods/Humans/Male/Middle Aged/Nutritional Sciences/education/Ontario/Pilot Projects
Reprint: Not in File
Abstract: PURPOSE: The likelihood of experiencing poor nutrition status increases as people age. Sampling new foods may promote a continued interest in food and enjoyment of eating. This pilot study was designed to describe and provide a preliminary evaluation of food-tasting activities integrated into nutrition displays directed at community-living older adults. METHODS: Three consecutive monthly nutrition displays incorporating a food-tasting activity were presented at a Guelph, Ontario, recreation centre for seniors. Seniors had an opportunity to taste two recipes at each of three displays; 226 food samples and 155 copies of recipes were taken. Feedback forms were used to determine participants' interest in making the recipes, and whether tasting influenced their interest in preparing the food. RESULTS: Among 54 participants who completed feedback forms about the program, 75.9% indicated that they intended to prepare one or both of the recipes tasted at the display; 70.4% indicated that they would not or may not have made the recipe without tasting it beforehand. CONCLUSIONS: Dietitians working with community-living older adults could use food tastings to help translate key educational messages into practice, and to encourage eating enjoyment as people age
Notes: DA - 20100607
IS - 1486-3847 (Print)
IS - 1486-3847 (Linking)
LA - eng
PT - Journal Article
PT - Research Support, Non-U.S. Gov't
SB - IM
	79. 	Marques AI, Santos L, Soares P et al. A proposed adaptation of the European Foundation for Quality Management Excellence Model to physical activity programmes for the elderly - development of a quality self-assessment tool using a modified Delphi process. Int.J.Behav.Nutr Phys.Act. 2011;8:104.
Ref ID: 113
Keywords: Aged/Aging/Consensus/Delivery of Health Care/Delphi Technique/Europe/Exercise/Geriatrics/Health Promotion/Humans/Internet/Practice Guidelines as Topic/Program Evaluation/methods/Quality Control/Sports
Reprint: Not in File
Abstract: BACKGROUND: There has been a growing concern in designing physical activity (PA) programmes for elderly people, since evidence suggests that such health promotion interventions may reduce the deleterious effects of the ageing process. Complete programme evaluations are a necessary prerequisite to continuous quality improvements. Being able to refine, adapt and create tools that are suited to the realities and contexts of PA programmes for the elderly in order to support its continuous improvement is, therefore, crucial. Thus, the aim of this study was to develop a self-assessment tool for PA programmes for the elderly. METHODS: A 3-round Delphi process was conducted via the Internet with 43 national experts in PA for the elderly, management and delivery of PA programmes for the elderly, sports management, quality management and gerontology, asking experts to identify the propositions that they considered relevant for inclusion in the self-assessment tool. Experts reviewed a list of proposed statements, based on the criteria and sub-criteria from the European Foundation for Quality Management Excellence Model (EFQM) and PA guidelines for older adults and rated each proposition from 1 to 8 (disagree to agree) and modified and/or added propositions. Propositions receiving either bottom or top scores of greater than 70% were considered to have achieved consensus to drop or retain, respectively. RESULTS: In round 1, of the 196 originally-proposed statements (best practice principles), the experts modified 41, added 1 and achieved consensus on 93. In round 2, a total of 104 propositions were presented, of which experts modified 39 and achieved consensus on 53. In the last round, of 51 proposed statements, the experts achieved consensus on 19. After 3 rounds of rating, experts had not achieved consensus on 32 propositions. The resulting tool consisted of 165 statements that assess nine management areas involved in the development of PA programmes for the elderly. CONCLUSION: Based on experts' opinions, a self-assessment tool was found in order to access quality of PA programmes for the elderly. Information obtained with evaluations would be useful to organizations seeking to improve their services, customer satisfaction and, consequently, adherence to PA programmes, targeting the ageing population
Notes: DA - 20120214
IS - 1479-5868 (Electronic)
IS - 1479-5868 (Linking)
LA - eng
PT - Journal Article
PT - Research Support, Non-U.S. Gov't
SB - IM
	80. 	Marques AI, Soares P, Soares-Miranda L et al. Evaluation of physical activity programmes for the elderly - exploring the lessons from other sectors and examining the general characteristics of the programmes. BMC.Res.Notes 2011;4:368.
Ref ID: 114
Reprint: Not in File
Abstract: BACKGROUND: In Portugal, there are several physical activity (PA) programmes for elderly people developed by the local government. The importance of these programmes has been increasing since the evidence has shown that this type of health promotion interventions may reduce the deleterious effects of the ageing process. However, no study has already identified the general characteristics of these programmes nor if they use any scheme to assess the quality of the service provided. A widely-used scheme is the EFQM Excellence Model, which will be in the core of our present work. Thus, the main aims of this preliminary study were 1) to identify the general characteristics of the PA programmes developed by the Portuguese Local Public Administration 2) to determine the extent of implementation of quality initiatives in these programmes. METHODS: Data were collected by an on-line questionnaire sent to all Continental Municipalities (n = 278). Categorical data were expressed as absolute counts and percentages. Continuous data were expressed as the mean and SD. An open-ended question was analysed using qualitative content analysis with QSR NVivo software. Associations between categorical variables were tested by the use of contingency tables and the calculation of chi-square tests. Significance level was set at p </= 0.05. RESULTS: Results showed: i) a total of 125 PA programmes were identified in the 18 districts of the Portugal mainland; ii) the main goal of the majority (95.2%) was the participants' health promotion; iii) different characteristics of the programmes were found according to different regions of the country; iv) certain characteristics of the programmes were associated to the existence of other features; v) only one PA programme developed quality initiatives. CONCLUSIONS: In conclusion, although there are many PA programmes for elderly people spread throughout the country, aiming at improving the health of participants, the overwhelming majority does not adopt quality control initiatives. Considering that the quality of a service increases customer satisfaction, the continuous quality improvement of the PA programmes for elderly people should therefore be implemented since they can be useful and critical for elderly satisfaction and adherence
Notes: DA - 20111021
IS - 1756-0500 (Electronic)
IS - 1756-0500 (Linking)
LA - eng
PT - Journal Article
	81. 	Marques AI, Rosa MJ, Soares P, Santos R, Mota J, Carvalho J. Evaluation of physical activity programmes for elderly people - a descriptive study using the EFQM' criteria. BMC.Public Health 2011;11:123.
Ref ID: 124
Keywords: Aged/Exercise/Female/Health Promotion/standards/Humans/Interviews as Topic/Male/Middle Aged/Program Evaluation
Reprint: Not in File
Abstract: BACKGROUND: In the past years, there has been a growing concern in designing physical activity (PA) programmes for elderly people, because evidence suggests that such health promotion interventions may reduce the deleterious effects of the ageing process. Quality is an important issue when designing a PA programme for older people. Some studies support the Excellence Model of the European Foundation for Quality Management (EFQM) as an operational framework for evaluating the quality of an organization. Within this context, the aim of this study was to characterize the quality management models of the PA programmes developed by Portuguese Local Administration to enhance quality of life for elderly people, according to the criteria of the EFQM Excellence Model. METHODS: A methodological triangulation was conducted in 26 PA programmes using questionnaire surveys, semi-structured interviews and document analysis. We used standard approaches to the statistical analysis of data including frequencies and percentages for the categorical data. RESULTS: Results showed that Processes (65,38%), Leadership (61,03%), Customer results (58,46) and People (51,28%) had high percentage occurrences of quality practices. In contrast, Partnerships and resources (45,77%), People results (41,03%), Policy and strategy (37,91%), Key performance results (19,23%) and Society results (19,23%) had lower percentage occurrences. CONCLUSIONS: Our findings suggest that although there are some good practices in PA programmes, there are still relevant areas that require improvement
Notes: DA - 20110307
IS - 1471-2458 (Electronic)
IS - 1471-2458 (Linking)
LA - eng
PT - Journal Article
PT - Research Support, Non-U.S. Gov't
SB - IM
	82. 	Marques AI, Rosa MJ, Amorim M et al. Study protocol: using the Q-STEPS to assess and improve the quality of physical activity programmes for the elderly. BMC.Res.Notes 2012;5:171.
Ref ID: 101
Keywords: Aged/Exercise/Geriatric Assessment/Health Promotion/methods/Health Services for the Aged/standards/Humans/Program Evaluation/Quality Control/Quality of Life/Reproducibility of Results/Self-Assessment
Reprint: Not in File
Abstract: BACKGROUND: Aging is one of the most important and obvious phenomenon observed in our society. In the past years, there has been a growing concern in designing physical activity (PA) programmes for elderly people, because evidence suggests that such health promotion interventions may reduce the deleterious effects of the ageing process. Accordingly, a growing body of literature points to the importance of a sound approach to planning and evaluation in order to improve the quality of PA programmes. However, while numerous PA programmes have been designed for the elderly in recent years, their evaluation has been scarce. Quality management processes and tools provide a practical way for organisations to assess, identify and shed light on the areas requiring improvement. The Quality Self-assessment Tool for Exercise Programmes for Seniors (Q-STEPS) seems to provide a framework tailored to evaluate PA programmes for the elderly. FINDINGS: The primary purpose of this study is 1) to determine feasibility, acceptability and usability of the Q-STEPS. Secondary purposes of the study are: 2) to examine the quality of the PA programmes for elderly people developed by the Portuguese Local Administration over a three-year period of self-assessments in terms of: a) Enabler domains (Leadership, Policy and Strategy, People, Partnership and Resources, Processes); b) Result domains (Customer Results, People Results, Society Results and Key Performance Results); 3) to estimate the association between the use of Q-STEPS and some indicators relating to the elderly participants, during the three self-assessments, such as: attendance rates, physical fitness, health-related quality of life and the elderly's perceived quality of the programme. The study will be conducted in PA programmes for elderly adults from mainland Portuguese municipalities over a three-year period. The project will adopt a participative quality improvement approach that features annual learning cycles of: 1) self-assessment with the Q-STEPS; 2) feedback to and interpretation of results involving programme's staff; 3) action planning to achieve system changes; 4) implementation of strategies for change; and 5) review process through further self-assessment. The study will collect a range of process and outcome data that will be used to achieve the research aims. DISCUSSION: It is our understanding that the results of the Q-STEPS study will contribute directly to the evidence based on effectiveness of continuous quality improvement approaches, in order to improve customer satisfaction and adherence to PA programmes targeting the ageing population. This comprehensive evaluation will also add significant new knowledge regarding the characteristics associated with a sustainable public service
Notes: DA - 20120710
IS - 1756-0500 (Electronic)
IS - 1756-0500 (Linking)
LA - eng
PT - Journal Article
PT - Research Support, Non-U.S. Gov't
SB - IM
	83. 	Marsh AP, Chmelo EA, Katula JA, Mihalko SL, Rejeski WJ. Should physical activity programs be tailored when older adults have compromised function? J.Aging Phys.Act. 2009;17:294-306.
Ref ID: 145
Keywords: Age Factors/Aged/Aged,80 and over/Aging/physiology/Analysis of Variance/Disability Evaluation/Female/Humans/Male/Mobility Limitation/Motor Activity/Postural Balance/Program Development/Psychometrics/Risk Assessment/Risk Factors/Surveys and Questionnaires/Task Performance and Analysis/Walking
Reprint: Not in File
Abstract: The purpose of this study was to determine whether a walking program supplemented by tasks designed to challenge balance and mobility (WALK+) could improve physical function more than a traditional walking program (WALK) in older adults at risk for mobility disability. 31 community-dwelling older adults (M +/- SD age = 76 +/- 5 yr; Short Physical Performance Battery [SPPB] score = 8.4 +/- 1.7) were randomized to treatment. Both interventions were 18 sessions (1 hr, 3x/wk) and progressive in intensity and duration. Physical function was assessed using the SPPB and the 400-m-walk time. A subset of participants in the WALK group who had relatively lower baseline function showed only small improvement in their SPPB scores after the intervention (0.3 +/- 0.5), whereas a subset of participants in the WALK+ group with low baseline function showed substantial improvement in their SPPB scores (2.2 +/- 0.7). These preliminary data underscore the potential importance of tailoring interventions for older adults based on baseline levels of physical function
Notes: DA - 20091005
IS - 1063-8652 (Print)
IS - 1063-8652 (Linking)
LA - eng
PT - Comparative Study
PT - Journal Article
PT - Randomized Controlled Trial
SB - IM
	84. 	McClelland JW, Jayaratne KS, Bird C. Use of song as an effective teaching strategy for nutrition education in older adults. J.Nutr Gerontol.Geriatr. 2015;34:22-33.
Ref ID: 39
Keywords: Aged/Aged,80 and over/Education,Nonprofessional/methods/Elder Nutritional Physiological Phenomena/ethnology/Female/Food Assistance/Health Knowledge,Attitudes,Practice/Humans/Male/Music Therapy/North Carolina/Nutrition Policy/Nutritional Sciences/education/Patient Education as Topic/Poverty/Singing/United States/United States Department of Agriculture
Reprint: Not in File
Abstract: The objective of this study was to explore whether singing an educational song would be effective in improving older adults' knowledge about nutrition. We used a randomized controlled design to determine whether singing an educational song would result in increased nutrition knowledge in a low-income population of older adults compared to a control group of similar adults who did not sing the song. Eighteen congregate nutrition sites were randomly assigned to the treatment or control group. Analysis via independent samples t -test showed the knowledge gain mean scores for the treatment group were significantly ( P < 0.05) greater than those of the control group. This study supports a unique new approach to increasing nutrition knowledge of older adults by using music
Notes: DA - 20150325
IS - 2155-1200 (Electronic)
LA - eng
PT - Journal Article
PT - Multicenter Study
PT - Randomized Controlled Trial
PT - Research Support, U.S. Gov't, Non-P.H.S
SB - IM
SB - N
	85. 	McKay DL, Houser RF, Blumberg JB, Goldberg JP. Nutrition information sources vary with education level in a population of older adults. J.Am.Diet.Assoc. 2006;106:1108-11.
Ref ID: 176
Keywords: Aged/Aged,80 and over/Boston/Diet/psychology/standards/Eating/Educational Status/Female/Health Behavior/Humans/Male/Mass Media/Middle Aged/Nutritional Sciences/education/Socioeconomic Factors
Reprint: Not in File
Abstract: Education level, more than any other socioeconomic factor, can predict disease risk, health behavior patterns, and diet quality. It has been suggested that one reason higher education promotes more healthful diets is that better-educated people may get better nutrition information. We present data from a survey examining specific sources of nutrition information among an older adult population (age >50 years, n=176), and compare the difference in sources associated with extent of education. Reliance on doctors, television, and neighbors was significantly higher among those with less education (P<0.05). Our results also show that less-educated older adults rely more upon different specific sources for their nutrition information than those who have attained a higher level of education. Strategies to improve and/or ensure the quality of the specific nutrition information sources this vulnerable group relies on may be needed
Notes: DA - 20060703
IS - 0002-8223 (Print)
IS - 0002-8223 (Linking)
LA - eng
PT - Journal Article
SB - AIM
SB - IM
	86. 	McMahon SK, Wyman JF, Belyea MJ, Shearer N, Hekler EB, Fleury J. Combining Motivational and Physical Intervention Components to Promote Fall-Reducing Physical Activity Among Community-Dwelling Older Adults: A Feasibility Study. Am.J.Health Promot. 2015.
Ref ID: 22
Reprint: Not in File
Abstract: Purpose . To assess the feasibility of a new intervention, Ready~Steady, in terms of demand, acceptability, implementation, and limited efficacy. Design . Randomized controlled trial; repeated measures. Setting . Two rural communities in Itasca County, Minnesota. Subjects . Thirty participants were randomized to an intervention (n = 16) or attention-control (n = 14) group. Intervention . Ready~Steady combined two components: (1) motivational (motivational support, social network support, empowering education), and (2) fall-reducing physical activities (PAs; guidance to practice leg-strengthening, balance, and flexibility activities and walking). Measures . Acceptability questionnaire and Indices of Procedural Consistency (investigator developed), Community Health Activity Model Program for Seniors Questionnaire (confirmed with accelerometry), Short Physical Performance Battery, Perceived Environmental Support Scale, Social Support for Exercise Questionnaire, Goal Attainment Scale, Index of Readiness, and Index of Self-Regulation. Analysis . Descriptive statistics and a marginal approach to repeated-measures analysis of variance, using mixed-model procedures. Results . Attrition was 7% and mean attendance was 7.2 of 8 sessions, participants evaluated Ready~Steady as acceptable, and implementation fidelity was good. The intervention group improved significantly more than the attention-control group in PA behavior, F1,27 = 11.92, p = .002; fall risk (functional balance and strength), F1,27 = 14.89, p = .001; support for exercise from friends, F1,27 = 11.44, p = .002; and self-regulation, F1,26 = 38.82, p < .005. Conclusion . The Ready~Steady intervention was feasible as evidenced by low attrition and good attendance and implementation, as well as positive effects on targeted outcomes and theoretical mechanisms of change
Notes: DA - 20150921
IS - 2168-6602 (Electronic)
IS - 0890-1171 (Linking)
LA - ENG
PT - JOURNAL ARTICLE
	87. 	Mitchell RE, Ash SL, McClelland JW. Nutrition education among low-income older adults: a randomized intervention trial in Congregate Nutrition sites. Health Educ.Behav. 2006;33:374-92.
Ref ID: 180
Keywords: Aged/Aged,80 and over/Dietary Supplements/Female/Health Surveys/Humans/Male/North Carolina/Nutritional Sciences/education/Nutritional Status/Poverty/Prospective Studies
Reprint: Not in File
Abstract: Nutritional well-being among older adults is critical for maintaining health, increasing longevity, and decreasing the impact of chronic illness. However, few well-controlled studies have examined nutritional behavior change among low-income older adults. A prospective, controlled, randomized design examined a five session nutrition education module delivered to limited-resource older adults (N = 703) in Congregate Nutrition sites by Cooperative Extension agents. Experimental group participants were significantly more likely than control group participants to increase multivitamin use, to increase calcium supplement use, to read labels of dietary supplements, to carry a supplement and/or medication list, and to discuss such use with their health care professional. The study addresses weaknesses in the literature by using a theoretically derived education component, implementing the intervention within a setting regularly used by low-income older adults, employing randomized assignment to intervention and control conditions, and using hierarchical linear modeling to deal with "nested" data
Notes: DA - 20060515
IS - 1090-1981 (Print)
IS - 1090-1981 (Linking)
LA - eng
PT - Journal Article
PT - Randomized Controlled Trial
SB - IM
	88. 	Moore-Harrison TL, Speer EM, Johnson FT, Cress ME. The effects of aerobic training and nutrition education on functional performance in low socioeconomic older adults. J.Geriatr.Phys.Ther. 2008;31:18-23.
Ref ID: 161
Keywords: Aged/Aged,80 and over/Exercise Therapy/methods/Exercise Tolerance/Female/Food Habits/Geriatric Assessment/Health Education/Health Promotion/Humans/Male/Nutrition Assessment/Physical Fitness/physiology/Poverty Areas/Walking
Reprint: Not in File
Abstract: PURPOSE: To describe the population in terms of risk for disability and compare the effects of a walking intervention and nutrition education intervention on risk modification and functional performance in lower socioeconomic older adults using a randomized controlled study. METHODS: Twenty-six community-dwelling older adults aged 60 and older were randomly assigned to a 16-week walking exercise group or a nutrition education control group. Peak aerobic capacity and physical function were measured at baseline and post intervention. Physical function was measured using the Medical Outcomes Study Short Form Health Survey Physical Function subscale, Short Physical Performance Battery, Physical Performance Test, and Continuous Scale Physical Functional Performance 10 item test (CS-PFP10). RESULTS: Eighty-five percent of the participants were at risk for preclinical disability of which 50% were at risk for moderate disability. The walking exercise group significantly improved in peak aerobic capacity (18.9%), physical function (25%) using the CS-PFP10 compared to the control group. CONCLUSION: These findings highlight the importance of physical activity and indicate that walking, a simple exercise that can be done without specialized exercise leader or equipment can significantly increase peak aerobic capacity and physical function in just 4 months
Notes: DA - 20080520
IS - 1539-8412 (Print)
IS - 1539-8412 (Linking)
LA - eng
PT - Journal Article
PT - Randomized Controlled Trial
SB - IM
	89. 	Morey MC, Pieper CF, Edelman DE et al. Enhanced fitness: a randomized controlled trial of the effects of home-based physical activity counseling on glycemic control in older adults with prediabetes mellitus. J.Am.Geriatr.Soc. 2012;60:1655-62.
Ref ID: 91
Keywords: Aged/Aged,80 and over/Aging/physiology/Anthropometry/Blood Glucose/analysis/Body Mass Index/Counseling/Female/Hemoglobin A,Glycosylated/Homeostasis/Humans/Insulin Resistance/Male/Middle Aged/Motor Activity/Physical Fitness/Prediabetic State/physiopathology/prevention & control/Quality of Life/Time Factors/Treatment Outcome/Veterans
Reprint: Not in File
Abstract: OBJECTIVES: To determine whether a home-based multicomponent physical activity counseling (PAC) intervention is effective in reducing glycemic measures in older outpatients with prediabetes mellitus. DESIGN: Controlled clinical trial. SETTING: Primary care clinics of the Durham Veterans Affairs (VA) Medical Center between September 29, 2008, and March 25, 2010. PARTICIPANTS: Three hundred two overweight (body mass index 25-45 kg/m(2) ), older (60-89) outpatients with impaired glucose tolerance (fasting blood glucose 100-125 mg/dL, glycosylated hemoglobin (HbA1c) <7%) randomly assigned to a PAC intervention group (n = 180) or a usual care control group (n = 122). INTERVENTION: A 12-month, home-based multicomponent PAC program including one in-person baseline counseling session, regular telephone counseling, physician endorsement in clinic with monthly automated encouragement, and customized mailed materials. All study participants, including controls, received a consultation in a VA weight management program. MEASUREMENTS: The primary outcome was a homeostasis model assessment of insulin resistance (HOMA-IR), calculated from fasting insulin and glucose levels at baseline and 3 and 12 months. HbA1c was the secondary indicator of glycemic control. Other secondary outcomes were anthropometric measures and self-reported physical activity, health-related quality of life, and physical function. RESULTS: There were no significant differences between the PAC and control groups over time for any of the glycemic indicators. Both groups had small declines over time of approximately 6% in fasting blood glucose (P < .001), and other glycemic indicators remained stable. The declines in glucose were not sufficient to affect the change in HOMA-IR scores due to fluctuations in insulin over time. Endurance physical activity increased significantly in the PAC group (P < .001) and not in the usual care group. CONCLUSION: Home-based telephone counseling increased physical activity levels but was insufficient to improve glycemic indicators in older outpatients with prediabetes mellitus
Notes: DA - 20120918
IS - 1532-5415 (Electronic)
IS - 0002-8614 (Linking)
LA - eng
PT - Controlled Clinical Trial
PT - Journal Article
PT - Randomized Controlled Trial
PT - Research Support, N.I.H., Extramural
PT - Research Support, U.S. Gov't, Non-P.H.S
RN - 0 (Blood Glucose)
RN - 0 (Hemoglobin A, Glycosylated)
SB - IM
	90. 	Motl RW, Konopack JF, McAuley E, Elavsky S, Jerome GJ, Marquez DX. Depressive symptoms among older adults: long-term reduction after a physical activity intervention. J.Behav.Med. 2005;28:385-94.
Ref ID: 187
Keywords: Aged/Depression/diagnosis/epidemiology/therapy/Female/Humans/Male/Middle Aged/Motor Activity/Self Concept/Surveys and Questionnaires/Walking
Reprint: Not in File
Abstract: We examined the effects of two physical activity modes on depressive symptoms over a 5-year period among older adults and change in physical self-esteem as a mediator of changes in depressive symptoms. Formerly sedentary, older adults (N = 174) were randomly assigned into 6-month conditions of either walking or low-intensity resistance/flexibility training. Depressive symptoms and physical self-esteem were measured before and after the 6-month intervention, and 12 and 60 months after intervention initiation. Depressive symptoms scores were decreased immediately after the intervention, followed by a sustained reduction for 12 and 60 months after intervention initiation; there was no differential pattern of change between the physical activity modes. Change in physical self-esteem predicted change in depressive symptoms. This study supports the effectiveness of an exercise intervention for the sustained reduction of depressive symptoms among sedentary older adults and physical self-esteem as a potential mediator of this effect
Notes: DA - 20051026
IS - 0160-7715 (Print)
IS - 0160-7715 (Linking)
LA - eng
PT - Journal Article
PT - Randomized Controlled Trial
PT - Research Support, N.I.H., Extramural
SB - IM
	91. 	Mouton A, Cloes M. Efficacy of a web-based, center-based or combined physical activity intervention among older adults. Health Educ.Res. 2015;30:422-35.
Ref ID: 40
Reprint: Not in File
Abstract: With more social support and environment-centered interventions being recommended in web-based interventions, this study examined the efficacy of three intervention conditions aimed at promoting physical activity (PA) in older adults. The efficacy analyses included the self-reported PA level, stage of change for PA and awareness about PA among participants. Eligible participants (N = 149; M = 65 years old, SD = 6), recruited in a unique Belgian French-speaking municipality, were randomized in four research arms for a 3-month intervention: (i) web-based; (ii) center-based; (iii) mixed (combination of web- and center-based); and (iv) control (no intervention). Web-based condition included a PA website and monthly tailored emails whereas center-based condition comprised 12 sessions (1 per week) of group exercising. With a significant increase in PA, the PA stage of change and the PA awareness at 12 months, the mixed intervention condition seemed to include the key social and motivating elements for sustainable behavior change. Center-based intervention was more likely to produce significant improvements of the PA level and the stage of change for PA change whereas web-based intervention was more likely to extend the awareness about PA
Notes: DA - 20150519
IS - 1465-3648 (Electronic)
IS - 0268-1153 (Linking)
LA - eng
PT - Journal Article
SB - T
	92. 	Nguyen HQ, Koepsell T, Unutzer J, Larson E, LoGerfo JP. Depression and use of a health plan-sponsored physical activity program by older adults. Am.J.Prev.Med. 2008;35:111-7.
Ref ID: 157
Keywords: Aged/Case-Control Studies/Comorbidity/Depression/classification/therapy/Exercise/Female/Fitness Centers/statistics & numerical data/Health Services for the Aged/economics/organization & administration/Humans/International Classification of Diseases/Male/Medicare/Patient Participation/United States/Washington
Reprint: Not in File
Abstract: BACKGROUND: The purpose of this study was to determine the associations between depression and the likelihood of enrollment in a health plan-sponsored physical activity program and pattern of program participation over 2 years; a secondary aim was to examine the association between participation dose and depression risk. There are no published studies on how depression influences participation in health plan-sponsored physical activity programs and how participation affects depression risk in older adults in nonresearch settings. METHODS: This study used administrative data from a Medicare Advantage plan. Participants (n=4766) were enrolled in the plan for at least 1 year prior to participating in the plan-sponsored health club benefit (Silver Sneakers). Controls were age- and gender-matched to participants (n=9035). Members were identified as having depression based on ICD-9-CM codes. Multivariate regression and generalized estimating equations models were used. Data were collected between 1998 and 2003 and analyzed in 2008. RESULTS: Members who had a history of depression were as likely to participate in Silver Sneakers as nondepressed members (OR: 1.03; 95% CI=0.89, 1.20; p=0.67). The risk of lapse in Silver Sneakers attendance was 28%-55% (p<0.05) higher for depressed participants during months 15-24. For nondepressed Silver Sneakers participants, attendance of at least 2 visits/week during Year 1 was significantly associated with lower risk of depression in Year 2 (OR=0.54; 95% CI=0.37, 0.79; p=0.002); a similar but statistically nonsignificant association was observed for previously depressed participants (OR=0.51; 95% CI=0.26, 1.02; p=0.06). CONCLUSIONS: While depressed older adults are as likely to enroll in a health plan-sponsored physical activity as nondepressed members, they were at higher risk of attendance lapses. Greater participation in the physical activity program was associated with lower depression risk
Notes: DA - 20080711
IS - 0749-3797 (Print)
IS - 0749-3797 (Linking)
LA - eng
PT - Journal Article
PT - Research Support, N.I.H., Extramural
PT - Research Support, Non-U.S. Gov't
PT - Research Support, U.S. Gov't, P.H.S
SB - IM
	93. 	Olson EA, McAuley E. Impact of a brief intervention on self-regulation, self-efficacy and physical activity in older adults with type 2 diabetes. J.Behav.Med. 2015;38:886-98.
Ref ID: 29
Reprint: Not in File
Abstract: Despite evidence of the benefits of physical activity, most individuals with type 2 diabetes do not meet physical activity recommendations. The purpose of this study was to test the efficacy of a brief intervention targeting self-efficacy and self-regulation to increase physical activity in older adults with type 2 diabetes. Older adults (Mage = 61.8 +/- 6.4) with type 2 diabetes or metabolic syndrome were randomized into a titrated physical activity intervention (n = 58) or an online health education course (n = 58). The intervention included walking exercise and theory-based group workshops. Self-efficacy, self-regulation and physical activity were assessed at baseline, post-intervention, and a follow-up. Results indicated a group by time effect for self-regulation [F(2,88) = 14.021, p < .001, eta (2) = .24] and self-efficacy [F(12,77) = 2.322, p < .05, eta (2) = .266] with increases in the intervention group. The intervention resulted in short-term increases in physical activity (d = .76, p < .01), which were partially maintained at the 6-month follow-up (d = .35, p < .01). The intervention increased short-term physical activity but was not successful at maintaining increases in physical activity. Similar intervention effects were observed in self-efficacy and self-regulation. Future research warrants adjusting intervention strategies to increase long-term change
Notes: DA - 20151031
IS - 1573-3521 (Electronic)
IS - 0160-7715 (Linking)
LA - eng
PT - Journal Article
PT - Research Support, N.I.H., Extramural
PT - Research Support, Non-U.S. Gov't
SB - IM
	94. 	Opdenacker J, Delecluse C, Boen F. The longitudinal effects of a lifestyle physical activity intervention and a structured exercise intervention on physical self-perceptions and self-esteem in older adults. J.Sport Exerc.Psychol. 2009;31:743-60.
Ref ID: 136
Keywords: Aged/Analysis of Variance/Belgium/Body Image/Case-Control Studies/Exercise/psychology/Female/Humans/Life Style/Longitudinal Studies/Male/Middle Aged/Models,Theoretical/Motor Activity/Self Concept
Reprint: Not in File
Abstract: The objectives of this study were (1) to evaluate the long-term effects of a lifestyle physical activity intervention (n = 60) and a structured exercise intervention (n = 60) on physical self-perceptions and self-esteem in older adults compared with a control group (n = 66), and (2) to test the longitudinal fit of the exercise and self-esteem model (EXSEM). Immediately after the 11-month interventions, the lifestyle group showed significant improvements in self-perceived physical condition, sport competence, body attractiveness, and physical self-worth. In the structured group, significant effects were found on physical condition and sport competence. One year later, the lifestyle program had significant effects on body attractiveness and global self-esteem, while the structured group showed significant improvements in physical condition, sport competence, and body attractiveness. Path analyses revealed a good fit for the EXSEM across the 2-year period
Notes: DA - 20100413
IS - 0895-2779 (Print)
IS - 0895-2779 (Linking)
LA - eng
PT - Journal Article
SB - IM
	95. 	Pahor M, Blair SN, Espeland M et al. Effects of a physical activity intervention on measures of physical performance: Results of the lifestyle interventions and independence for Elders Pilot (LIFE-P) study. J.Gerontol.A Biol.Sci.Med.Sci. 2006;61:1157-65.
Ref ID: 174
Keywords: Activities of Daily Living/Aged/Aged,80 and over/Disability Evaluation/Exercise/Female/Follow-Up Studies/Geriatric Assessment/Health Behavior/Health Education/Health Promotion/Humans/Life Style/Male/Pilot Projects/Single-Blind Method/Walking
Reprint: Not in File
Abstract: BACKGROUND: The Short Physical Performance Battery (SPPB), which includes walking, balance, and chair stands tests, independently predicts mobility disability and activities of daily living disability. To date, however, there is no definitive evidence from randomized controlled trials that SPPB scores can be improved. Our objective was to assess the effect of a comprehensive physical activity (PA) intervention on the SPPB and other physical performance measures. METHODS: A total of 424 sedentary persons at risk for disability (ages 70-89 years) were randomized to a moderate-intensity PA intervention or a successful aging (SA) health education intervention and were followed for an average of 1.2 years. RESULTS: The mean baseline SPPB score on a scale of 0-12, with 12 corresponding to highest performance, was 7.5. At 6 and 12 months, the PA versus SA group adjusted SPPB (+/- standard error) scores were 8.7 +/- 0.1 versus 8.0 +/- 0.1, and 8.5 +/- 0.1 versus 7.9 +/- 0.2, respectively (p < .001). The 400-meter walking speed was also significantly improved in the PA group. The PA group had a lower incidence of major mobility disability defined as incapacity to complete a 400-meter walk (hazard ratio = 0.71, 95% confidence interval = 0.44-1.20). CONCLUSIONS: A structured PA intervention improved the SPPB score and other measures of physical performance. An intervention that improves the SPPB performance may also offer benefit on more distal health outcomes, such as mobility disability
Notes: DA - 20061214
IS - 1079-5006 (Print)
IS - 1079-5006 (Linking)
LA - eng
PT - Journal Article
PT - Multicenter Study
PT - Randomized Controlled Trial
PT - Research Support, N.I.H., Extramural
PT - Research Support, N.I.H., Intramural
PT - Research Support, U.S. Gov't, Non-P.H.S
SB - AIM
SB - IM
	96. 	Pasalich M, Lee AH, Jancey J, Burke L, Howat P. Sustainability of a physical activity and nutrition program for seniors. J.Nutr Health Aging 2013;17:486-91.
Ref ID: 76
Keywords: Aged/Body Mass Index/Data Collection/Diet/Dietary Fats/administration & dosage/Dietary Fiber/Exercise/Female/Follow-Up Studies/Food Habits/Health Behavior/Health Promotion/methods/Humans/Interviews as Topic/Male/Middle Aged/Patient Compliance/Prospective Studies/Sex Factors/Surveys and Questionnaires/Time Factors/Waist-Hip Ratio/Weight Loss/Western Australia
Reprint: Not in File
Abstract: OBJECTIVE: This prospective cohort study aimed to determine the impact of a low cost, home-based physical activity and nutrition program for older adults at 6 months follow-up. DESIGN: A follow-up survey was conducted 6 months after program completion via computer-assisted telephone interviewing. The International Physical Activity Questionnaire and the Fat and Fibre Barometer were used to measure physical activity levels and dietary behaviours, respectively. Self-reported height, weight, waist and hip circumferences were obtained. Changes over three time points of data collection (baseline, post-program, follow-up) and differences between the intervention and control groups were assessed. The use of program materials was also evaluated. SETTING: Community and home-based. PARTICIPANTS: Insufficiently active 60 to 70 year olds (n = 176, intervention and n = 198, control) residing in suburbs within the Perth metropolitan area. RESULTS: A sustained improvement was observed for the intervention group in terms of fat avoidance behaviours (p interaction = .007). Significant improvements were found for strength exercises, fibre intake, body mass index and waist-to-hip ratio at either post-program or follow-up, however the overall effect was not significant. At post-program, the intervention group increased time spent participating in moderate activity by 50 minutes (p > .05), which was followed by a significant decline at follow-up (p < .05). Among intervention group participants, males and females differed with respect to strength exercises and moderate physical activity. CONCLUSION: This low-cost physical activity and nutrition intervention resulted in a sustained improvement in fat avoidance behaviours and overall short-term gains in physical activity. Future studies for older adults are recommended to investigate gender-specific behavioural barriers as well as booster interventions which focus on physical activity
Notes: DA - 20130502
IS - 1760-4788 (Electronic)
IS - 1279-7707 (Linking)
LA - eng
PT - Evaluation Studies
PT - Journal Article
PT - Research Support, Non-U.S. Gov't
RN - 0 (Dietary Fats)
SB - IM
	97. 	Patel A, Keogh JW, Kolt GS, Schofield GM. The long-term effects of a primary care physical activity intervention on mental health in low-active, community-dwelling older adults. Aging Ment.Health 2013;17:766-72.
Ref ID: 79
Keywords: Aged/Aged,80 and over/Analysis of Variance/Depression/therapy/Exercise/psychology/Female/Follow-Up Studies/Humans/Male/Mental Health/Primary Health Care/methods/Program Evaluation/Psychiatric Status Rating Scales/Residence Characteristics/Walking
Reprint: Not in File
Abstract: OBJECTIVES: To examine the effect that physical activity delivered via two different versions of the Green Prescription (a primary care physical activity scripting program) had on depressive symptomatology and general mental health functioning over a 12-month period in non-depressed, low-active, community-dwelling older adults. METHOD: Two hundred and twenty-five participants from the Healthy Steps study took part in the present study. Healthy Steps participants were randomized to receive either the standard time-based or a modified pedometer-based Green Prescription. Depression, mental health functioning and physical activity were measured at baseline, post-intervention (3 months post-baseline) and at the 9-month follow-up period. RESULTS: At post-intervention, a positive association was found between increases in leisure-time physical activity and total walking physical activity and a decrease in depressive symptomatology (within the non-depressed range of the GDS-15) and an increase in perceived mental health functioning, regardless of intervention allocation. These improvements were also evident at the follow-up period for participants in both intervention allocation groups. CONCLUSION: Our findings suggest that the standard time-based Green Prescription and a modified pedometer-based Green Prescription are both effective in maintaining and improving mental health in non-depressed, previously low-active older adults
Notes: DA - 20130724
IS - 1364-6915 (Electronic)
IS - 1360-7863 (Linking)
LA - eng
PT - Journal Article
PT - Randomized Controlled Trial
SB - IM
	98. 	Payette H. Nutrition as a determinant of functional autonomy and quality of life in aging: a research program. Can.J.Physiol Pharmacol. 2005;83:1061-70.
Ref ID: 185
Keywords: Activities of Daily Living/Aged/Aged,80 and over/Aging/Canada/Frail Elderly/Humans/Nutritional Status/Quality of Life/Research
Reprint: Not in File
Abstract: With the aging of the Canadian population, functional autonomy and quality of life among seniors are now important public health issues. We hypothesized that nutrition is an important determinant of the quality of aging because of its potential to modulate the transitions from vulnerability to frailty and dependence. Over the past 15 years, our research program addressed the prevalence, the determinants, and the consequences of undernutrition among seniors, especially the free-living frail elderly. Very low energy and nutrient intakes were observed as well as a high prevalence of involuntary weight loss. These chronic conditions were associated with early institutionalization and increased mortality rates. Intervention strategies were then developed and evaluated, including the Nutrition Screening Program and the Nutrition Support Program. The effectiveness of these programs was shown with respect to improvement of nutritional status. However, this improvement was not sufficient to produce significant changes in functional autonomy or quality of life. Methodological issues related to the conduct of intervention studies in this specific population were addressed. A conceptual framework of nutritional intervention is currently being validated. A large longitudinal study that is being undertaken will further contribute to our understanding of the aging process as determined by a modifiable factor such as nutrition
Notes: DA - 20060104
IS - 0008-4212 (Print)
IS - 0008-4212 (Linking)
LA - eng
PT - Journal Article
PT - Review
SB - IM
	99. 	Peels DA, Bolman C, Golsteijn RH et al. Long-term efficacy of a printed or a Web-based tailored physical activity intervention among older adults. Int.J.Behav.Nutr Phys.Act. 2013;10:104.
Ref ID: 60
Keywords: Aged/Cluster Analysis/Female/Follow-Up Studies/Health Behavior/Health Promotion/methods/Humans/Internet/Male/Middle Aged/Motor Activity/physiology/Socioeconomic Factors/Surveys and Questionnaires/Time
Reprint: Not in File
Abstract: BACKGROUND: This study provides insight into the long-term efficacy (i.e. 12 month results) of the Web-based or print-delivered tailored Active Plus intervention (with and without environmental approach) to promote physical activity (PA) among the over-fifties. Differences in effect among subgroups are studied as well. METHODS: Intervention groups (i.e. print-delivered basic (PB; N = 439), print-delivered environmental (PE; N = 435), Web-based basic (WB; N = 423), Web-based environmental (WE; N = 432)) and a waiting list control group (N = 411) were studied in a clustered randomized controlled trial. Intervention participants received tailored advice three times within 4 months. Long-term effects (12 months after the intervention has started, i.e. 8 months after the intervention was completed) on PA (i.e. self-reported weekly minutes and days of sufficient PA) were tested using multilevel linear regression analyses. Participants' age, gender, BMI, educational level, PA intention and the presence of a chronic physical limitation were considered to be potential moderators of the effect. RESULTS: Overall, the Active Plus intervention was effective in increasing weekly days of sufficient PA (B=0.49; p=.005), but ineffective in increasing weekly minutes of PA (B=84.59; p=.071). Per intervention condition analysis showed that the PB-intervention (B(days)=0.64; p=.002; B(min)=111.36; p=.017) and the PE-intervention (B(days)=0.70; p=.001; B(min)=157.41; p=.001) were effective in increasing days and minutes of PA. Neither Web-based conditions significantly increased PA, while the control group decreased their PA. In contrast to the intervention effect on minutes of PA, the effect on weekly days of PA was significantly moderated by the participants' baseline intention to be sufficiently physically active. CONCLUSIONS: In general, after 12 months the print-delivered interventions resulted in stronger effects than the Web-based interventions. The participants' baseline intention was the only significant moderator of the intervention effect. All other assessed user characteristics did not significantly moderate the effect of the intervention, which might indicate that the intervention is sufficiently tailored to the different participant characteristics. Additional efforts should be taken to increase the sustainability of Web-based interventions. TRIAL REGISTRATION: Dutch Trial Register: NTR2297
Notes: DA - 20131115
IS - 1479-5868 (Electronic)
IS - 1479-5868 (Linking)
LA - eng
PT - Journal Article
PT - Randomized Controlled Trial
PT - Research Support, Non-U.S. Gov't
SB - IM
	100. 	Pogge EK, Eddings L. Effect of a 12-week nutrition and wellness program in independent living seniors. J.Nutr Educ.Behav. 2013;45:471-2.
Ref ID: 80
Keywords: Age Factors/Aged/Aged,80 and over/Blood Pressure/Body Mass Index/Body Weight/Health Knowledge,Attitudes,Practice/Health Promotion/methods/Humans/Independent Living/Nutritional Sciences/education
Reprint: Not in File
Notes: DA - 20130910
IS - 1878-2620 (Electronic)
IS - 1499-4046 (Linking)
LA - eng
PT - Journal Article
PT - Research Support, Non-U.S. Gov't
SB - IM
	101. 	Porter KN, Fischer JG, Johnson MA. Improved physical function and physical activity in older adults following a community-based intervention: Relationships with a history of depression. Maturitas 2011;70:290-4.
Ref ID: 115
Keywords: Activities of Daily Living/psychology/Aged/Aged,80 and over/Depression/Depressive Disorder/Exercise/Exercise Test/Female/Georgia/Humans/Male/Middle Aged/Physical Exertion/Physical Fitness/Residence Characteristics/Treatment Outcome/United States
Reprint: Not in File
Abstract: The purpose of this study was to explore the relationship of a history of depression with moderate physical activity and physical function before and after a physical activity intervention of congregate meal participants in senior centers from all 12 Georgia Area Agencies on Aging (AAA). Participants were a convenience sample of older adults (n=376, mean age=76 years, 82% female, 64% Caucasian, 36% African American, 22% a history of depression). The physical activity intervention included educator-led chair exercises that incorporated balls and bands. Pre- and post-tests assessed moderate physical activity and physical function. At the pre-test, a history of depression was not related to moderate physical activity or physical function. Following the intervention there were significant increases in both moderate physical activity and physical function, but a history of depression was a negative predictor of improvements in physical activity when controlled for site, demographics, and health-related conditions. These results provide an evidence base for the effectiveness of this intervention in improving moderate physical activity and physical function in a community setting, but additional efforts may be needed to improve the impact of this type of intervention among older adults with a history of depression
Notes: DA - 20111007
IS - 1873-4111 (Electronic)
IS - 0378-5122 (Linking)
LA - eng
PT - Clinical Trial
PT - Journal Article
PT - Research Support, Non-U.S. Gov't
SB - IM
	102. 	Poulin MJ, Eskes GA, Hill MD. Physical Activity vs Health Education for Cognition in Sedentary Older Adults. JAMA 2016;315:415.
Ref ID: 13
Keywords: Dementia/prevention & control/Executive Function/Exercise Therapy/methods/Female/Health Promotion/Humans/Male/Mild Cognitive Impairment/Sedentary Lifestyle
Reprint: Not in File
Notes: DA - 20160127
IS - 1538-3598 (Electronic)
IS - 0098-7484 (Linking)
LA - eng
PT - Comment
PT - Letter
SB - AIM
SB - IM
	103. 	Puranen T, Finne-Soveri H, Auranne K, Lehtinen-Fraser M, Suominen MH. Nutritional Intervention Via Videoconferencing for Older Adults Receiving Home Care - A Pilot Study. J.Frailty.Aging 2013;2:33-7.
Ref ID: 87
Reprint: Not in File
Abstract: BACKGROUND: Malnutrition is common in aged home care clients and that affects negatively the health of aged people. Nutritional screening is recommended for early detection of malnutrition. OBJECTIVES: The aim was to assess the nutritional status and food intake of home care receivers and improve their nutrient intake with tailored nutritional advice administered via videoconferencing. DESIGN: Intervention with follow-up. SETTING: Home care in the city of Helsinki. PARTICIPANTS: 25 older (>65 years) adults receiving home care. INTERVENTION: After an initial assessment determining their needs, participants received tailored nutritional advice via videoconferencing over a six-month follow-up period. MEASUREMENTS: Participants nutritional status was assessed with a Mini Nutritional Assessment -test (MNA). Nutrient intake was calculated based on a detailed three-day food diary compiled twice during the six-month follow-up period. RESULTS: Altogether 25 persons participated in the study (mean age 78.5 years, 88 % females). According to the MNA test 80 % were at risk of malnutrition at the outset. Energy (1329 kcal) and mean nutrient intakes of protein (54 g) and folic acid (210 mug), for example, were inadequate. After six months of intervention, the mean energy intake had increased to 1450 (SD 319) kcal, protein to 65 (SD 20) g, and folic acid to 231 (SD 105) mug per day. CONCLUSIONS: The energy, protein and other nutrient intake of the study participants increased during the six-month intervention. Videoconferencing seemed to be a well-accepted and feasible method for providing nutritional advice to older home care clients
Notes: DA - 20160413
IS - 2260-1341 (Print)
IS - 2260-1341 (Linking)
LA - eng
PT - Journal Article
	104. 	Purath J, Keller CS, McPherson S, Ainsworth B. A randomized controlled trial of an office-based physical activity and physical fitness intervention for older adults. Geriatr.Nurs. 2013;34:204-11.
Ref ID: 78
Keywords: Aged/Energy Metabolism/Humans/Motor Activity/Office Visits/Physical Fitness/Primary Health Care/organization & administration/Self Efficacy/Social Support
Reprint: Not in File
Abstract: This primary care-based study aimed to evaluate the efficacy and feasibility of a 24-week intervention on physical activity and physical fitness in a group of community-dwelling older adults. Secondary aims were to determine the effect of the intervention on self-efficacy and barriers to physical activity. Intervention participants (n = 36) received an exercise prescription based on physical fitness test results and personal choice. Comparison participants (n = 36) received a nutrition intervention. Both groups received 10 follow-up telephone calls. Repeated measures ANOVA analyses showed no direct effects of the intervention on the primary outcomes of physical activity or physical fitness in the intervention group (p > 0.05). Secondary analyses with ANCOVA that included potential moderating variables of age, gender, income, BMI, and support for physical activity showed that the intervention group significantly increased frequency of all physical activity (F = 3.50, p < 0.05) as well as the fitness outcomes of lower body strength (F = 3.63, p < 0.05) and aerobic endurance (F = 4.03, p < 0.05). This is one of the first studies to evaluate the use of fitness measures to increase physical activity and fitness in the primary care setting. The intervention improved some aspects of physical activity and fitness for selected participants
Notes: DA - 20130603
IS - 1528-3984 (Electronic)
IS - 0197-4572 (Linking)
LA - eng
PT - Journal Article
PT - Randomized Controlled Trial
SB - N
	105. 	Rejeski WJ, Axtell R, Fielding R et al. Promoting physical activity for elders with compromised function: the lifestyle interventions and independence for elders (LIFE) study physical activity intervention. Clin.Interv.Aging 2013;8:1119-31.
Ref ID: 65
Keywords: Accelerometry/statistics & numerical data/Aged/Aged,80 and over/Exercise/Female/Health Promotion/methods/organization & administration/Humans/Independent Living/Male/Mobility Limitation/Qualitative Research/Risk Reduction Behavior/Sex Distribution
Reprint: Not in File
Abstract: The Lifestyle Interventions and Independence for Elders (LIFE) Study is a Phase III randomized controlled clinical trial (Clinicaltrials.gov identifier: NCT01072500) that will provide definitive evidence regarding the effect of physical activity (PA) on major mobility disability in older adults (70-89 years old) who have compromised physical function. This paper describes the methods employed in the delivery of the LIFE Study PA intervention, providing insight into how we promoted adherence and monitored the fidelity of treatment. Data are presented on participants' motives and self-perceptions at the onset of the trial along with accelerometry data on patterns of PA during exercise training. Prior to the onset of training, 31.4% of participants noted slight conflict with being able to meet the demands of the program and 6.4% indicated that the degree of conflict would be moderate. Accelerometry data collected during PA training revealed that the average intensity - 1,555 counts/minute for men and 1,237 counts/minute for women - was well below the cutoff point used to classify exercise as being of moderate intensity or higher for adults. Also, a sizable subgroup required one or more rest stops. These data illustrate that it is not feasible to have a single exercise prescription for older adults with compromised function. Moreover, the concept of what constitutes "moderate" exercise or an appropriate volume of work is dictated by the physical capacities of each individual and the level of comfort/stability in actually executing a specific prescription
Notes: DA - 20130919
IS - 1178-1998 (Electronic)
IS - 1176-9092 (Linking)
LA - eng
PT - Clinical Trial, Phase III
PT - Journal Article
PT - Randomized Controlled Trial
PT - Research Support, N.I.H., Extramural
PT - Research Support, N.I.H., Intramural
PT - Research Support, U.S. Gov't, Non-P.H.S
SB - IM
	106. 	Rousset S, Droit-Volet S, Boirie Y. Change in protein intake in elderly French people living at home after a nutritional information program targeting protein consumption. J.Am.Diet.Assoc. 2006;106:253-61.
Ref ID: 184
Keywords: Aged/Analysis of Variance/Anthropometry/Attitude to Health/Diet Surveys/Dietary Proteins/administration & dosage/Female/France/Health Knowledge,Attitudes,Practice/Humans/Male/Nutritional Requirements/Nutritional Sciences/education/Sex Factors/Statistics,Nonparametric/Surveys and Questionnaires
Reprint: Not in File
Abstract: OBJECTIVE: The purpose of the study was to assess the impact of a nutrition information program targeting protein consumption in elderly people. DESIGN AND INTERVENTION: Participants individually completed a questionnaire on food consumption and answered an attitude questionnaire (first survey period). Half of the participants (message group) participated in an information program targeting protein consumption, whereas the other half (control group) were not given any information. Two weeks after the program, both groups participated in the same surveys again (second survey period). SUBJECTS: Eighty-two healthy subjects (65 to 75 years old) living at home participated in this study. STATISTICAL ANALYSES: A two-way multivariate analysis of variance, paired t tests, and chi2 tests were performed to determine the influence of group (control versus message) and gender on the differences in protein consumption and in attitudes between the first and second survey periods. RESULTS: In the second survey period, the control group participants decreased their protein intake by an average of 0.049 g/lb/day, mainly by a reduction in meat product consumption. Conversely, the message group participants increased their protein intake by 0.041 g/lb/day, with a greater increase for the women (0.059) than the men (0.023 g/lb/day). After the nutrition information program, knowledge, perceived control on health, and belief that sensory perception decreased with age were higher among the message group participants. CONCLUSIONS: Nutrition knowledge and protein intake increased significantly among the message group participants. Thus, it is possible to change dietary practice and knowledge in elderly individuals by information targeting one nutrition message
Notes: DA - 20060130
IS - 0002-8223 (Print)
IS - 0002-8223 (Linking)
LA - eng
PT - Journal Article
PT - Randomized Controlled Trial
PT - Research Support, Non-U.S. Gov't
RN - 0 (Dietary Proteins)
SB - AIM
SB - IM
	107. 	Russell C, Oakland MJ. Nutrition education for older adults: the Chef Charles Club. J.Nutr Educ.Behav. 2007;39:233-4.
Ref ID: 171
Keywords: Aged/Aging/physiology/Exercise/Female/Focus Groups/Food Habits/psychology/Food Handling/instrumentation/methods/Fruit/Health Promotion/Humans/Male/Nutritional Sciences/education/Vegetables
Reprint: Not in File
Notes: DA - 20070703
IS - 1499-4046 (Print)
IS - 1499-4046 (Linking)
LA - eng
PT - Journal Article
PT - Research Support, Non-U.S. Gov't
SB - IM
	108. 	Rydwik E, Lammes E, Frandin K, Akner G. Effects of a physical and nutritional intervention program for frail elderly people over age 75. A randomized controlled pilot treatment trial. Aging Clin.Exp.Res. 2008;20:159-70.
Ref ID: 164
Keywords: Activities of Daily Living/Aged/Aged,80 and over/Body Weight/Energy Intake/Exercise Therapy/methods/Female/Frail Elderly/Humans/Male/Muscle Strength/Nutrition Therapy/Pilot Projects/Postural Balance/Sweden/Walking
Reprint: Not in File
Abstract: BACKGROUND AND AIMS: There are few studies published that combine the interventions of physical training and nutrition. The aim of the present study was to describe the impact of a physical and nutritional intervention program for frail community- dwelling elderly people over the age of 75. METHODS: Ninety-six community-dwelling elderly people (58 women) were randomized to four different groups: i) a physical training program (aerobic, muscle strength, balance), ii) a nutritional intervention program (individually targeted advice and group sessions), iii) a combination of these interventions, and iv) a control group. At baseline subjects were screened for physical performance such as muscle strength, balance, mobility and activities of daily living, as well as nutritional aspects such as energy intake, body weight and fat-free mass. These measurements were repeated immediately after the intervention, which lasted for 12 weeks, and after another 6 months. RESULTS: The intention-to-treat analysis indicated significant improvements in lower- extremity muscle strength in both training groups compared with the nutrition group at 1st follow-up. There were small significant changes for some of the balance measurements in the training group without nutrition treatment. The nutrition intervention did not show any significant results. CONCLUSIONS: This study shows the positive effect on lower-extremity muscle strength directly after the intervention. Balance training most probably needs to be more individualized in order to be effective for frail elderly people. Further studies are needed, with larger sample sizes, to investigate the effects of these types of interventions before any further conclusions can be drawn
Notes: DA - 20080423
IS - 1594-0667 (Print)
IS - 1594-0667 (Linking)
LA - eng
PT - Journal Article
PT - Randomized Controlled Trial
SB - IM
	109. 	Rydwik E, Frandin K, Akner G. Effects of a physical training and nutritional intervention program in frail elderly people regarding habitual physical activity level and activities of daily living--a randomized controlled pilot study. Arch.Gerontol.Geriatr. 2010;51:283-9.
Ref ID: 140
Keywords: Activities of Daily Living/Aged/Aged,80 and over/Exercise Therapy/methods/Female/Frail Elderly/Humans/Male/Models,Statistical/Nutrition Therapy/Pilot Projects/Treatment Outcome
Reprint: Not in File
Abstract: The aim of this randomized controlled pilot study is to describe the effects of a physical training and nutritional intervention program on the physical activity level and activities of daily living (ADL) in frail elderly people. Ninety-six community-dwelling frail elderly people (58 women) above the age of 75 were included in the study. The 12-week physical and/or nutritional intervention program was followed by six months of home-based exercises for the training groups, followed up with training diaries. At baseline the subjects were screened for physical activity level, walking habits, and ADL. These measurements were repeated immediately after the intervention at 3 months, and at 2nd follow-up at 9 months. ADL data were also collected 24 months after baseline at 3rd follow-up. The intention-to-treat analyses showed an increase of the habitual physical activity level and walking duration at 1st follow-up for the two training groups compared to the other groups. These increases remained at 2nd follow-up. The nutrition intervention did not show any significant results. No significant effects on ADL were shown however, there were moderate correlations between increases in physical activity level and ADL as well as between the amounts of home-based exercises and ADL for the two training groups
Notes: DA - 20101011
IS - 1872-6976 (Electronic)
IS - 0167-4943 (Linking)
LA - eng
PT - Journal Article
PT - Randomized Controlled Trial
SB - IM
	110. 	Sallinen J, Fogelholm M, Pakarinen A et al. Effects of strength training and nutritional counseling on metabolic health indicators in aging women. Can.J.Appl.Physiol 2005;30:690-707.
Ref ID: 182
Keywords: Aged/Blood Pressure/Body Composition/Body Mass Index/Cholesterol/blood/Cholesterol,HDL/Cholesterol,LDL/Counseling/Exercise/physiology/Female/Heart Rate/Humans/Middle Aged/Muscle,Skeletal/Nutritional Physiological Phenomena/Triglycerides
Reprint: Not in File
Abstract: PURPOSE: Effects of strength training (ST) and nutritional counseling (NC) on metabolic health indicators were examined in 50 aging women. METHODS: Subjects performed ST for 21 weeks. NC was given to obtain sufficient energy and protein intake, and recommended intake of fat and fiber. RESULTS: NC increased intake of protein and polyunsaturated fat by 4.5% and 10.7% and decreased intake of saturated fat by 18.3%. Serum concentrations of total cholesterol (TC), LDL-cholesterol (LDL-C), total and HDL-cholesterol (HDL-C) ratio and triacylglycerols (TAG) decreased, and serum HDL-C increased in all subjects after ST. Respectively, systolic and diastolic blood pressure and serum insulin concentration decreased in all subjects. NC contributed to the decreases in levels of serum LDL-C after the first half of ST and serum TC and HDL-C ratio during both ST periods. Changes in serum TAG concentrations correlated positively with intake of carbohydrates, and negatively with monounsaturated fat in all subjects. Respectively, changes in serum TC levels were related to protein intake, and changes in serum HDL-C to intake of fat, and inversely to carbohydrate and protein in all subjects. Relationships between serum TC and HDL-C levels and protein intake were only observable in the ST+NC group. CONCLUSIONS: The long-term ST had favorable effects on serum lipids, lipoproteins, insulin concentration, and blood pressure. However, NC further contributed to positive changes in serum lipids and lipoproteins
Notes: DA - 20060220
IS - 1066-7814 (Print)
IS - 1066-7814 (Linking)
LA - eng
PT - Journal Article
PT - Randomized Controlled Trial
PT - Research Support, Non-U.S. Gov't
RN - 0 (Cholesterol, HDL)
RN - 0 (Cholesterol, LDL)
RN - 0 (Triglycerides)
RN - 97C5T2UQ7J (Cholesterol)
SB - IM
	111. 	Schijndel-Speet M, Evenhuis HM, van Empelen P, van Wijck R, Echteld MA. Development and evaluation of a structured programme for promoting physical activity among seniors with intellectual disabilities: a study protocol for a cluster randomized trial. BMC.Public Health 2013;13:746.
Ref ID: 68
Keywords: Aged/Cluster Analysis/Health Promotion/methods/Humans/Intellectual Disability/Motor Activity/Program Development/Program Evaluation/Research Design
Reprint: Not in File
Abstract: BACKGROUND: Older people with intellectual disabilities have very low physical activity levels. Well designed, theory-driven and evidence-based health promotion programmes for the target population are lacking. This paper describes the design of a cluster-randomised trial for a systematically developed health promotion programme aimed at improving physical activity and increasing fitness among seniors with intellectual disabilities. METHODS AND DESIGN: The Intervention Mapping protocol was used for programme development. After defining the programme's objectives, the following behavioural techniques were selected to achieve them: Tailoring, Education, Modelling, Mirroring, Feedback, Reinforcement and Grading. With professionals and managers of provider services for people with intellectual disabilities, we translated these strategies into a structured day-activity programme, that consisted of a physical activity and an education programme. The programme will be executed in five day-activity centres in groups of eight to ten seniors during eight months, whereas seniors in five other centres receive care as usual. The physical activity level, as measured in number of steps a day, will be used as primary outcome measurement. Secondary outcome measurements include motor fitness, cardio respiratory fitness, morphological and metabolic fitness, ADL, functional deterioration and depressive symptoms. Differences in the primary and secondary outcome measures between participants and controls will be analysed using generalized estimation equations, correcting for day-activity center as cluster. DISCUSSION: This paper provides insight into the development and content of a theory-driven intervention aimed at behavioural change in a population with a low intellectual level. Its evaluation design is described. The programme's applicability to other populations is discussed
Notes: DA - 20130820
IS - 1471-2458 (Electronic)
IS - 1471-2458 (Linking)
LA - eng
PT - Journal Article
PT - Randomized Controlled Trial
PT - Research Support, Non-U.S. Gov't
SB - IM
	112. 	Shahar S, Adznam SN, Rahman SA et al. Development and analysis of acceptance of a nutrition education package among a rural elderly population: an action research study. BMC.Geriatr. 2012;12:24.
Ref ID: 95
Keywords: Adult/Aged/Aged,80 and over/Female/Health Services Research/methods/trends/Humans/Male/Middle Aged/Nutritional Requirements/Pamphlets/Patient Acceptance of Health Care/psychology/Patient Education as Topic/Program Development/Rural Population/Young Adult
Reprint: Not in File
Abstract: BACKGROUND: It is well known that older adults are often vulnerable to malnutrition. This action research was conducted to develop a nutrition education package for promoting healthy ageing and reducing risk of chronic diseases among older adults in a rural area of Malaysia. METHODS: This study was designed and conducted in three stages, including needs assessment, development of the package and analysis of acceptance among 33 older adults aged 60 years and over in rural communities, and 14 health staff members at rural health clinics. Subjects completed a questionnaire including sociodemographic factors and acceptance evaluation of the nutrition education package with respect to content, graphics and design. Data were analysed descriptively using numbers and percentages. RESULTS: A nutrition education package comprising a booklet, flipchart and placemats was developed. A total of 42.4% of the older adults expressed that the sentences in the flipchart needed to be simplified and medical terms explained. Terminology (60%), illustrations (20%) and nutrition recommendations (20%) were the aspects that prevented elderly subjects from fully understanding the booklet. Information on the placemats was easily understood by subjects. CONCLUSIONS: A well accepted nutrition education package for promoting healthy ageing and reducing risk of chronic diseases was developed that incorporated modifications based on feedback from older adult subjects and health clinic staff in a rural area. It is a tool that can effectively be used for health education in this population
Notes: DA - 20121112
IS - 1471-2318 (Electronic)
IS - 1471-2318 (Linking)
LA - eng
PT - Journal Article
PT - Research Support, Non-U.S. Gov't
SB - IM
	113. 	Sharifirad G, Najimi A, Hassanzadeh A, Azadbakht L. Application of BASNEF educational model for nutritional education among elderly patients with type 2 diabetes: improving the glycemic control. J.Res.Med.Sci. 2011;16:1149-58.
Ref ID: 92
Reprint: Not in File
Abstract: BACKGROUND: The objective of this study was to determine the effects of nutritional educational program on glycemic control of elderly patients with type 2 diabetes. METHODS: In this parallel randomized controlled educational trial, 100 diabetic elderly patients (>/=60 years) were chosen (50 in control and 50 in test group). Nutrition education based on beliefs, attitudes, subjective norms and enabling factors (BASNEF model) was conducted. Dietary intake and glycemic indices as well as the components of the BASNEF model were assessed. The four 70-minute educational sessions were conducted in one month. Three months after training intervention, questionnaire was completed again and blood tests were performed. RESULTS: Increased intake in the mean daily servings of fruits (0.91+/- 0.82 vs. 0.17+/-0.79; p < 0.001), vegetables (0.87+/-0.86 vs. 0.03+/-1; p < 0.001) and dairy (0.35+/-0.52 vs. and 0.12+/-0.76; p < 0.001) were reported in the intervention group compared to the control group (p < 0.001). The amount of fruits, vegetables and dairy increased in the intervention group at the end of the study (p < 0.001). However, it was not significantly changed in the control group. HbA1c and fasting blood sugar (FBS) levels decreased significantly in the interventional group compared to the control group (p < 0.001). Comparing the amount of FBS and HbA1c at the end of the study with the baseline measurements showed significant reduction in interventional group (p < 0.001). However, there was no significant change in control group in this regard. CONCLUSIONS: BASNEF-based nutritional educational intervention improved dietary intakes as well as glycemic control, 3 months after intervention
Notes: DA - 20120913
IS - 1735-7136 (Electronic)
IS - 1735-1995 (Linking)
LA - eng
PT - Journal Article
	114. 	Shatenstein B, Kergoat MJ, Reid I. Outcome of a Targeted Nutritional Intervention Among Older Adults With Early-Stage Alzheimer's Disease: The Nutrition Intervention Study. J.Appl.Gerontol. 2016.
Ref ID: 8
Reprint: Not in File
Abstract: A 6-month dietary intervention program was designed for community-dwelling older adults with Alzheimer's disease. Sixty-seven persons aged 70 years and above were recruited with their caregivers from six hospital memory and geriatric outpatient clinics, and allocated to intervention (n = 34 dyads) or control group (n = 33 dyads). Usual diet was assessed by a validated food frequency questionnaire and current diet by two nonconsecutive diet recalls or records corroborated by caregivers, at recruitment (T1) and exit from the study (T2). Intervention participants received targeted dietary recommendations; control participants received Canada's Food Guide leaflets. The program was assessed using paired and independent t tests and nonparametric statistics. Fat intakes increased at T2 within intervention participants (54 +/- 16 vs. 67 +/- 23 g, p = .013), and there was a tendency for higher energy, protein, and calcium intakes at T2 within this group. Proportions with adequate protein intakes almost doubled from T1 to T2 in intervention group women (p = .028) but decreased in female controls (p = .030). Longer follow-up is necessary to determine persistence of benefits
Notes: DA - 20160225
IS - 1552-4523 (Electronic)
IS - 0733-4648 (Linking)
LA - ENG
PT - JOURNAL ARTICLE
	115. 	Silva-Smith AL, Fleury J, Belyea M. Effects of a physical activity and healthy eating intervention to reduce stroke risk factors in older adults. Prev.Med. 2013;57:708-11.
Ref ID: 73
Keywords: Aged/Diet,Sodium-Restricted/Female/Food Habits/Health Education/methods/Health Promotion/Humans/Male/Middle Aged/Motivation/Motor Activity/Obesity/etiology/prevention & control/Overweight/Risk Factors/Sedentary Lifestyle/Stroke/Vegetables/Walking
Reprint: Not in File
Abstract: OBJECTIVE: To evaluate the effects of a theory-based physical activity and healthy eating intervention aimed at reducing stroke risk factors among overweight/obese and sedentary older adults. METHODS: Between 12/2009-1/2011, participants (n=69) were randomly assigned to an 8-week group motivational intervention or biweekly newsletters by mail. Physical activity (blinded pedometer, 7-day recall) body composition, theoretical mediator, and dietary (24-hour recall) variables were measured at pre-test and post-test. The physical activity and dietary outcomes are reported. RESULTS: For outcome measures, the follow-up was 90% for the intervention group (n=29) and 91% for the control group (n=34) for this sample. Statistically significant differences in the 7-day physical activity self-report were noted at post-test in the intervention group. The dietary measures were not statistically significant at post-test; however, the intervention group increased the quantity of vegetable servings. CONCLUSION: Limited efficacy testing was supported for a combined physical activity-dietary intervention, framed by a wellness-motivation theory, and designed to reduce stroke risk factors in older adults who are sedentary and overweight/obese. Limitations were identified and recommendations for additional research provided
Notes: DA - 20131016
IS - 1096-0260 (Electronic)
IS - 0091-7435 (Linking)
LA - eng
PT - Journal Article
PT - Randomized Controlled Trial
PT - Research Support, Non-U.S. Gov't
SB - IM
	116. 	Sink KM, Espeland MA, Castro CM et al. Effect of a 24-Month Physical Activity Intervention vs Health Education on Cognitive Outcomes in Sedentary Older Adults: The LIFE Randomized Trial. JAMA 2015;314:781-90.
Ref ID: 25
Keywords: Age Factors/Aged/Aged,80 and over/Dementia/epidemiology/prevention & control/Executive Function/Exercise Therapy/methods/Female/Health Education/Health Promotion/Humans/Male/Mild Cognitive Impairment/Muscle Stretching Exercises/Resistance Training/Sedentary Lifestyle/Treatment Outcome/Upper Extremity/Walking
Reprint: Not in File
Abstract: IMPORTANCE: Epidemiological evidence suggests that physical activity benefits cognition, but results from randomized trials are limited and mixed. OBJECTIVE: To determine whether a 24-month physical activity program results in better cognitive function, lower risk of mild cognitive impairment (MCI) or dementia, or both, compared with a health education program. DESIGN, SETTING, AND PARTICIPANTS: A randomized clinical trial, the Lifestyle Interventions and Independence for Elders (LIFE) study, enrolled 1635 community-living participants at 8 US centers from February 2010 until December 2011. Participants were sedentary adults aged 70 to 89 years who were at risk for mobility disability but able to walk 400 m. INTERVENTIONS: A structured, moderate-intensity physical activity program (n = 818) that included walking, resistance training, and flexibility exercises or a health education program (n = 817) of educational workshops and upper-extremity stretching. MAIN OUTCOMES AND MEASURES: Prespecified secondary outcomes of the LIFE study included cognitive function measured by the Digit Symbol Coding (DSC) task subtest of the Wechsler Adult Intelligence Scale (score range: 0-133; higher scores indicate better function) and the revised Hopkins Verbal Learning Test (HVLT-R; 12-item word list recall task) assessed in 1476 participants (90.3%). Tertiary outcomes included global and executive cognitive function and incident MCI or dementia at 24 months. RESULTS: At 24 months, DSC task and HVLT-R scores (adjusted for clinic site, sex, and baseline values) were not different between groups. The mean DSC task scores were 46.26 points for the physical activity group vs 46.28 for the health education group (mean difference, -0.01 points [95% CI, -0.80 to 0.77 points], P = .97). The mean HVLT-R delayed recall scores were 7.22 for the physical activity group vs 7.25 for the health education group (mean difference, -0.03 words [95% CI, -0.29 to 0.24 words], P = .84). No differences for any other cognitive or composite measures were observed. Participants in the physical activity group who were 80 years or older (n = 307) and those with poorer baseline physical performance (n = 328) had better changes in executive function composite scores compared with the health education group (P = .01 for interaction for both comparisons). Incident MCI or dementia occurred in 98 participants (13.2%) in the physical activity group and 91 participants (12.1%) in the health education group (odds ratio, 1.08 [95% CI, 0.80 to 1.46]). CONCLUSIONS AND RELEVANCE: Among sedentary older adults, a 24-month moderate-intensity physical activity program compared with a health education program did not result in improvements in global or domain-specific cognitive function. TRIAL REGISTRATION: clinicaltrials.gov Identifier: NCT01072500
Notes: DA - 20150826
IS - 1538-3598 (Electronic)
IS - 0098-7484 (Linking)
LA - eng
PT - Journal Article
PT - Multicenter Study
PT - Randomized Controlled Trial
PT - Research Support, N.I.H., Extramural
PT - Research Support, N.I.H., Intramural
PT - Research Support, U.S. Gov't, Non-P.H.S
SB - AIM
SB - IM
	117. 	Song HJ, Simon JR, Patel DU. Food preferences of older adults in senior nutrition programs. J.Nutr Gerontol.Geriatr. 2014;33:55-67.
Ref ID: 53
Keywords: African Americans/Aged/Aged,80 and over/Aging/European Continental Ancestry Group/Female/Food Assistance/Food Preferences/ethnology/Health Promotion/Health Services for the Aged/Home Care Services/Humans/Male/Maryland/Middle Aged/Nutrition Policy/Nutrition Surveys/Patient Compliance/Senior Centers/Sex Characteristics
Reprint: Not in File
Abstract: The Elderly Nutrition Program (ENP) is being challenged to improve the quality of meal programs. The purpose of this study was to explore how food preferences varied depending on gender and ethnic groups. A total of 2,024 participants in the ENP aged 60 years or older were interviewed. A majority of the participants were female (74.7%), served by congregate meal programs (71.7%), with the mean +/- SD age of 76.9 +/- 9.2 years. A general impression of the meals and preferences for 13 food groups (fresh fruit, chicken, soup, salad, vegetables, potatoes, meat, sandwiches, pasta, canned fruit, legumes, deli meats, and ethnic foods) were assessed. After adjusting other variables, older males were significantly more likely to prefer deli meats, meat, legumes, canned fruit, and ethnic foods compared to females. In addition, compared with African Americans, Caucasians demonstrated higher percentages of preference for 9 of 13 food groups including pasta, meat, and fresh fruit. To improve the quality of the ENP, and to increase dietary compliance of the older adults to the programs, the nutritional services require a strategic meal plan that solicits and incorporates older adults' food preferences
Notes: DA - 20140306
IS - 2155-1200 (Electronic)
LA - eng
PT - Comparative Study
PT - Journal Article
SB - IM
SB - N
	118. 	Stevens AB, Thiel SB, Thorud JL et al. Increasing the Availability of Physical Activity Programs for Older Adults: Lessons Learned From Texercise Stakeholders. J.Aging Phys.Act. 2016;24:39-44.
Ref ID: 34
Reprint: Not in File
Abstract: Many initiatives have been developed to facilitate older adults' engagement in physical activity (PA) and document its benefits. One example is Texercise, a 12-week program with a focus on increasing participants' self-efficacy. The goal of this paper is to augment the knowledgebase of PA program implementation and dissemination by elucidating the experience of Texercise implementation as perceived by multiple stakeholders. We conducted 28 semistructured stakeholder interviews and categorized the responses into four preset themes: (1) program delivery and advocacy; (2) value/merit of the program; (3) successes/challenges of offering and sustaining the program; and (4) recommendations for enhancing implementation and delivery. We identified emergent subthemes through further analysis. Many perceptions that are broadly applicable to community organizations emerged. Our findings highlight the importance of stakeholder support when embedding PA programs in communities. Furthermore, the findings are crucial to understanding underlying processes that support widespread program dissemination and sustainability
Notes: DA - 20160114
IS - 1543-267X (Electronic)
IS - 1063-8652 (Linking)
LA - eng
PT - Journal Article
SB - IM
	119. 	Stewart AL, Gillis D, Grossman M et al. Diffusing a research-based physical activity promotion program for seniors into diverse communities: CHAMPS III. Prev.Chronic.Dis. 2006;3:A51.
Ref ID: 181
Keywords: Aged/Community Health Services/organization & administration/Exercise/psychology/Female/Health Promotion/methods/Humans/Life Style/Male/San Francisco
Reprint: Not in File
Abstract: INTRODUCTION: Increasing the physical activity levels of older adults through diffusion of successful research-based programs into community settings is challenging because of differences between research and real-world settings. This project diffused the Community Healthy Activities Model Program for Seniors (CHAMPS) II, an individual-level research-based physical activity promotion program, through three community organizations to reach lower-income and minority (primarily Hispanic or Latino and African American) seniors. METHODS: Through an academic-community partnership, university staff worked with each organization to adapt the program to be appealing and effective, enable their staff and volunteers to provide the program, increase participants' physical activity, and leave sustainable programs in place. Evaluation was based on methods recommended by the Centers for Disease Control and Prevention. RESULTS: The adapted programs, referred to as CHAMPS III, differed from the original program and among organizations. Group-based components and resource guides were included and new features were added; however, individualized components were not offered because of limited resources. A total of 321 people enrolled among three organizations; there was a trend toward increased physical activity at two organizations (an estimated increase of 481 kcal/week [P = .08] and 437 kcal/week [P = .06] expended in physical activity). Evaluation revealed challenges and unexpected community-level benefits. All organizations are continuing efforts to promote physical activity for older adults. CONCLUSION: This project enabled community organizations to implement physical activity promotion programs. The overarching challenge was to retain original program features within each organization's resources yet be sustainable. Although the programs differed from the original research program, they were a catalyst for numerous community-level changes. Our findings can guide similar projects to reach underserved older adults
Notes: DA - 20060316
IS - 1545-1151 (Electronic)
IS - 1545-1151 (Linking)
LA - eng
PT - Journal Article
PT - Research Support, Non-U.S. Gov't
SB - IM
	120. 	Stewart AL, Grossman M, Bera N et al. Multilevel perspectives on diffusing a physical activity promotion program to reach diverse older adults. J.Aging Phys.Act. 2006;14:270-87.
Ref ID: 175
Keywords: Age Factors/Aged/Attitude of Health Personnel/Exercise/Female/Focus Groups/Health Knowledge,Attitudes,Practice/Health Promotion/Health Resources/supply & distribution/Health Services Accessibility/Humans/Male/Middle Aged/Minority Groups/psychology/Needs Assessment/Patient Acceptance of Health Care/San Francisco
Reprint: Not in File
Abstract: Diffusing research-based physical activity programs in underserved communities could improve the health of ethnically diverse populations. We utilized a multilevel, community-based approach to determine attitudes, resources, needs, and barriers to physical activity and the potential diffusion of a physical activity promotion program to reach minority and lower-income older adults. Formative research using focus groups and individual interviews elicited feedback from multiple community sectors: community members, task force and coalition members, administrators, service implementers, health care providers, and physical activity instructors. Using qualitative data analysis, 47 transcripts (N = 197) were analyzed. Most sectors identified needs for culturally diverse resources, promotion of existing resources, demonstration of future cost savings, and culturally tailored, proactive outreach. The program was viewed favorably, especially if integrated into existing resources. Linking sectors to connect resources and expertise was considered essential. Complexities of such large-scale collaborations were identified. These results may guide communities interested in diffusing health promotion interventions
Notes: DA - 20061108
IS - 1063-8652 (Print)
IS - 1063-8652 (Linking)
LA - eng
PT - Journal Article
PT - Research Support, Non-U.S. Gov't
SB - IM
	121. 	Strand KA, Francis SL, Margrett JA, Franke WD, Peterson MJ. Community-based exergaming program increases physical activity and perceived wellness in older adults. J.Aging Phys.Act. 2014;22:364-71.
Ref ID: 67
Keywords: Aged/Aged,80 and over/Attitude to Health/Exercise/physiology/Female/Health Promotion/methods/Humans/Intergenerational Relations/Male/Middle Aged/Motor Activity/Pilot Projects/Play Therapy/Program Development/Program Evaluation/Rural Population/Self Report/Video Games
Reprint: Not in File
Abstract: Exergaming may be an effective strategy to increase physical activity participation among rural older adults. This pilot project examined the effects of a 24-wk exergaming and wellness program (8 wk onsite exergaming, 16-wk wellness newsletter intervention) on physical activity participation and subjective health in 46 rural older adults. Sociodemographic data and self-reported physical activity were analyzed using descriptive statistics and Cochran's Q, respectively. Qualitative data were reviewed, categorized on the basis of theme, and tabulated for frequency. Increased physical activity and perceived health were the most reported perceived positive changes. Significant increases in physical activity participation were maintained among participants who were physically inactive at baseline. Best-liked features were physical activity and socialization. Findings suggest that this pilot exergaming and wellness program is effective in increasing physical activity in sedentary rural older adults, increasing socialization, and increasing subjective physical health among rural older adults
Notes: DA - 20140620
IS - 1543-267X (Electronic)
IS - 1063-8652 (Linking)
LA - eng
PT - Evaluation Studies
PT - Journal Article
PT - Research Support, U.S. Gov't, Non-P.H.S
SB - IM
	122. 	Taguchi N, Higaki Y, Inoue S, Kimura H, Tanaka K. Effects of a 12-month multicomponent exercise program on physical performance, daily physical activity, and quality of life in very elderly people with minor disabilities: an intervention study. J.Epidemiol. 2010;20:21-9.
Ref ID: 144
Keywords: Activities of Daily Living/Aged/Aged,80 and over/Case-Control Studies/Disabled Persons/rehabilitation/Exercise/physiology/Exercise Therapy/methods/Female/Humans/Japan/Male/Nursing Homes/Physical Fitness/Program Evaluation/Prospective Studies/Quality of Life
Reprint: Not in File
Abstract: BACKGROUND: Although studies suggest that exercise training improves physical performance and health-related quality of life (HRQOL) among elderly people, most of these studies have investigated relatively healthy persons. The objective of the present study was to determine the effects of a 12-month multicomponent exercise program on physical performance, daily physical activity, and HRQOL among very elderly people with minor disabilities. METHODS: The subjects consisted of 65 elders (median age: 84 years) who were certified to receive long-term care in the form of support only or Level 1 care (the lowest level of care required); 31 were allocated to the intervention group and 34 to the control group. The intervention group participated in supervised exercises once a week for 12 months and in home-based exercises. The exercise program consisted of various exercises related to flexibility, muscle strength, balance, and aerobic performance. RESULTS: After 12 months of exercise training, the intervention group had significant improvements in lower-limb strength and on the sit-and-reach test; these effects were not observed in the control group. The control group had significant decreases in grip strength, 6-minute walking distance, walking speed, and stride length; these decreases were not observed in the intervention group. No clear differences in HRQOL measurements or changes in physical activity were detected between groups. CONCLUSIONS: The 12-month multicomponent exercise program may effectively improve and maintain the physical performance of very elderly individuals with minor disabilities
Notes: DA - 20100106
IS - 1349-9092 (Electronic)
IS - 0917-5040 (Linking)
LA - eng
PT - Clinical Trial
PT - Journal Article
PT - Research Support, Non-U.S. Gov't
SB - IM
	123. 	Tan EJ, Xue QL, Li T, Carlson MC, Fried LP. Volunteering: a physical activity intervention for older adults--The Experience Corps program in Baltimore. J.Urban.Health 2006;83:954-69.
Ref ID: 178
Keywords: African Americans/Aged/Aged,80 and over/Baltimore/Female/Health Promotion/methods/Humans/Male/Middle Aged/Motor Activity/Pilot Projects/Urban Population/Volunteers
Reprint: Not in File
Abstract: There is compelling evidence supporting the benefits of increased regular physical activity in older adults. The Experience Corps program in Baltimore MD was designed in part as a community based approach to increasing physical activity that would also appeal to older adults who have historically not utilized health promotion programs. The Baltimore Experience Corps program places older volunteers in public elementary schools for 15 h a week in roles designed to improve the academic outcomes of children and, simultaneously, increase the physical, cognitive and social activity of volunteers. This paper reports on the change in physical activity levels among older adults associated with participation in the Baltimore Experience Corps. In a pilot randomized controlled evaluation, older adults were randomly assigned to Experience Corps (EC participants) or a waiting list control group. Ages ranged from 59-86 years, 96% were African American, 94% were women, and 84% had annual incomes less than $15,000. EC participants were required to serve >/=15 h a week. At follow-up after 4-8 months, an analysis of 113 randomized volunteers revealed 53% of the EC participants were more active than the previous year by self-report, as compared to 23% of the controls (p<0.01). When adjusted for age, gender and education, there was a trend toward increased physical activity in the EC participants as calculated by a kilocalorie per week increase of 40%, versus a 16% decrease in the controls (p=0.49). EC participants who reported "low activity" at baseline experienced an average 110% increase in their physical activity at follow-up. Among the controls who were in the "low activity" group at baseline, there was, on average, only a 12% increase in physical activity (p=0.03). Among those who were previously active, there was no significant difference (p=0.30). The pilot results suggest that a high intensity volunteer program that is designed as a health promotion intervention can lead, in the short-term, to significant improvements in the level of physical activity of previously inactive older adult volunteers
Notes: DA - 20060828
IS - 1099-3460 (Print)
IS - 1099-3460 (Linking)
LA - eng
PT - Journal Article
PT - Randomized Controlled Trial
PT - Research Support, Non-U.S. Gov't
SB - IM
	124. 	Taylor N. A 2-year physical activity program for sedentary older adults does not improve cognitive functioning more than a health education program [synopsis]. J.Physiother. 2016;62:115.
Ref ID: 7
Reprint: Not in File
Notes: DA - 20160408
IS - 1836-9561 (Electronic)
IS - 1836-9561 (Linking)
LA - eng
PT - Journal Article
SB - IM
	125. 	Temple B, Janzen BL, Chad K, Bell G, Reeder B, Martin L. The health benefits of a physical activity program for older adults living in congregate housing. Can.J.Public Health 2008;99:36-40.
Ref ID: 163
Keywords: Age Factors/Aged/Aged,80 and over/Exercise/physiology/Female/Health Promotion/Health Status/Humans/Male/Middle Aged/Motor Activity/Program Development/Program Evaluation/Residence Characteristics/Saskatchewan/Socioeconomic Factors
Reprint: Not in File
Abstract: BACKGROUND: In Saskatoon in 2002, as one of the key strategies for the in motion health promotion strategy, the Forever...in motion program was developed with the general goal of increasing opportunities for physical activity among older adults living in congregate housing. The three components of the program were a low-intensity exercise program, informal socialization and educational sessions. The objective of the present study was to examine whether participation in this program positively influenced participants' physical, emotional, psychological and social well-being. METHODS: A quasi-experimental, pretest/post-test design was employed to examine the impact of the program on various aspects of participant well-being. Thirty-six program participants and a comparison group of 22 non-participants from two congregate housing facilities took part in the study. The pretest was administered to the study and comparison groups before or shortly after the 12-week session commenced, and the post-test was administered after the 12-week session had concluded. Pretest and post-test assessment consisted of self-report measures of (1) vitality, (2) self-rated health, 3) mental health, (4) social functioning, (5) role limitations due to emotional problems, 6) physical activity-related knowledge, and (7) self-efficacy for exercise. A multivariate analysis of covariance (MANCOVA) was conducted using the seven post-test scores as dependent variables and the pretest scores as covariates. RESULTS: After adjusting for differences in baseline characteristics, the findings revealed statistically significant improvements in self-reported health and self-efficacy for exercise in the program participant group as compared with non-participants. CONCLUSION: The results of this study suggest that a relatively low-cost, low-intensity exercise program such as the Forever...in motion program may positively influence the well-being of older adults living in congregate housing. However, additional research with a larger number of participants and a more rigorous study design is needed to further elucidate the health benefits of the Forever...in motion program
Notes: DA - 20080425
IS - 0008-4263 (Print)
IS - 0008-4263 (Linking)
LA - eng
PT - Comparative Study
PT - Journal Article
SB - IM
	126. 	Thiel C, Vogt L, Tesky VA et al. Cognitive intervention response is related to habitual physical activity in older adults. Aging Clin.Exp.Res. 2012;24:47-55.
Ref ID: 122
Keywords: Aged/Aged,80 and over/Aging/physiology/psychology/Alzheimer Disease/diagnosis/physiopathology/therapy/Cognition/Cognition Disorders/Cognitive Therapy/methods/Female/Follow-Up Studies/Humans/Male/Memory Disorders/Motor Activity/Neuropsychological Tests/Quality of Life/Treatment Outcome
Reprint: Not in File
Abstract: BACKGROUND AND AIMS: This study analysed the associations between physical activity and the effects of cognitive training on perceived cognitive functioning and life satisfaction in older adults. METHODS: A sample of 114 intervention group participants (65-89 yrs) received weekly group sessions of cognitive stimulation for two months. This sample was stratified into groups according to habitual physical activity (PA) and matched with 45 controls. Participants completed the Memory Complaint Questionnaire (MAC-Q), Nuremberg Self-Rating List (NSL) and Alzheimer Disease Assessment Scale - Cognitive Subscale (ADAS-Cog) at three time-points (baseline, 2 months, and 6-month follow-up). RESULTS: At baseline, groups did not differ in absolute MAC-Q, NSL or ADAS-Cog scores. NSL difference scores (follow-up score minus baseline NSL score) of the three cognitive intervention groups (>6.95h MVPA/ wk; 3.64-6.95h MVPA/wk; <3.64h MVPA/wk) and controls were -3.8+/-7.3, -2.5+/-11.0, +0.3+/-12.0 and +0.1+/-9.1 over 2 months, and -4.2+/-7.6, -4.0+/-14.0, -1.8+/-7.7 and +0.5+/-9.7 over 6 months, respectively. MAC-Q difference scores were -1.1+/-2.9, -1.1+/-3.4, -0.3+/-3.9 and +0.3+/-2.7 over 2 months, and -1.5+/-3.2, -0.8+/-2.9, -0.3+/-2.9 and +0.3+/-2.2 over 6 months. The groups significantly (p<0.05) differed on NSL and MAC-Q difference scores. Specifically, the more active groups differed from controls, and in some cases from the least active group. Groups did not differ on ADAS-Cog difference scores. CONCLUSIONS: Our findings indicate a relation between amount of physical activity and the effects of a cognitive stimulation intervention on perceived cognitive functioning and life satisfaction. Physically more active persons may gain more benefit from cognitive stimulation than the physically less active
Notes: DA - 20120528
IS - 1594-0667 (Print)
IS - 1594-0667 (Linking)
LA - eng
PT - Journal Article
PT - Randomized Controlled Trial
PT - Research Support, Non-U.S. Gov't
SB - IM
	127. 	Toto PE, Raina KD, Holm MB, Schlenk EA, Rubinstein EN, Rogers JC. Outcomes of a multicomponent physical activity program for sedentary, community-dwelling older adults. J.Aging Phys.Act. 2012;20:363-78.
Ref ID: 107
Keywords: Activities of Daily Living/Aged/Aging/physiology/psychology/Analysis of Variance/Benchmarking/Depression/Female/Health Status Indicators/Humans/Male/Motor Activity/Outcome Assessment (Health Care)/Pilot Projects/Program Development/Program Evaluation/Psychometrics/Residence Characteristics/Sedentary Lifestyle/Self Report/Time Factors
Reprint: Not in File
Abstract: This single-group repeated-measures pilot study evaluated the effects of a 10-wk, multicomponent, best-practice exercise program on physical activity, performance of activities of daily living (ADLs), physical performance, and depression in community-dwelling older adults from low-income households (N = 15). Comparison of pretest and posttest scores using a one-tailed paired-samples t test showed improvement (p < .05) for 2 of 3 ADL domains on the Activity Measure-Post Acute Care and for 6 physical-performance measures of the Senior Fitness Test. Repeated-measures ANOVA revealed significant main effects for 3 of 8 physical activity measures using the Yale Physical Activity Scale. Retention rate was 78.9%, and the adherence rate for group sessions was 89.7%. Results suggest that participation in a multicomponent, best-practice physical activity program may positively affect sedentary, community-dwelling older adults' physical activity, ADL performance, and physical performance
Notes: DA - 20120625
IS - 1543-267X (Electronic)
IS - 1063-8652 (Linking)
LA - eng
PT - Comparative Study
PT - Journal Article
PT - Research Support, Non-U.S. Gov't
SB - IM
	128. 	van der DM, Etman A, Kamphuis CB, van Lenthe FJ. Participation levels of physical activity programs for community-dwelling older adults: a systematic review. BMC.Public Health 2014;14:1301.
Ref ID: 42
Keywords: Aged/Aged,80 and over/Consumer Participation/Exercise/Humans/Middle Aged/Motor Activity
Reprint: Not in File
Abstract: BACKGROUND: Although many physical activity (PA) programs have been implemented and tested for effectiveness, high participation levels are needed in order to achieve public health impact. This study aimed to determine participation levels of PA programs aimed to improve PA among community-dwelling older adults. METHODS: We searched five databases up until March 2013 (PubMed, PubMed publisher, Cochrane Library, EMBASE, and Web of Science) to identify English-written studies investigating the effect of PA programs on at least one component of PA (e.g. frequency, duration) among community-dwelling populations (i.e. not in a primary care setting and/or assisted living or nursing home) of persons aged 55 years and older. Proportions of participants starting and completing the PA programs (initial and sustained participation, respectively) were determined. RESULTS: The search strategy yielded 11,994 records of which 16 studies were included reporting on 17 PA programs. The number of participants enrolled in the PA programs ranged between 24 and 582 persons. For 12 PA programs it was not possible to calculate initial participation because the number of older adults invited to participate was unknown due to convenience sampling. Of the five remaining programs, mean initial participation level was 9.2% (+/-5.7%). Mean sustained participation level of all 17 programs was 79.8% (+/-13.2%). CONCLUSIONS: Understanding how to optimize initial participation of older adults in PA programs deserves more attention in order to improve the population impact of PA programs for community-dwelling older adults
Notes: DA - 20141226
IS - 1471-2458 (Electronic)
IS - 1471-2458 (Linking)
LA - eng
PT - Journal Article
PT - Research Support, Non-U.S. Gov't
PT - Review
SB - IM
	129. 	Van Hoecke AS, Delecluse C, Bogaerts A, Boen F. Effects of need-supportive physical activity counseling on well-being: a 2-year follow-up among sedentary older adults. J.Phys.Act.Health 2014;11:1492-502.
Ref ID: 57
Keywords: Aged/Aged,80 and over/Anxiety/epidemiology/Chronic Disease/Counseling/methods/Female/Follow-Up Studies/Health Promotion/Humans/Male/Middle Aged/Personal Autonomy/Personal Satisfaction/Self Report/Surveys and Questionnaires/Walking/psychology
Reprint: Not in File
Abstract: BACKGROUND: This study evaluated the long-term effectiveness of multiple physical activity counseling strategies on subjective health among older adults. METHODS: Sedentary older adults (n = 442) were randomized to 3 programs: (1) a one-contact referral to locally organized physical activities, (2) a one-contact provision of a walking program, (3) a 10-week multiple-contact physical activity coaching based on the Self-Determination Theory. Self-reports on well-being, trait anxiety and physical activity were completed at baseline (pretest), and 10 weeks after (10-week follow-up), 1 year after (1-year follow-up) and 2 years after (2-year follow-up) pretests. RESULTS: All 3 programs yielded improvements in well-being and trait anxiety from pretest to 10-week follow-up and to 1-year follow-up. From pretest to 2-year follow-up, no changes emerged in well-being whereas trait anxiety increased significantly. Changes over time in well-being and anxiety were not significantly different between the programs. Changes in physical activity contributed significantly to the prediction of changes in well-being and trait anxiety. CONCLUSIONS: The findings demonstrate the year-round effectiveness of physical activity counseling on subjective health among older adults, irrespective of counseling strategy. However, a relapse to baseline level occurred 2 years after the intervention. Physical activity appears to be an important determinant of older adults' well-being
Notes: DA - 20150212
IS - 1543-5474 (Electronic)
IS - 1543-3080 (Linking)
LA - eng
PT - Journal Article
PT - Randomized Controlled Trial
PT - Research Support, Non-U.S. Gov't
SB - IM
	130. 	Van Hoecke AS, Delecluse C, Bogaerts A, Boen F. The long-term effectiveness of need-supportive physical activity counseling compared with a standard referral in sedentary older adults. J.Aging Phys.Act. 2014;22:186-98.
Ref ID: 77
Keywords: Aged/Analysis of Variance/Counseling/methods/Exercise/psychology/Female/Health Promotion/Humans/Linear Models/Longitudinal Studies/Male/Motivation/Referral and Consultation/statistics & numerical data/Risk Reduction Behavior/Sedentary Lifestyle/Self Report/Social Support/Walking/physiology
Reprint: Not in File
Abstract: This study compared the long-term effectiveness of three physical activity counseling strategies among sedentary older adults: a 1-contact referral (REFER), a 1-contact individualized walking program (WALK), and multiple-contact, individually tailored, and need-supportive coaching based on the self-determination theory (COACH). Participants (n = 442) completed measurements before (pretest), immediately after (posttest), and 1 yr after (follow-up test) a 10-wk intervention. Linear mixed models demonstrated significant time-by-condition interaction effects from pre- to posttest. More specifically, WALK and COACH yielded larger increases in daily steps and self-reported physical activity than REFER. Similarly, self-reported physical activity increased more from pre- to follow-up test in WALK and COACH compared with REFER. Autonomous motivation mediated the effect of perceived need-support on physical activity, irrespective of counseling strategy. These results demonstrate the long-term effectiveness of both a 1-contact individualized walking program and a more time-consuming, need-supportive coaching, especially in comparison with a standard referral to local opportunities
Notes: DA - 20140313
IS - 1543-267X (Electronic)
IS - 1063-8652 (Linking)
LA - eng
PT - Clinical Trial
PT - Comparative Study
PT - Journal Article
PT - Research Support, Non-U.S. Gov't
SB - IM
	131. 	van Stralen MM, de Vries H, Mudde AN, Bolman C, Lechner L. The working mechanisms of an environmentally tailored physical activity intervention for older adults: a randomized controlled trial. Int.J.Behav.Nutr Phys.Act. 2009;6:83.
Ref ID: 142
Reprint: Not in File
Abstract: BACKGROUND: The aim of this study was to explore the working mechanisms of a computer tailored physical activity intervention for older adults with environmental information compared to a basic tailored intervention without environmental information. METHOD: A clustered randomized controlled trial with two computer tailored interventions and a no-intervention control group was conducted among 1971 adults aged >/= 50. The two tailored interventions were developed using Intervention Mapping and consisted of three tailored letters delivered over a four-month period. The basic tailored intervention targeted psychosocial determinants alone, while the environmentally tailored intervention additionally targeted environmental determinants, by providing tailored environmental information. Study outcomes were collected with questionnaires at baseline, three and six months and comprised total physical activity (days/week), walking (min/week), cycling (min/week), sports (min/week), environmental perceptions and use and appreciation of the interventions. RESULTS: Mediation analyses showed that changes in cycling, sports and total physical activity behaviour induced by the environmentally tailored intervention were mediated by changes in environmental perceptions. Changes in environmental perceptions did not mediate the effect of the basic tailored intervention on behaviour. Compared with the basic tailored intervention, the environmentally tailored intervention significantly improved cycling behaviour (tau = 30.2). Additionally, the tailored letters of the environmentally tailored intervention were better appreciated and used, although these differences did not mediate the intervention effect. DISCUSSION: This study gave some first indications of the relevance of environmental perceptions as a determinant of changing physical activity behaviours and the potential effectiveness of providing environmental information as an intervention strategy aimed at enhancing physical activity behaviour among older adults
Notes: DA - 20100101
IS - 1479-5868 (Electronic)
IS - 1479-5868 (Linking)
LA - eng
PT - Journal Article
	132. 	Vroege DP, Wijsman CA, Broekhuizen K et al. Dose-response effects of a Web-based physical activity program on body composition and metabolic health in inactive older adults: additional analyses of a randomized controlled trial. J.Med.Internet.Res. 2014;16:e265.
Ref ID: 43
Keywords: Activities of Daily Living/Aged/Body Composition/physiology/Exercise/Female/Health Promotion/methods/Humans/Internet/Male/Middle Aged/Risk Factors/Weight Loss
Reprint: Not in File
Abstract: BACKGROUND: Low physical activity is a major risk factor for several age-related diseases. Recently, we showed in a randomized controlled trial that a 12-week Web-based intervention (Philips DirectLife) to increase physical activity was effective in increasing physical activity levels and metabolic health in an inactive population aged 60-70 years. OBJECTIVE: The goal of this paper was to assess how many participants successfully reached the physical activity level as targeted by the intervention and what the effects of the intervention on body composition and metabolic health in these successful individuals were to provide insight in the maximum attainable effect of the intervention. METHODS: Among the 235 participants in a randomized controlled trial of the Actief en Gezond Oud (AGO) study, we assessed the effects of the intervention on metabolic parameters in those who had successfully reached their personalized physical activity target compared with the entire intervention group. Furthermore, we studied the dose-response effect of increase in physical activity on metabolic outcome within the intervention group. RESULTS: Of the intervention group, 50 of 119 (42.0%) participants successfully reached the physical activity target (corresponding to a 10% increased daily physical activity on average). This group showed markedly higher effects of the intervention compared to the entire intervention group, with greater decreases in body weight (2.74 vs 1.49 kg), waist circumference (3.74 vs 2.33 cm), insulin resistance (HOMA index: 0.23 vs 0.20), and in cholesterol/HDL ratio (0.39 vs 0.20) and Framingham risk score (0.90% vs 0.54%). We found that men compared to women were more likely to be successful. The dose-response analysis showed that there was a significant association between increase in minutes spent in moderate-to-vigorous activity and body weight loss, BMI reduction, waist circumference reduction, HDL cholesterol increasing, and cholesterol/HDL ratio lowering. CONCLUSIONS: Of the intervention group, 42.0% (50/119) reached their daily physical activity end goal, which was associated with a markedly better effect on body composition and metabolic health compared to the effect in the entire intervention group. In this population, men are more likely to be successful in increasing physical activity. Findings demonstrate that improving the effect of such physical activity interventions requires finding new ways to increase the proportion of the population reaching the targeted goal. TRIAL REGISTRATION: Dutch Trial Registry: NTR 3045; http://www.trialregister.nl/trialreg/admin/rctview.asp?TC=3045 (Archived by WebCite at http://www.webcitation.org/6KPw52dCc)
Notes: DA - 20141209
IS - 1438-8871 (Electronic)
IS - 1438-8871 (Linking)
LA - eng
PT - Journal Article
PT - Randomized Controlled Trial
PT - Research Support, Non-U.S. Gov't
SB - IM
	133. 	Walker MH, Murimi MW, Kim Y, Hunt A, Erickson D, Strimbu B. Multiple point-of-testing nutrition counseling sessions reduce risk factors for chronic disease among older adults. J.Nutr Gerontol.Geriatr. 2012;31:146-57.
Ref ID: 97
Keywords: Aged/Aged,80 and over/Chronic Disease/epidemiology/prevention & control/Diet/adverse effects/Dietetics/methods/Female/Follow-Up Studies/Geriatric Nursing/Humans/Longitudinal Studies/Louisiana/Male/Patient Compliance/Patient Education as Topic/Risk Factors/Rural Health
Reprint: Not in File
Abstract: The objectives of this study were to explore the relationships of baseline dietary intakes and frequency of attendance at point-of-testing nutrition counseling sessions to selected risk factors for chronic diseases during a 3-year intervention. This study was part of a large multidisciplinary, community-based health outreach project conducted in a rural community of northern Louisiana. Screenings, point-of-testing counseling, weekly group exercise sessions, and group nutrition education sessions were provided over a period of 3 years. Outcome variables assessed at 6-month intervals over 3 years were body mass index (BMI), systolic and diastolic blood pressure, fasting blood glucose, and total and LDL cholesterol and dietary intake. Repeated measure analysis of variance was used to investigate the impact of the frequency of counseling sessions on outcome variables. Paired t-tests were used to identify points at which significant changes occurred. A total of 159 subjects ages 65 years and older participated in this study. The majority of the participants were female (62%) and White (82%). Attending the point of testing counseling for more than two sessions was important for a significant improvement in BMI (p </= 0.001), LDL cholesterol (p </= 0.03), blood glucose (p </= 0.03), and diastolic blood pressure (p </= 0.045). Participants who attended at least three sessions had significant reductions in risk factors for obesity and related chronic diseases, underscoring the importance of follow-up sessions after health screening
Notes: DA - 20120521
IS - 2155-1200 (Electronic)
LA - eng
PT - Journal Article
PT - Research Support, U.S. Gov't, P.H.S
SB - IM
SB - N
	134. 	Wallace R, Lo J, Devine A. Tailored Nutrition Education in the Elderly Can Lead to Sustained Dietary Behaviour Change. J.Nutr Health Aging 2016;20:8-15.
Ref ID: 15
Reprint: Not in File
Abstract: OBJECTIVES: Evaluate a 4-week dementia specific nutrition education intervention to determine long term knowledge and healthy dietary behaviour changes in 72 elderly men and women. DESIGN: A mixed method design used qualitative findings to triangulate quantitative within-subject changes to determine efficacy and sustained dietary behaviour change. SETTING: Community. PARTICIPANTS: 72 independently-living individuals. INTERVENTION: 4-week dementia specific nutrition education intervention. MEASUREMENTS: Change in participant attitude, confidence, dietary patterns, cooking behaviour, and knowledge were analysed within-subjects using non-parametric repeated-measures procedures. Significance level was set at 5% (alpha = 0.05). Effect size (ES) was reported and identified as small (S), medium (M) or large (L) if a significant change was observed. RESULTS: Compared to before the nutrition education intervention participants had an increase in total knowledge (p < 0.001, ES = 0.972 (L)), consumed a greater variety of vegetables (p = 0.007, ES = 0.35 (M)), used less salt (p = 0.006, ES = -0.42 (M-L)) and increased spice use (p < 0.001, ES = 0.40 (M-L)). Participants overcame barriers to enable sustained change, held a positive view on healthy living and believed government should invest in this sector of the community. Sharing and socialisation emerged as important themes that increased program satisfaction. CONCLUSION: The dementia specific nutrition program produced a large effect in knowledge improvement from pre to post, which was retained at follow up, consolidated observational and participatory learning which produced a moderate increase in healthy dietary behaviours which participants valued and sustained
Notes: DA - 20160105
IS - 1760-4788 (Electronic)
IS - 1279-7707 (Linking)
LA - eng
PT - Journal Article
PT - Research Support, Non-U.S. Gov't
SB - IM
	135. 	Wang X, Hsu FC, Isom S et al. Effects of a 12-month physical activity intervention on prevalence of metabolic syndrome in elderly men and women. J.Gerontol.A Biol.Sci.Med.Sci. 2012;67:417-24.
Ref ID: 111
Keywords: Adiposity/Aged/Aged,80 and over/Body Weight/Female/Humans/Male/Metabolic Syndrome X/epidemiology/Motor Activity/Pilot Projects/Prevalence/Single-Blind Method/Walking
Reprint: Not in File
Abstract: BACKGROUND: There is a lack of information on whether exercise training alone can reduce the prevalence of metabolic syndrome (MetS) in elderly men and women. METHODS: This study was an ancillary to the Lifestyle Interventions and Independence for Elders Pilot Study, a four-site, single-blind, randomized controlled clinical trial comparing a 12-month physical activity (PA) intervention (N = 180) with a successful aging intervention (N = 181) in elderly (70-89 years) community-dwelling men and women at risk for physical disability. The PA intervention included aerobic, strength, and flexibility exercises, with walking as the primary mode. MetS was defined using the National Cholesterol Education Program criteria. RESULTS: There was no significant change in body weight or fat mass after either intervention. The trend of MetS prevalence over the intervention period was similar between PA and successful aging groups (p = .77). Overall, the prevalence of MetS decreased significantly from baseline to 6 months (p = .003) but did not change further from 6- to 12-month visits (p = .11). There were no group differences in any individual MetS components (p > .05 for all group by visit interactions). However, in individuals not using medications at any visit to treat MetS components, those in the PA intervention had lower odds of having MetS than those in the successful aging group during follow-up (odds ratio = 0.28, 95% confidence interval = 0.08-0.96). CONCLUSIONS: In this sample, a 12-month PA intervention did not reduce the prevalence of MetS more than a successful aging intervention, perhaps due to the large proportion of individuals taking medications for treating MetS components
Notes: DA - 20120323
IS - 1758-535X (Electronic)
IS - 1079-5006 (Linking)
LA - eng
PT - Journal Article
PT - Randomized Controlled Trial
PT - Research Support, N.I.H., Extramural
PT - Research Support, N.I.H., Intramural
PT - Research Support, Non-U.S. Gov't
SB - AIM
SB - IM
	136. 	Weiss DR, Wolfson C, Yaffe MJ, Shrier I, Puts MT. Physician counseling of older adults about physical activity: the importance of context. Am.J.Health Promot. 2012;27:71-4.
Ref ID: 89
Keywords: Aged/Attitude to Health/Counseling/Exercise/Female/Focus Groups/Humans/Male/Middle Aged/Physician-Patient Relations/Qualitative Research/Quebec
Reprint: Not in File
Abstract: PURPOSE: Physicians are encouraged to discuss physical activity with their older adult patients. Studies of physician-initiated counseling have yielded inconsistent results, perhaps because older adults' perceptions and concerns about such counseling have not been addressed. The objective of the present work was therefore to explore such perceptions and their implications. DESIGN: Qualitative study, using a grounded theory approach. Data were collected using both focus groups and semistructured interviews. SETTING: Data were collected in several settings, including a fitness center and physicians' offices. SUBJECTS: In a first sample, 56 adults aged 65 and older participated in one of six focus group sessions examining physical activity and exercise. Subsequently, 16 older adults participated in one of two focus groups comprising a second, validation sample. Individual semistructured interviews were conducted with a sample of five physicians. METHODS: Data collection and analysis took place concurrently. Transcripts were analyzed using the constant comparative method. Recruitment, data collection, and analysis were informed by grounded theory. RESULTS: Inactive older adults experiencing a health problem were more receptive than their healthy counterparts to receiving physical activity counseling from their physicians. Those who were receptive appeared to find such an intervention useful in leading to behavior change. CONCLUSION: This study suggests that physicians' efforts in physical activity counseling may have the best impact when provided in the context of a health problem
Notes: DA - 20121101
IS - 2168-6602 (Electronic)
IS - 0890-1171 (Linking)
LA - eng
PT - Journal Article
SB - T
	137. 	Wijsman CA, Westendorp RG, Verhagen EA et al. Effects of a web-based intervention on physical activity and metabolism in older adults: randomized controlled trial. J.Med.Internet.Res. 2013;15:e233.
Ref ID: 62
Keywords: Aged/Female/Humans/Internet/Male/metabolism/Middle Aged/Motor Activity/Surveys and Questionnaires/Waiting Lists
Reprint: Not in File
Abstract: BACKGROUND: Lack of physical activity leads to detrimental changes in body composition and metabolism, functional decline, and increased risk of disease in old age. The potential of Web-assisted interventions for increasing physical activity and improving metabolism in older individuals holds great promise but to our knowledge it has not been studied. OBJECTIVE: The goal of our study was to assess whether a Web-based intervention increases physical activity and improves metabolic health in inactive older adults. METHODS: We conducted a 3-month randomized, waitlist-controlled trial in a volunteer sample of 235 inactive adults aged 60-70 years without diabetes. The intervention group received the Internet program Philips DirectLife, which was directed at increasing physical activity using monitoring and feedback by accelerometer and digital coaching. The primary outcome was relative increase in physical activity measured objectively using ankle- and wrist-worn accelerometers. Secondary outcomes of metabolic health included anthropometric measures and parameters of glucose metabolism. RESULTS: In total, 226 participants (97%) completed the study. At the ankle, activity counts increased by 46% (standard error [SE] 7%) in the intervention group, compared to 12% (SE 3%) in the control group (P(difference)<.001). Measured at the wrist, activity counts increased by 11% (SE 3%) in the intervention group and 5% (SE 2%) in the control group (P(difference)=.11). After processing of the data, this corresponded to a daily increase of 11 minutes in moderate-to-vigorous activity in the intervention group versus 0 minutes in the control group (P(difference)=.001). Weight decreased significantly more in the intervention group compared to controls (-1.5 kg vs -0.8 kg respectively, P=.046), as did waist circumference (-2.3 cm vs -1.3 cm respectively, P=.036) and fat mass (-0.6% vs 0.07% respectively, P=.025). Furthermore, insulin and HbA1c levels were significantly more reduced in the intervention group compared to controls (both P<.05). CONCLUSIONS: This was the first study to show that in inactive older adults, a 3-month Web-based physical activity intervention was effective in increasing objectively measured daily physical activity and improving metabolic health. Such Web-based interventions provide novel opportunities for large scale prevention of metabolic deregulation in our rapidly aging population
Notes: DA - 20131107
IS - 1438-8871 (Electronic)
IS - 1438-8871 (Linking)
LA - eng
PT - Journal Article
PT - Randomized Controlled Trial
PT - Research Support, Non-U.S. Gov't
SB - IM
	138. 	Wilcox S, Dowda M, Griffin SF et al. Results of the first year of active for life: translation of 2 evidence-based physical activity programs for older adults into community settings. Am.J.Public Health 2006;96:1201-9.
Ref ID: 179
Keywords: Aged/Aged,80 and over/Behavior Therapy/Body Image/Body Mass Index/Choice Behavior/Community Health Services/methods/organization & administration/Counseling/Evidence-Based Medicine/Exercise/psychology/Female/Health Promotion/Humans/Male/Middle Aged/Outcome Assessment (Health Care)/Personal Satisfaction/Program Evaluation/Self Efficacy/Social Perception/Surveys and Questionnaires
Reprint: Not in File
Abstract: OBJECTIVES: Translating efficacious interventions into practice within community settings is a major public health challenge. We evaluated the effects of 2 evidence-based physical activity interventions on self-reported physical activity and related outcomes in midlife and older adults. METHODS: Four community-based organizations implemented Active Choices, a 6-month, telephone-based program, and 5 implemented Active Living Every Day, a 20-week, group-based program. Both programs emphasize behavioral skills necessary to become more physically active. Participants completed pretest and posttest surveys. RESULTS: Participants (n=838) were aged an average of 68.4 +/-9.4 years, 80.6% were women, and 64.1% were non-Hispanic White. Seventy-two percent returned posttest surveys. Intent-to-treat analyses found statistically significant increases in moderate-to-vigorous physical activity and total physical activity, decreases in depressive symptoms and stress, increases in satisfaction with body appearance and function, and decreases in body mass index. CONCLUSIONS: The first year of Active for Life demonstrated that Active Choices and Active Living Every Day, 2 evidence-based physical activity programs, can be successfully translated into community settings with diverse populations. Further, the magnitudes of change in outcomes were similar to those reported in the efficacy trials
Notes: DA - 20060628
IS - 1541-0048 (Electronic)
IS - 0090-0036 (Linking)
LA - eng
PT - Journal Article
PT - Research Support, Non-U.S. Gov't
SB - AIM
SB - IM
	139. 	Wolff JK, Warner LM, Ziegelmann JP, Wurm S. What do targeting positive views on ageing add to a physical activity intervention in older adults? Results from a randomised controlled trial. Psychol.Health 2014;29:915-32.
Ref ID: 54
Keywords: Aged/Aging/psychology/Attitude/Female/Follow-Up Studies/Health Promotion/methods/Humans/Male/Motor Activity/Outcome Assessment (Health Care)/Sedentary Lifestyle
Reprint: Not in File
Abstract: OBJECTIVE: Physical activity is a key factor for healthy ageing, yet many older people lead a sedentary lifestyle. Traditional physical activity interventions do not consider the specific needs and views of older adults. As views on ageing are known to be related to health behaviours, the current study evaluates the effectiveness of prompting positive views on ageing within a physical activity intervention. DESIGN: Randomised controlled trial with three groups aged 65+: Intervention for physical activity with 'views-on-ageing'-component (n = 101; IGVoA), and without 'views-on-ageing'-component (n = 30; IG), and active control intervention for volunteering (n = 103; CG). MAIN OUTCOME MEASURES: Attitudes towards older adults and physical activity were assessed five weeks before intervention, two weeks, six weeks and 8.5 months after the intervention. RESULTS: Compared to the IG and CG, positive attitudes towards older adults increased in the IGVoA after the intervention. For IGVoA, the indirect intervention effect on change in activity via change in attitudes towards older adults was reliable. CONCLUSION: A 'views-on-ageing'-component within a physical activity intervention affects change in physical activity via change in views on ageing. Views on ageing are a promising intervention technique to be incorporated into future physical activity interventions for older adults
Notes: DA - 20140512
IS - 1476-8321 (Electronic)
IS - 0887-0446 (Linking)
LA - eng
PT - Journal Article
PT - Randomized Controlled Trial
PT - Research Support, Non-U.S. Gov't
SB - IM
	140. 	Wunderlich S, McKinnon C, Piemonte J, Ahmad ZN. Measuring the impact of nutrition education and physical activity on older adults participating in government sponsored programs. J.Nutr Elder. 2009;28:255-71.
Ref ID: 148
Keywords: Aged/Aging/physiology/Blood Pressure/Cohort Studies/Exercise/Female/Health Education/methods/Health Promotion/Health Status/Humans/Longitudinal Studies/Male/Nutrition Assessment/Nutritional Sciences/education
Reprint: Not in File
Abstract: A longitudinal, four-year study (2004-2007) with a cohort of 139 older adults (majority women;71%) was conducted to examine the impact of community-based nutrition and physical activity programs on health outcome measures. Demographic and anthropometric data were collected and nutrition screening was performed. Blood pressure, serum cholesterol and glucose levels, and pulse rate were also measured. The blood pressure, both mean systolic and diastolic, 141(+/- 19.0)/79.3 +/- 9.7 (2004) vs. 127.8 +/- 10.9/73.8 +/- 10.2 mm Hg (2007), were improved (p < 0.05) in women. There were also improvements (p < 0.05 in both men and women) in pulse rate between 2004 and 2007. There is no doubt that nutrition education and exercise programs together enhanced the overall health and well being of these older adults. However, our findings emphasize the need for further systematic study and for appropriate biometric assessments to evaluate the full impact of nutrition education and physical activity interventions in older adults
Notes: DA - 20101224
IS - 1540-8566 (Electronic)
IS - 0163-9366 (Linking)
LA - eng
PT - Journal Article
SB - IM
	141. 	Wyers CE, Reijven PL, Evers SM et al. Cost-effectiveness of nutritional intervention in elderly subjects after hip fracture. A randomized controlled trial. Osteoporos.Int. 2013;24:151-62.
Ref ID: 96
Keywords: Aged/Aged,80 and over/Body Weight/Cost-Benefit Analysis/Counseling/economics/methods/Dietary Supplements/Elder Nutritional Physiological Phenomena/physiology/Female/Fracture Fixation/rehabilitation/Health Care Costs/statistics & numerical data/Hip Fractures/Humans/Male/Middle Aged/Netherlands/Nutritional Support/Postoperative Care/Quality-Adjusted Life Years
Reprint: Not in File
Abstract: Hip fracture patients can benefit from nutritional supplementation during their recovery. Up to now, cost-effectiveness evaluation of nutritional intervention in these patients has not been performed. Costs of nutritional intervention are relatively low as compared with medical costs. Cost-effectiveness evaluation shows that nutritional intervention is likely to be cost-effective. INTRODUCTION: Previous research on the effect of nutritional intervention on clinical outcome in hip fracture patients yielded contradictory results. Cost-effectiveness of nutritional intervention in these patients remains unknown. The aim of this study was to evaluate cost-effectiveness of nutritional intervention in elderly subjects after hip fracture from a societal perspective. METHODS: Open-label, multi-centre randomized controlled trial investigating cost-effectiveness of intensive nutritional intervention comprising regular dietetic counseling and oral nutritional supplementation for 3 months postoperatively. Patients allocated to the control group received care as usual. Costs, weight and quality of life were measured at baseline and at 3 and 6 months postoperatively. Incremental cost-effectiveness ratios (ICERs) were calculated for weight at 3 months and quality adjusted life years (QALYs) at 6 months postoperatively. RESULTS: Of 152 patients enrolled, 73 were randomized to the intervention group and 79 to the control group. Mean costs of the nutritional intervention was 613 Euro. Total costs and subcategories of costs were not significantly different between both groups. Based on bootstrapping of ICERs, the nutritional intervention was likely to be cost-effective for weight as outcome over the 3-month intervention period, regardless of nutritional status at baseline. With QALYs as outcome, the probability for the nutritional intervention being cost-effective was relatively low, except in subjects aged below 75 years. CONCLUSION: Intensive nutritional intervention in elderly hip fracture patients is likely to be cost-effective for weight but not for QALYs. Future cost-effectiveness studies should incorporate outcome measures appropriate for elderly patients, such as functional limitations and other relevant outcome parameters for elderly
Notes: DA - 20130104
IS - 1433-2965 (Electronic)
IS - 0937-941X (Linking)
LA - eng
PT - Journal Article
PT - Multicenter Study
PT - Randomized Controlled Trial
PT - Research Support, Non-U.S. Gov't
SB - IM
	142. 	Zhu H, An R. Impact of home-delivered meal programs on diet and nutrition among older adults: a review. Nutr Health 2013;22:89-103.
Ref ID: 49
Keywords: Aged/Aged,80 and over/Diet/Energy Intake/Food Services/Homebound Persons/Humans/Long-Term Care/Middle Aged/Nutritional Requirements/Nutritional Status/Quality of Life/Randomized Controlled Trials as Topic
Reprint: Not in File
Abstract: BACKGROUND: Poor diet quality and insufficient nutrient intake is of particular concern among older adults. The Older Americans Act of 1965 authorizes home-delivered meal services to homebound individuals aged 60 years and older. OBJECTIVE: The purpose of this study was to review scientific evidence on the impact of home-delivered meal services on diet and nutrition among recipients. METHODS: Keyword and reference searches were conducted in Cochrane Library, Google Scholar, PubMed and Web of Science. Inclusion criteria included: study design (randomized controlled trials, cohort studies, pre-post studies, or cross-sectional studies); main outcome (food and nutrient intakes); population (home-delivered meal program participants); country (US); language (articles written in English); and article type (peer-reviewed publications or theses). RESULTS: Eight studies met the inclusion criteria, including two randomized controlled trial studies (from the same intervention), one cohort study, two pre-post studies, and three cross-sectional studies. All but two studies found home-delivered meal programs to significantly improve diet quality, increase nutrient intakes, and reduce food insecurity and nutritional risk among participants. Other beneficial outcomes include increased socialization opportunities, improvement in dietary adherence, and higher quality of life. CONCLUSIONS: Home-delivered meal programs improve diet quality and increase nutrient intakes among participants. These programs are also aligned with the federal cost-containment policy to rebalance long-term care away from nursing homes to home- and community-based services by helping older adults maintain independence and remain in their homes and communities as their health and functioning decline
Notes: DA - 20150330
IS - 0260-1060 (Print)
IS - 0260-1060 (Linking)
LA - eng
PT - Journal Article
PT - Review
SB - IM
