# Supplementary material for: Intervention Mapping: A Framework to Co‐Design the ALAPAGE Programme to Simultaneously Improve Dietary Diversity and Physical Fitness Among Older People
Source: Health Expect. 2026 Mar 23;29(2):e70612. doi: 10.1111/hex.70612 (PMC13087432; doi:10.1111/hex.70612)
Supplement: Supplementary file 4 — Supporting file 4: Interview guide. [file HEX-29-e70612-s002.pdf]

Interview guide

Focus group guide

Presentation of the framework

Interviewer's script: "Hello. Thank you for agreeing to this interview.

We are working on a project called ALAPAGE, which is an action research project on nutrition and physical activity among seniors. As part of this project, we would like to hear your opinions and impressions of the workshops you have participated in. The aim is for you to share your ideas and help us improve these workshops. The purpose of the sessionis to express yourself as freely as possible; there are no right or wrong answers.

Everything you say is interesting. There is no judgement, we are here only to listen to you. Everything said here will remain confidential. The data we collect will remain anonymous. The interview will be recorded with your consent, and you can request that your data be removed from the study at any time.

Participation/Membership in the workshops

Question 1: Can you tell us what motivated you to participate in the workshops?

- How did you find out about the workshops?

- Did you encounter any difficulties in attending the workshops (equipment and organisational conditions in your life)?

Question 2: Can you tell us what the barriers and facilitators are to participating in the workshops?

- Do you know anyone who has heard about the workshops but has not registered or who has hesitated?

- Do you know anyone who signed up but didn't attend? Why not?

Question 3: Do you have any ideas on how to attract more participants?

- What do you think about making initial contact at home to encourage participation (e.g. senior

ambassadors)?

- What do you think about the role of healthcare professionals in referring people to the workshops?

Perception of the workshops and behavioural changes

Question 4: Can you tell us about the workshops, give us your impressions and say what you got out of them?

- Has your perception of what constitutes a balanced diet or regular physical activity changed as a result of the workshops? How did you notice this? Can you give us an example?

Question 5: Have you changed your eating habits and/or shopping habits,

or your physical activity? Can you give us an example?

- What has encouraged these changes and what are the obstacles?

- What could help you maintain these changes over time?

- Do you think professionals could help you maintain these changes?

New technologies

Question 6: What would you think about using new technologies to create games in the workshops?

- What are the obstacles to using new technologies?

Question 7: Do you think that using new technologies could help you maintain changes after the workshops?

Interview guide (non-directive)

Presentation of the framework

"Hello. Thank you for agreeing to this interview. The data collected is anonymous and confidential and will only be used for my dissertation. With your consent, the interview will be recorded, and you can request that thedata be removed from the study at any time.

This interview focuses on the motivations for participating in prevention workshops for senior citizens. I am a student in the humanities and I am working on a study that aims to improve nutrition/physical activity prevention workshops for senior citizens. These workshops consist of several sessions that provide tips for maintaining regular physical activity and a balanced diet. We would like to hear your thoughts on what would motivate you to participate in these workshops.

The goal is to express whatever comes to mind as freely as possible, without any restrictions.

There are no right or wrong answers; I am not here to confirm or deny what you say. Everything you express will be interesting.

Initiative instructions

Can you tell me what would motivate you to participate in nutrition/physical activity workshops and what would prevent you from participating in nutrition/physical activity workshops?

Sociodemographic data

Gender: Male/Female

Age:

Occupation before retirement:

Mode of transport:

Do you live alone?

Yes/No

Interview guide (semi-structured)

Presentation of the framework

"Hello. Thank you for agreeing to this interview. The data collected is anonymous and confidential and will only be used for my dissertation. With your consent, the interview will be recorded, and you may request that the data be removed from the study at any time.

This interview focuses on the motivations for participating in prevention workshops for senior citizens. I am a student in the humanities and am working on a study that aims to improve nutrition/physical activity prevention workshops for senior citizens. These workshops consist of several sessions that provide tips on how to maintain regular physical activity and a balanced diet. We would like to hear your thoughts on what might motivate you to participate in these workshops.

The aim is to express as freely as possible whatever comes to mind, without any restrictions.

There are no right or wrong answers; I am not here to confirm or deny what you say, everything you express will be interesting.

Initiative instructions

Can you tell me what would motivate you to participate in nutrition/physical activity workshops and what would prevent you from participating in nutrition/physical activity workshops

?

Follow-up questions:

Question 1: What do you think about the value of acquiring knowledge and skills about nutrition or physical activity, or testing your personal knowledge during these workshops?

Question 2: What do you think about offering practical exercises and a variety of activities (e.g. tasting, developing a recipe, trying different types of sport) during these workshops? How else could you feel active?

84

Question 3: How useful do you think it is to adapt to everyone's needs and abilities during these workshops? How do you think this could be done?

Question 4: In your opinion, what qualities should the facilitator have to make you want to participate?

Question 5: In your opinion, can old eating habits, taught by previous generations, be an obstacle to maintaining a balanced diet and participating in nutrition workshops?

Question 6: What do you think about the opportunity to meet and share a friendly moment with other participants during these workshops?

Question 7: In your opinion, is feeling lonely an obstacle to maintaining a balanced diet and taking an interest in nutrition?

Question 7: In your opinion, is feeling lonely an obstacle to maintaining a balanced diet and taking an interest in nutrition?

Socio-demographic data

Gender: Male / Female

Age

Occupation before retirement

Mode of transport

Do you live alone? Yes / No
