# Supplementary figures and images for: Intervention Mapping: A Framework to Co‐Design the ALAPAGE Programme to Simultaneously Improve Dietary Diversity and Physical Fitness Among Older People
Source: Health Expect. 2026 Mar 23;29(2):e70612. doi: 10.1111/hex.70612 (PMC13087432; doi:10.1111/hex.70612)

**Additional file 3: Example of material with a graphic chart: The diversity magnet**

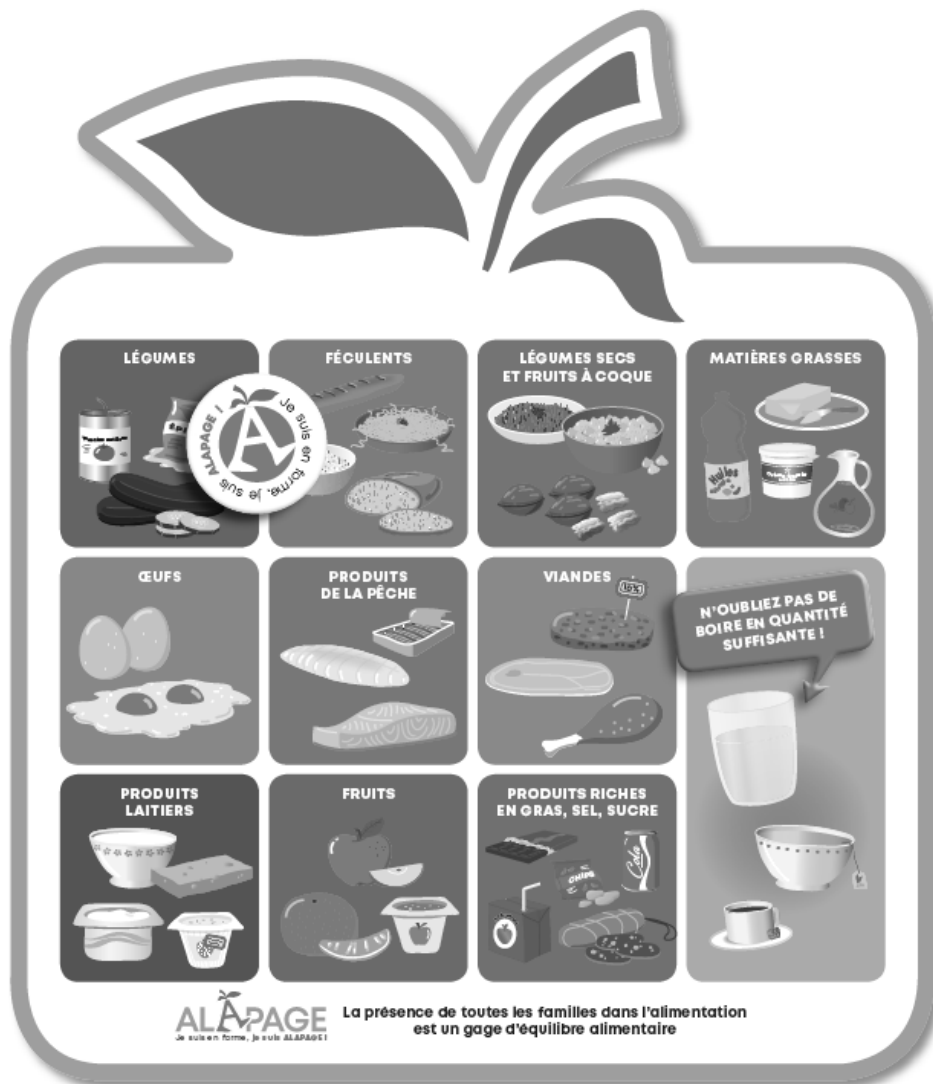

Supplement: Supplementary file 5 — Supporting file 5: Example of material with a graphic chart: The diversity magnet. [file HEX-29-e70612-s001.pdf]
